# Total Synthesis of the Marine Cyclic Depsipeptide Lagunamide D

**DOI:** 10.3390/md23030099

**Published:** 2025-02-24

**Authors:** Huiru Nan, Xiong-En Long, Jianfei He, Hailiang Xing, Min-Jing Cheng, Jin-Bao Peng, Tao Ye, Jia-Lei Yan, Junyang Liu

**Affiliations:** 1 School of Pharmacy and Food Engineering, Wuyi University, Jiangmen 529020, China; huiru_nan@foxmail.com (H.N.); 18255186096@163.com (H.X.); pengjb_05@126.com (J.-B.P.); 2 State Key Laboratory of Chemical Oncogenomics, Key Laboratory of Chemical Genomics, Peking University Shenzhen Graduate School, Shenzhen, 518055, China; 3 Qian Yan (Shenzhen) Pharmatech. Ltd., Shenzhen 518172, China; 4 Center for Bioactive Natural Molecules and Innovative Drugs, and Guangdong Province Key Laboratory of Pharmacodynamic Constituents of TCM and New Drugs Research, College of Pharmacy, Jinan University, Guangzhou 510632, China; chengmj1235@jnu.edu.cn

**Keywords:** lagunamide D, marine natural products, depsipeptide, total synthesis

## Abstract

Lagunamide D is a structurally distinct 26-membered cytotoxic cyclic depsipeptide, originally isolated from a marine cyanobacterium. It exhibits potent antiproliferative activity in the low nanomolar range against A549 human lung adenocarcinoma cells and HCT116 colon cancer cells. A significant challenge associated with lagunamide D is its propensity for intramolecular acyl migration, which leads to the formation of a contracted 24-membered analog, lagunamide D′. This structural rearrangement complicates its isolation, characterization, and synthesis. In this study, the total synthesis of lagunamide D was achieved in a 14-step longest linear sequence, starting from the known intermediate **17**, with an overall yield of 4.6%. The synthetic strategy involved several key transformations, including Ghosh’s TiCl_4_-promoted anti-aldol reaction, Corey–Bakshi–Shibata reduction (CBS reduction), cross-metathesis, Pinnick oxidation, and Yamaguchi esterification. Furthermore, this synthetic effort unambiguously confirmed the stereochemistry of the natural product.

## 1. Introduction

Natural products and their derivatives have long been recognized as invaluable sources of pharmaceuticals, playing a pivotal role in drug discovery and development. Many modern medicines are derived from natural compounds, demonstrating their significant contribution to human health [1]. While terrestrial plants, animals, and microorganisms have traditionally served as primary sources for natural product-based drugs, marine-derived compounds have garnered increasing attention due to their exceptional chemical diversity, unique structural features, and promising pharmacological potential [2,3,4]. The harsh and competitive marine environment has driven the evolution of highly specialized secondary metabolites with novel mechanisms of action, making them particularly attractive candidates for pharmaceutical development.

Several marine-derived drugs have already received regulatory approval for the treatment of various diseases, including cancer, chronic pain, and neurodegenerative disorders such as Alzheimer’s disease. Notable examples include Cytarabine (Cytosar-U^®^), an anti-leukemic agent; Trabectedin (Yondelis^®^), used in soft tissue sarcoma and ovarian cancer; Ziconotide (Prialt^®^), a potent analgesic; Eribulin mesylate (Halaven^®^), a microtubule inhibitor for metastatic breast cancer; Plitidepsin (Aplidin^®^), with antiviral and anticancer properties; Brentuximab vedotin (Adcetris^®^), an antibody–drug conjugate for lymphomas; and Omega-3-Acid ethyl esters (Lovaza^®^), used in cardiovascular disease management [5]. Beyond these approved drugs, more than 20 marine-derived compounds are currently progressing through various phases of clinical trials, further highlighting their potential in therapeutic applications [6,7,8].

Despite their immense pharmaceutical promise, the development of marine natural products faces significant challenges, particularly regarding their limited natural availability. Many bioactive compounds are produced in minute quantities by marine organisms, making large-scale extraction impractical and unsustainable. To address this issue, researchers have explored alternative strategies such as total chemical synthesis, semi-synthesis, and microbial fermentation to ensure a reliable supply. While biosynthetic technologies, including genetic engineering and heterologous expression, offer promising avenues for sustainable production, these approaches also present technical and logistical challenges [9]. Continued advancements in synthetic methodologies and biotechnological innovations are therefore crucial for unlocking the full potential of marine-derived pharmaceuticals.

**Figure 1:**
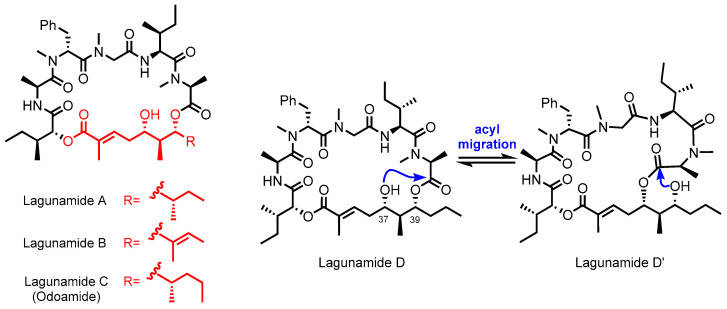
Structures of lagunamides A–D and D′.

Lagunamide D, a novel cytotoxic cyclic depsipeptide, was first isolated in 2019 by Luesch’s research group from a marine cyanobacterium collected at Loggerhead Key in Dry Tortugas, Florida. The compound was obtained in an extremely low isolation yield of 0.003% [10], highlighting the challenge of natural product extraction. Lagunamide D represents a new member of the lagunamide family, expanding the structural diversity within this class of bioactive molecules [11,12] (Figure 1).

The planar structure of lagunamide D was elucidated through a comprehensive analysis of 1D and 2D nuclear magnetic resonance (NMR) spectra, complemented by high-resolution mass spectrometry (HRMS) data. Notably, the absolute stereochemistry of the hydroxyl groups at positions C-37 and C-39 within the polyketide segment was determined using a modified Mosher method. Meanwhile, the configuration of the methyl group at position C-38 was deduced through a combination of Kishi’s NMR database [13,14] and detailed comparisons of hydrogen peak shapes and splitting patterns in the ^1^H NMR spectrum with those observed in lagunamide A. These rigorous structural studies provided valuable insight into the stereochemical framework of lagunamide D. A particularly intriguing observation was made during high-performance liquid chromatography (HPLC) separation, where intramolecular ester exchange led to the formation of a cyclization product, lagunamide D′. This transformation involved acyl migration within the macrocyclic core, converting the native 26-membered macrocycle into a contracted 24-membered analog [10] (Figure 1). Such structural rearrangements underscore the dynamic nature of cyclic depsipeptides and pose additional considerations for their synthetic and biological studies. In terms of biological activity, lagunamide D exhibits potent antiproliferative effects against human cancer cell lines. It demonstrates strong cytotoxicity towards A549 human lung adenocarcinoma cells (IC_50_ = 7.1 ± 1.7 nM) and HCT116 colon cancer cells (IC_50_ = 5.1 nM) [15], suggesting its potential as a promising lead compound for anticancer drug development. Given this remarkable bioactivity and its complex structural framework, we aim to undertake a total synthesis of lagunamide D. This effort will not only confirm its stereochemistry with absolute certainty but also provide a sustainable and scalable synthetic route to facilitate further medicinal chemistry investigations.

The key synthetic challenges associated with the lagunamide family primarily involve the construction of the anti-anti-syn configuration at the C37–C40 stereogenic centers and the incorporation of the unsaturated ester subunit within the fatty acid fragment (highlighted in red, Figure 1). In 2012, Ye and co-workers reported the first total synthesis and stereochemical revision of lagunamide A [16], highlighting the use of a double asymmetric Brown crotylboration to establish the required anti-anti-syn configuration of the C37–C40 stereogenic centers, as well as the Horner–Wadsworth–Emmons (HWE) reaction to install the α,β-unsaturated carboxylic acid unit within the polyketide chain. Lin’s group achieved the second total synthesis of lagunamide A [17], employing an asymmetric Paterson anti-aldol condensation to establish the C38–C39 stereogenic centers, followed by stereoselective allylation to install the required C37 chiral center. Furthermore, the α,β-unsaturated carboxylic acid unit was constructed via a cross-metathesis (CM) reaction. In 2018, Kazmaier and co-workers reported the third total synthesis of lagunamide A [18], utilizing a sequence of six iterative Matteson homologation steps and subsequent oxidation to generate the C37–C40 stereogenic centers. This was followed by the installation of the α,β-unsaturated ester unit through an HWE reaction. In 2016, Oishi et al. reported the first total synthesis of odoamide (lagunamide C) [19,20], featuring the use of an Evans syn-aldol reaction to establish the C39–C40 stereocenters, followed by a substrate-controlled Mukaiyama aldol reaction and a subsequent Mitsunobu reaction to install the C37 chiral center and the α,β-unsaturated ester. Currently, no reports on the total synthesis of lagunamide D exist. For the total synthesis of lagunamide D, we envisioned that the C38–C39 chiral centers could be established through an alternative anti-aldol reaction, such as the TiCl_4_-promoted protocol developed by Ghosh, while the C37 stereocenter could be installed via asymmetric reduction of the corresponding ketone. To the best of our knowledge, this approach has not been employed in previous lagunamide syntheses.

**Scheme 1:**
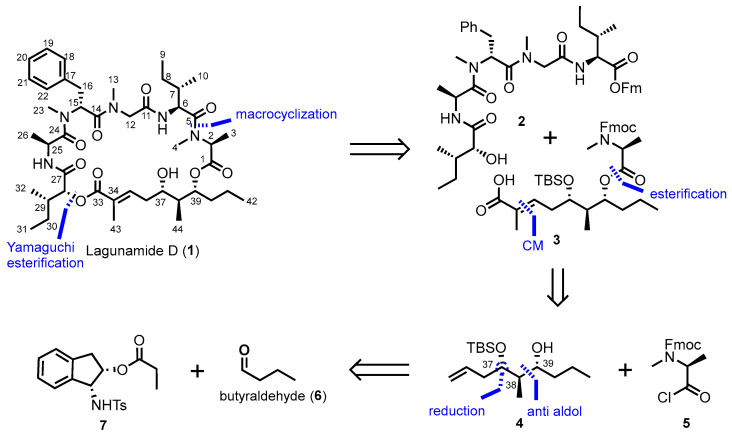
Retrosynthetic analysis of lagunamide D (**1**).

Our retrosynthetic analysis is illustrated in Scheme 1. The key transformation in our strategy is the macrocyclization between ʟ-Ile and *N*-Me-ʟ-Ala, which serves as the pivotal step in constructing the macrocyclic core of the target molecule. Following this, we implemented a strategic disconnection at the ester bond linking the α-hydroxy group of the 2-hydroxy-3-methylpentanoic acid moiety and the unsaturated acid. This approach effectively simplifies the molecule into two key intermediates: intermediate **2** and the polyketide subunit **3**, both possessing a comparable level of complexity. The formation of the ester bond in fragment **3** can be efficiently achieved through the reaction of an acyl chloride derivative with the corresponding alcohol. Meanwhile, the synthesis of the unsaturated acid is planned via a cross-metathesis reaction [21,22], enabling fragment **3** to be derived from a simpler precursor, the alcohol fragment **4**. The construction of fragment **4** is envisioned through a highly stereoselective route. Specifically, it can be assembled from intermediates **6** and **7** utilizing Ghosh’s TiCl₄-promoted anti-aldol reaction [23], which plays a crucial role in establishing the stereogenic centers at C38 and C39 with high selectivity. Furthermore, the stereogenic center at C37 will be introduced through a stereoselective reduction of the corresponding ketone, ensuring precise control over the molecule’s three-dimensional architecture. This retrosynthetic plan strategically leverages well-established reactions to achieve efficient and selective bond formations, paving the way for a streamlined synthesis of the target molecule.

## 2. Results and Discussion

**Scheme 2:**
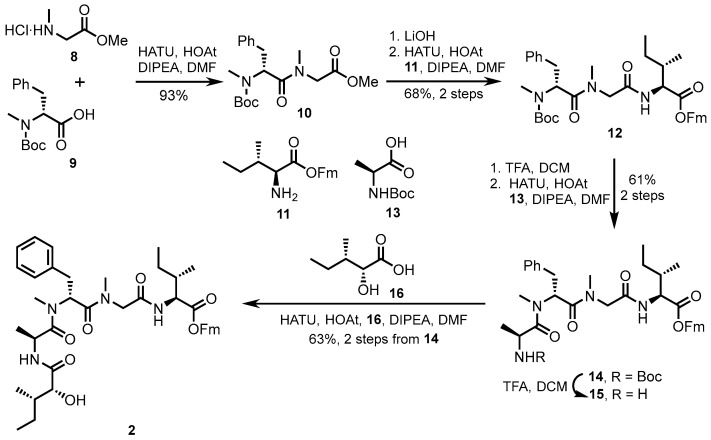
Synthesis of pentapeptide **2**.

The synthesis began with the preparation of the linear pentapeptide compound **2**, as depicted in Scheme 2. The process started with the coupling of *N*-Me-Gly-OMe·HCl (**8**) and Boc-*N*-Me-ᴅ-Phe-OH (**9**) using HATU(2-(7-Azabenzotriazol-1-yl)-*N*,*N*,*N′*,*N′*-tetramethyluronium hexafluorophosphate)/HOAt(3H-[1,2,3]-Triazolo [4,5-b]pyridin-3-ol)/DIPEA(*N*,*N*-Diisopropylethylamine)/DMF(*N*,*N*-Dimethylformamide) as the reaction conditions. This step efficiently produced the dipeptide **10** in an excellent yield of 93%. Next, the dipeptide **10** underwent hydrolysis with LiOH, converting the ester into a carboxylic acid. The resulting acid was then subjected to peptide coupling with amine **11** under similar conditions, affording the tripeptide **12** in an overall yield of 68% over two steps. Subsequent treatment of **12** with TFA (Trifluoroacetic acid) removed the Boc protecting group, generating a free amine. This intermediate was then coupled with Boc- ʟ-Ala-OH (**13**), forming the tetrapeptide **14** with a moderate yield of 61% over two steps. Finally, Boc deprotection of **14** using TFA yielded the corresponding amine **15**, which was coupled with α-hydroxy acid **16** under the same peptide coupling conditions. This step completed the synthesis of the pentapeptide **2** with an overall yield of 63% over the last two steps.

**Scheme 3:**
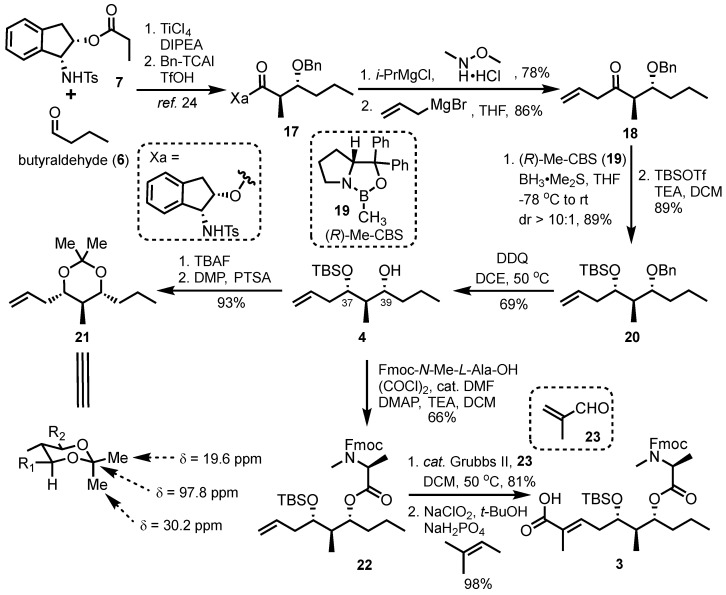
Synthesis of carboxylic acid **3**.

Upon successfully synthesizing pentapeptide **2**, we shifted our focus to obtaining the polyketide fragment **3**, as depicted in Scheme 3. Following established literature procedures [24], the anti-aldol product **17** was synthesized from butanal (**6**) under Ghosh’s TiCl_4_-promoted anti-aldol conditions [23]. This was followed by benzyl (Bn) protection using the Bn-TCAI (Benzyl 2,2,2-trichloroacetimidate) reagent and a catalytic amount of TfOH. Treatment of compound **17** with isopropyl magnesium chloride and *N*,*O*-dimethylhydroxylamine hydrochloride yielded the Weinreb amide in a moderate yield of 78%. Subsequent reaction with allyl magnesium bromide led to the formation of ketone **18**. Stereoselective reduction of ketone **18** was crucial to obtaining the desired 1,3-syn hydroxyl substrate. Initially, we adopted conditions previously used in the total synthesis of des-thiomethyllooekeyolide A [25], employing methanol as the solvent and NaBH_4_ as the reducing agent at −30 °C. However, the stereoselectivity was inconsistent, ranging from 5:1 to 7:1 diastereomeric ratio (dr). Other conditions were tested, including the following: MeOH/NaBH_4_ at −60 °C and −78 °C, MeOH/NaBH_4_/CeCl_3_·7H_2_O at −60 °C, KBH_4_/THF at −78 °C, and LiAl(O*t*-Bu)_3_H/THF at −78 °C. Despite these variations, the selectivity did not improve significantly. Ultimately, the best results were obtained using (*R*)-Me-CBS{(3aR)-1-Methyl-3,3-diphenyltetrahydro-1H,3H-pyrrolo[1,2-c][1,3,2]oxazaborole}/THF at −78 °C [26], achieving a remarkable selectivity of dr > 10:1 with an 89% yield. The resulting alcohol was treated with TBSOTf to afford product **20** in a high yield of 89%. Subsequent deprotection of the benzyl group at 50 °C using DDQ in a wet DCE solvent [27] yielded secondary alcohol **4** in a moderate yield of 69%. To confirm the relative stereochemistry at positions C37 and C39, the TBS protecting group in compound **4** was removed, followed by 1,3-diol acetonide protection to afford acetonide **21** in an excellent 93% yield. Analysis of the ^13^C NMR chemical shift of acetonide **21** confirmed the anticipated 1,3-syn configuration based on the distinct shifts of the ketal methyl groups (19.6 and 30.2 ppm for equatorial and axial methyl groups, respectively) [28] and the ketal carbon (97.8 ppm). Next, compound **4** was subjected to esterification using freshly prepared Fmoc-ʟ-(*N*-Me)Ala-Cl, yielding ester **22** with a 66% yield, without epimerization. An olefin cross-metathesis reaction between substrate **22** and methyl acrylate **23**, catalyzed by Grubbs II in DCM at 50 °C (sealed tube), proceeded smoothly, affording the desired aldehyde in an 81% yield. Finally, Pinnick oxidation of the resulting aldehyde using NaClO_2_ successfully delivered the unsaturated carboxylic acid **3**.

**Scheme 4:**
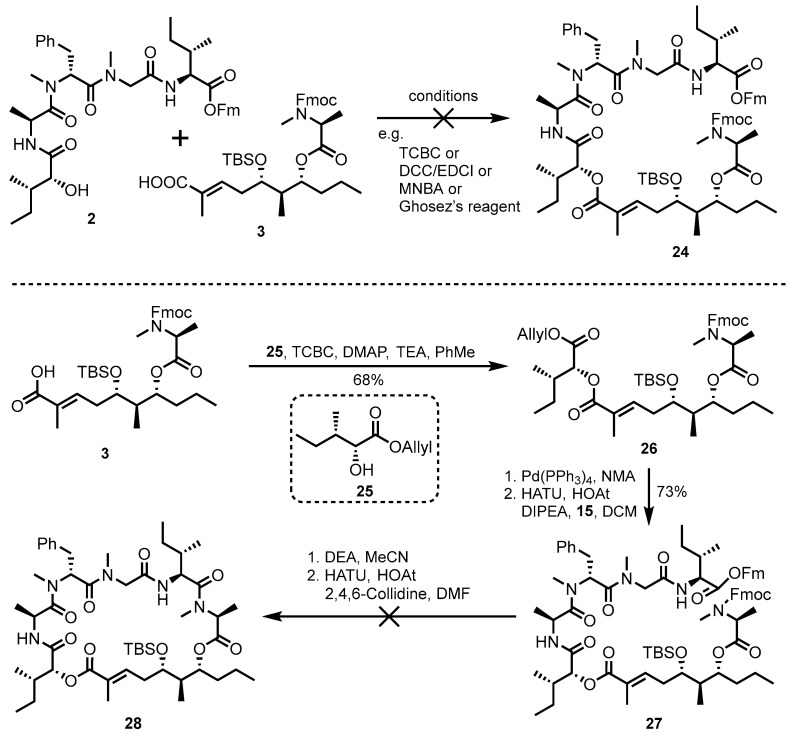
Initial attempts towards the total synthesis of lagunamide D (**1**).

With pentapeptide **2** and carboxylic acid **3** in hand, we next focused on their esterification (Scheme 4). Despite multiple attempts using Yamaguchi esterification [29], Steglich esterification [30], Shiina esterification [31], and the Ghosez reagent [32], we were unable to obtain the desired product. We speculated that this failure was due to the relatively low reactivity of conjugate acid **3** compared to a saturated acid, as well as the inherent complexity of the flexible alcohol **2**. To overcome this challenge, we shifted our focus to coupling alcohol **25** with acid **3**. Under Yamaguchi conditions, this reaction successfully yielded the key intermediate **26** in an acceptable 68% yield. Subsequent deprotection of the allyl group from compound **26**, followed by condensation with amine **15** under HATU conditions, afforded cyclization precursor **27** in a satisfactory 73% overall yield. Finally, after removing the Fm (9-Fluorenylmethyl) and Fmoc (Fluorenylmethoxycarbonyl) protecting groups using diethylamine, we investigated the macrocyclization reaction. Although HRMS confirmed the presence of the molecular ion peak corresponding to the target product, isolation of the cyclized compound proved challenging due to the low yield of this reaction.

**Scheme 5:**
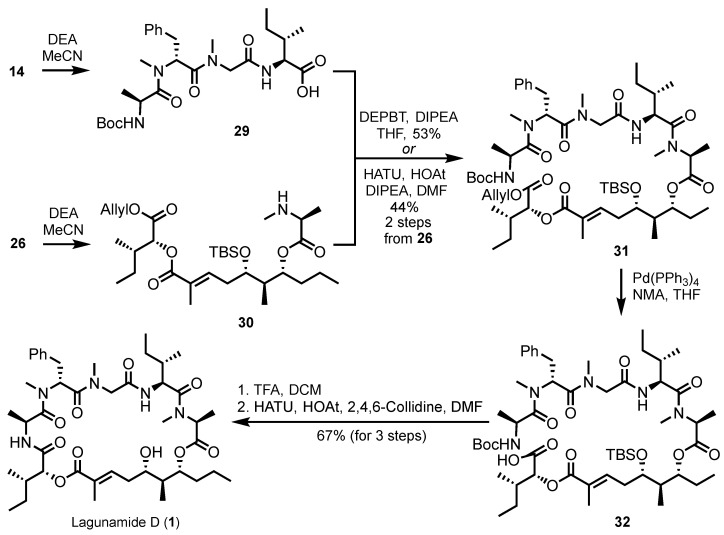
Completion of the total synthesis of lagunamide D (**1**).

To complete the total synthesis of lagunamide D, the macrocyclization site was reselected between the α-hydroxy acid and ʟ-Ala moiety (Scheme 5). To achieve this, the Fm and Fmoc protecting groups of compounds **14** and **26** were removed, yielding acid **29** and amine **30**, respectively. These intermediates were then subjected to HATU/HOAt/DIPEA/DMF conditions, forming the cyclization precursor **31** with an overall yield of 44% over two steps. Notably, using DEPBT(3-(Diethoxyphosphoryloxy)-1,2,3- benzotriazin-4(3H)-one)/DIPEA/THF conditions [33,34] improved the yield to 53%. Subsequent deprotection of the allyl group using Pd(PPh_3_)_4_/NMA(*N*-methylaniline)/THF provided carboxylic acid **32**. Treatment with TFA removed the Boc and TBS protecting groups. Finally, macrocyclization under HATU/HOAt/2,4,6-collidine/DMF conditions yielded compound **1** with an overall yield of 67% over three steps, completing the synthesis of lagunamide D. Acyl migration between lagunamides D and D′ has been reported in common HPLC solvents like methanol and acetonitrile, complicating purification. Fortunately, no such structural transformations were observed during our synthesis. In our synthetic procedure, DMF was utilized in the macrolactamization, while purification was carried out using an ethyl acetate/petroleum ether system, with the exclusion of methanol at every stage. This stability of lagunamide D during these processes may be attributed to the different conformations of the substrate in various solvents, as proposed by Kimura and Oishi in their investigations on the acyl migration of kulokekahilide-2 [35] and odoamide [36], respectively. The ^1^H and ^13^C NMR spectra of synthetic lagunamide D matched those of the natural product (see Figure S1 and Table S1 in the Supporting Information). Additionally, the optical rotation was measured at $[\alpha {]}_{D}^{20.0}$ = −57.1 (*c* 0.07, MeOH), which, while slightly higher than the reported value of $[\alpha {]}_{D}^{20.0}$ = −34.7 (*c* 0.05, MeOH) [10], confirmed the absolute stereochemistry of lagunamide D, as shown in Figure 1.

## 3. Materials and Methods

### 3.1. General Experiment

All reactions were conducted in flame-dried or oven-dried glassware under an atmosphere of dry nitrogen or argon. Oxygen- and/or moisture-sensitive solids and liquids were transferred appropriately. The concentration of solutions in vacuo was accomplished using a rotary evaporator fitted with a diaphragm vacuum pump. Residual solvents were removed under an oil vacuum pump (0.1–0.2 mm Hg). All reaction solvents were purified before use: Tetrahydrofuran (THF) was distilled from Na/benzophenone. Toluene was distilled over molten sodium metal. Dichloromethane (DCM), 1,2-dichloroethane (DCE), *N*,*N*-dimethylformamide (DMF), acetonitrile (MeCN), *N*,*N*-diisopropylethylamine (DIPEA), 2,4,6-collidine, and trimethylamine (Et_3_N) were distilled from CaH_2_. The reagents were purchased at the highest commercial quality and used without further purification unless otherwise stated. Flash column chromatography was performed using the indicated solvents on silica gel (100–200 mesh, Xinnuo, Yantai, China). Reactions were monitored using thin-layer chromatography (TLC), which was carried out using pre-coated sheets (Xinnuo silica gel coated with fluorescent indicator F254, 0.25 mm). Compounds were visualized with UV light, iodine, and ceric ammonium molybdate stainer phosphomolybdic acid in EtOH. The ^1^H NMR spectra were recorded on 500 MHz or 600 MHz spectrometers (Bruker Avance, Karlsruhe, Germany). Chemical shifts are reported in parts per million (ppm), relative to either a tetramethylsilane (TMS) internal standard or the signals of the deuterated solvent. The following abbreviations are used to describe the spin multiplicity: s = singlet, d = doublet, t = triplet, q = quartet, m = multiplet, br = broad, dd = doublet of doublets, dt = doublet of triplets, dq = doublet of quartets, ddd = doublet of doublet of doublets; other combinations are derived from those listed above. Coupling constants (J) are reported in hertz (Hz) for the corresponding solutions, and chemical shifts are reported as parts per million (ppm) relative to residual CHCl_3_ δH (7.26 ppm). ^13^C-NMR nuclear magnetic resonance spectra were recorded at 126 MHz or 151 MHz for the corresponding solutions, and chemical shifts are reported as parts per million (ppm) relative to residual CDCl_3_ δC (77.16 ppm). All high-resolution mass spectra (HRMS) were obtained by Thermo Scientific’s UltiMate 3000 Series liquid system (Germering, Bavaria, Germany) and Thermo Scientific’s Q-Exactive combined quadrupole Orbitrap mass spectrometer (Germering, Bavaria, Germany). Optical rotations were recorded on a Rudolph AutoPol-I polarimeter (Shanghai, China) at 589 nm with a 50 mm cell. Data are reported as follows: specific rotation (c (g/100 mL), solvent).

### 3.2. Synthesis of Dipeptide **10**

To a solution of Boc-*N*-Me-ᴅ-Phe-OH (1.00 g, 3.6 mmol) and *N*-Me-Gly-OMe⸱HCl (0.65 g, 4.7 mmol) in anhydrous DMF (5.0 mL), HOAt (0.10 g, 0.72 mmol), DIPEA (3.1 mL, 17.9 mmol), and HATU (2.72 g, 7.2 mmol) were added at room temperature, and the mixture was stirred overnight. The reaction mixture was quenched with saturated NH_4_Cl (aq.), and the aqueous phase was extracted with ethyl acetate (20.0 mL × 3). The combined organic layers were then washed with water and brine, dried over anhydrous Na_2_SO_4_, filtered, and concentrated. The resulting residue was purified by flash chromatography on silica gel (PE(petroleum ether)/EA(Ethyl acetate) = 2:1), yielding dipeptide **10** (1.20 g, 93%) as a colorless oil. $[\alpha {]}_{D}^{25.0}$= +110.7 (*c* 0.7, MeOH); ^1^H NMR (500 MHz, CDCl_3_) exists as rotational conformers: δ 7.31–7.11 (m, 5H), 5.33 (dd, *J* = 8.4, 6.7 Hz, 0.33H), 5.21 (dd, *J* = 9.1, 6.0 Hz, 0.17H), 5.02 (dd, *J* = 9.6, 5.1 Hz, 0.33H), 4.85 (dd, *J* = 9.2, 5.3 Hz, 0.17H), 4.39–4.14 (m, 1H), 4.04–3.90 (m, 0.67H), 3.78 (d, *J* = 17.2 Hz, 0.33H), 3.72 (s, 1H), 3.71 (s, 1H), 3.68 (s, 0.5H), 3.67 (s, 0.5H), 3.14 (dd, *J* = 13.9, 6.7 Hz, 0.5H), 3.07 (dd, *J* = 22.3, 5.7 Hz, 0.5H), 3.04–2.98 (m, 3H), 2.98–2.90 (m, 1H), 2.84 (s, 1H), 2.81 (s, 1H), 2.73 (s, 0.5H), 2.70 (s, 0.5H), 1.31 (s, 3H), 1.25 (s, 1.5H), 1.17 (s, 3H), 1.12 (s, 1.5H); ^13^C NMR (126 MHz, CDCl_3_) exists as rotational conformers: δ 170.9, 170.6, 170.5, 170.1, 169.5, 169.5, 169.4, 169.1, 155.2, 155.2, 154.3, 153.9, 138.1, 138.0, 137.6, 137.5, 129.5, 129.5, 129.5, 128.4, 128.4, 128.2, 126.4, 126.3, 80.4, 80.1, 80.1, 80.0, 58.1, 57.9, 55.8, 55.6, 52.3, 52.2, 52.1, 52.1, 50.7, 50.5, 50.0, 49.9, 36.0, 35.8, 35.1, 35.1, 29.6, 28.9, 28.9, 28.2, 28.1, 27.9, 27.7; HRMS (ESI-TOF) *m*/*z*: C_19_H_28_N_2_NaO_5_^+^ [M + Na]^+^: calcd: 387.1890; found: 387.1896.

### 3.3. Synthesis of Tripeptide **12**

Dipeptide **10** (2.97 g, 8.2 mmol) was dissolved in a mixture of THF-MeOH-H_2_O (10.0 mL/10.0 mL/5.0 mL), and LiOH (1 M in H_2_O, 16.5 mL, 16.5 mmol) was added at room temperature. The mixture was stirred for 2 h and then acidified to pH = 5 with 1 M HCl (aq.). The aqueous phase was extracted with ethyl acetate (30.0 mL × 3), and the combined organic phase was washed with brine, dried over anhydrous Na_2_SO_4_, filtered, and concentrated to yield the crude acid, which was not further purified. To a solution of the residue acid and amine **11** (3.62 g, 7.5 mmol) in anhydrous DMF (25.0 mL), HOAt (1.03 g, 7.5 mmol), DIPEA (9.2 mL, 52.7 mmol), and HATU (35.7 g, 15.1 mmol) were added at room temperature, and the mixture was stirred overnight. The reaction mixture was quenched with saturated NH_4_Cl (aq.) and extracted with ethyl acetate (30.0 mL × 3). The combined organic layers were washed with saturated NH_4_Cl (aq.) and brine, dried over anhydrous Na_2_SO_4_, filtered, and concentrated. The resulting residue was purified by flash chromatography on silica gel (PE/EA = 2:1), yielding tripeptide **12** (3.60 g, 68%) as a colorless oil. $[\alpha {]}_{D}^{20.0}$ = +35.8 (*c* 1.5, MeOH); ^1^H NMR (500 MHz, CDCl_3_) exists as rotational conformers: δ 7.85–7.74 (m, 2H), 7.66–7.55 (m, 2H), 7.46–7.38 (m, 2H), 7.38–7.31 (m, 2H), 7.30–7.14 (m, 5H), 6.82 (d, *J* = 8.1 Hz, 0.15H), 6.63 (d, *J* = 8.6 Hz, 0.3H), 6.56 (d, *J* = 8.6 Hz, 0.45H), 6.21 (d, *J* = 8.6 Hz, 0.1H), 5.37 (t, *J* = 7.6 Hz, 0.45H), 5.15 (t, *J* = 7.7 Hz, 0.15H), 5.06 (dd, *J* = 9.2, 5.4 Hz, 0.3H), 5.00 (dd, *J* = 10.4, 4.2 Hz, 0.1H), 4.64–4.51 (m, 3H), 4.28–4.17 (m, 1.5H), 4.09–3.95 (m, 0.8H), 3.95–3.80 (m, 0.3H), 3.75 (d, *J* = 15.3 Hz, 0.4H), 3.17 (dd, *J* = 14.0, 7.1 Hz, 0.45H), 3.12–3.06 (m, 0.45H), 3.07 (s, 1H), 3.06–3.02 (m, 1.55H), 3.02–2.93 (m, 1.55H), 2.89–2.81 (m, 3H), 1.88–1.75 (m, 1H), 1.42–1.32 (m, 1H), 1.35 (s, 3H), 1.31–1.26 (m, 1H), 1.25–1.13 (m, 1H), 1.20 (s, 3H), 1.11 (s, 1H), 1.03–0.91 (m, 1H), 0.88–0.75 (m, 6H); ^13^C NMR (126 MHz, CDCl_3_) exists as rotational conformers: δ 171.6, 171.5, 171.5, 171.2, 168.5, 168.3, 155.4, 154.3, 143.6, 143.4, 143.4, 141.4, 141.4, 137.9, 137.4, 129.5, 129.4, 128.5, 128.3, 128.3, 127.9, 127.2, 126.5, 126.5, 124.9, 124.8, 120.1, 80.3, 80.1, 66.7, 66.6, 57.8, 56.5, 56.5, 55.6, 53.5, 52.7, 52.5, 46.8, 37.6, 37.5, 36.4, 36.1, 35.3, 29.7, 29.1, 28.2, 28.1, 28.0, 27.7, 24.9, 24.9, 24.8, 15.3, 15.3, 11.6; HRMS (ESI-TOF) *m*/*z*: C_38_H_48_N_3_O_6_^+^ [M + H]^+^: calcd: 642.3538; found: 642.3542.

### 3.4. Synthesis of Tetrapeptide **14**

To a solution of tripeptide **12** (3.58 g, 5.6 mmol) in anhydrous CH_2_Cl_2_ (25.0 mL), TFA (5.0 mL) was added at room temperature. The mixture was stirred for 0.5 h and then concentrated under reduced pressure to yield the crude amine, which was not further purified. To a solution of the obtained amine and acid **13** in anhydrous DMF (25.0 mL), HOAt (0.68 g, 5.0 mmol), DIPEA (6.1 mL, 34.9 mmol), and HATU (3.79 g, 10.0 mmol) were added, and the mixture was stirred at room temperature overnight. The reaction mixture was quenched with saturated NH_4_Cl (aq.), and the aqueous phase was extracted with ethyl acetate (50.0 mL × 3). The combined organic layers were then washed with saturated NH_4_Cl (aq.), water, and brine, dried over anhydrous Na_2_SO_4_, filtered, and concentrated. The resulting residue was purified by flash chromatography on silica gel (PE/EA = 3:2), yielding tetrapeptide **14** (2.40 g, 61%) as a colorless oil. $[\alpha {]}_{D}^{25.0}$ = +7.7 (*c* 1.0, MeOH); ^1^H NMR (500 MHz, CDCl_3_) exists as rotational conformers: δ 7.85–7.75 (m, 2H), 7.65–7.58 (m, 2H), 7.45–7.38 (m, 2H), 7.33 (q, *J* = 7.8 Hz, 2H), 7.30–7.12 (m, 5H), 6.83 (d, *J* = 8.3 Hz, 0.3H), 6.64 (d, *J* = 8.7 Hz, 0.7H), 5.78 (dd, *J* = 9.2, 6.7 Hz, 0.7H), 5.62 (dd, *J* = 9.8, 6.2 Hz, 0.3H), 5.38 (d, *J* = 8.2 Hz, 0.7H), 5.30 (d, *J* = 7.8 Hz, 0.3H), 4.67–4.50 (m, 3H), 4.49–4.36 (m, 1H), 4.22 (t, *J* = 6.1 Hz, 1H), 4.17 (d, *J* = 17.0 Hz, 0.3H), 4.13 (d, *J* = 15.5 Hz, 0.7H), 3.87 (d, *J* = 15.5 Hz, 0.7H), 3.72 (d, *J* = 17.0 Hz, 0.3H), 3.23–3.14 (m, 1H), 3.14–3.06 (m, 1H), 3.04 (s, 3H), 3.00 (s, 3H), 1.90–1.77 (m, 1H), 1.44–1.34 (m, 9H), 1.22–1.13 (m, 1H), 1.04–0.95 (m, 1H), 0.88 (d, *J* = 6.8 Hz, 3H), 0.85–0.75 (m, 6H); ^13^C NMR (126 MHz, CDCl_3_) exists as rotational conformers: δ 173.9, 173.3, 171.7, 171.5, 171.1, 170.2, 168.2, 155.3, 143.5, 143.3, 141.4, 141.3, 136.4, 129.3, 129.2, 128.4, 127.9, 127.9, 127.2, 127.2, 126.8, 124.9, 124.8, 120.1, 79.6, 79.5, 66.7, 66.7, 56.7, 56.4, 53.9, 53.4, 52.5, 52.4, 46.8, 46.8, 46.6, 37.6, 37.6, 36.6, 35.5, 35.4, 35.2, 30.4, 29.7, 28.3, 28.3, 25.0, 24.8, 17.7, 17.5, 15.3, 15.3, 11.7, 11.6; HRMS (ESI-TOF) *m*/*z*: C_41_H_52_N_4_NaO_7_^+^ [M + Na]^+^: calcd: 735.3728; found: 735.3726.

### 3.5. Synthesis of Pentapeptide **2**

To a solution of tripeptide **14** (2.45 g, 3.4 mmol) in anhydrous CH_2_Cl_2_ (15.0 mL), TFA (5.0 mL) was added at room temperature. The mixture was stirred for 30 min and then concentrated under reduced pressure to yield the crude amine, which was not further purified. To a solution of the obtained amine and compound **16** in anhydrous DMF (15.0 mL), HOAt (0.46 g, 3.4 mmol), DIPEA (4.1 mL, 23.7 mmol), and HATU (2.57 g, 6.8 mmol) were added, and the mixture was stirred at room temperature overnight. The reaction mixture was quenched with saturated NH_4_Cl (aq.), and the aqueous phase was extracted with ethyl acetate (50.0 mL × 3). The combined organic layers were then washed with saturated NH_4_Cl (aq.), water, and brine, dried over anhydrous Na_2_SO_4_, filtered, and concentrated. The resulting residue was purified by flash chromatography on silica gel (PE/EA = 1:5), yielding tetrapeptide **2** (1.58 g, 63%) as a colorless oil. $[\alpha {]}_{D}^{25.0}$ = +12.9 (*c* 0.9, MeOH); ^1^H NMR (500 MHz, CDCl_3_) exists as rotational conformers: δ 7.86–7.72 (m, 2H), 7.65–7.54 (m, 2H), 7.47–7.38 (m, 2H), 7.37–7.30 (m, 2H), 7.30–7.05 (m, 6H), 6.87 (d, *J* = 8.5 Hz, 0.3H), 6.63 (d, *J* = 8.7 Hz, 0.7H), 5.74 (t, *J* = 7.9 Hz, 0.7H), 5.61 (t, *J* = 8.0 Hz, 0.3H), 4.82 (q, *J* = 7.0 Hz, 0.7H), 4.72 (t, *J* = 7.0 Hz, 0.3H), 4.65–4.48 (m, 3H), 4.22 (t, *J* = 6.2 Hz, 1H), 4.20–4.09 (m, 1H), 4.09–4.02 (m, 1H), 3.88–3.76 (m, 1H), 3.23–3.13 (m, 1.3H), 3.12–3.02 (m, 4H), 3.01–2.92 (m, 3.7H), 1.91–1.74 (m, 2H), 1.53–1.42 (m, 1H), 1.35–1.24 (m, 1H), 1.24–1.13 (m, 1H), 1.09–0.96 (m, 1H), 0.96–0.88 (m, 6H), 0.88–0.71 (m, 9H); ^13^C NMR (126 MHz, CDCl_3_) δ exists as rotational conformers: 173.2, 173.1, 172.8, 171.8, 171.7, 170.9, 170.2, 168.1, 168.0, 143.5, 143.3, 141.4, 141.3, 136.3, 129.3, 129.1, 128.5, 127.9, 127.9, 127.2, 127.2, 126.9, 124.9, 124.8, 120.1, 73.9, 73.9, 66.8, 56.7, 56.4, 54.0, 53.4, 52.5, 52.3, 46.7, 45.5, 45.2, 38.6, 37.8, 37.6, 36.5, 35.4, 35.1, 30.6, 30.5, 29.7, 26.2, 25.0, 24.9, 17.6, 17.4, 15.3, 12.7, 12.6, 11.9, 11.7, 11.6; HRMS (ESI-TOF) *m*/*z*: C_42_H_54_N_4_NaO_7_^+^ ccc^+^: calcd: 749.3885; found: 749.3895.

### 3.6. Synthesis of Ketone **18**

To a stirred solution of compound **17** (2.32 g, 4.4 mmol) and *N*,*O*-dimethylhydroxylamine hydrochloride (3.03 g, 31.1 mmol) in anhydrous THF (15.0 mL), *i*-PrMgCl (26.6 mL, 53.3 mmol) was added dropwise at −30 °C. After stirring for 1 h, the reaction mixture was warmed to −20 °C. After stirring for 1 h, the reaction mixture was warmed to 0 °C. After stirring for an additional hour, the reaction mixture was quenched with saturated NH_4_Cl (aq.), and the aqueous phase was extracted with ethyl acetate (50.0 mL × 3). The combined organic layers were then washed with saturated NH_4_Cl (aq.) and brine, dried over anhydrous Na_2_SO_4_, filtered, and concentrated. The resulting residue was purified by flash chromatography on silica gel (PE/EA = 4:1), yielding Weinreb amide **S1** (0.95 g, 78%) as a colorless oil. $[\alpha {]}_{D}^{25.0}$ = −8.0 (*c* 1.0, MeOH); ^1^H NMR (500 MHz, CDCl_3_) δ 7.43–7.25 (m, 5H), 4.54 (s, 2H), 3.75 (ddd, *J* = 8.7, 6.8, 3.1 Hz, 1H), 3.65 (s, 3H), 3.29–3.17 (m, 1H), 3.22 (s, 3H), 1.69–1.58 (m, 1H), 1.58–1.40 (m, 3H), 1.10 (d, *J* = 6.9 Hz, 3H), 0.95 (t, *J* = 7.1 Hz, 3H); ^13^C NMR (126 MHz, CDCl_3_) δ 176.4, 139.0, 128.2, 127.8, 127.3, 81.0, 72.8, 61.4, 39.1, 33.5, 32.0, 18.0, 14.4, 13.5; HRMS (ESI-TOF) *m*/*z*: C_16_H_25_NNaO_3_^+^ [M + Na]^+^: calcd: 302.1727; found: 302.1737.

To a stirred solution of compound **S1** (1.50 g, 5.4 mmol) in anhydrous THF (20.0 mL), allylmagnesium bromide (13.6 mL, 13.6 mmol, 1.0 M in THF) was added at 0 °C, and the mixture was stirred for 0.5 h at room temperature. The reaction mixture was quenched with saturated NH_4_Cl (aq.), and the aqueous phase was extracted with ethyl acetate (30.0 mL × 3). The combined organic layers were then washed with saturated NH_4_Cl (aq.) and brine, dried over anhydrous Na_2_SO_4_, filtered, and concentrated. The resulting residue was purified by flash chromatography on silica gel (PE/EA = 30:1), yielding ketone **18** (1.23 g, 86%) as a colorless oil. $[\alpha {]}_{D}^{25.0}$ = −57.8 (*c* 0.9, MeOH); ^1^H NMR (500 MHz, CDCl_3_) δ 7.40–7.27 (m, 5H), 5.95 (ddt, *J* = 17.1, 10.2, 6.9 Hz, 1H), 5.19 (dd, *J* = 10.2, 1.5 Hz, 1H), 5.10 (dd, *J* = 17.2, 1.6 Hz, 1H), 4.52 (d, *J* = 11.1 Hz, 1H), 4.43 (d, *J* = 11.0 Hz, 1H), 3.71 (ddd, *J* = 8.2, 6.0, 3.6 Hz, 1H), 3.28 (dd, *J* = 6.9, 1.7 Hz, 2H), 2.93 (dq, *J* = 8.3, 7.0 Hz, 1H), 1.58–1.39 (m, 4H), 1.05 (d, *J* = 7.0 Hz, 3H), 0.95 (t, *J* = 7.0 Hz, 3H); ^13^C NMR (126 MHz, CDCl_3_) δ 211.8, 138.4, 130.7, 128.3, 127.9, 127.6, 118.6, 81.0, 72.3, 49.0, 48.3, 33.1, 17.7, 14.3, 12.6; HRMS (ESI-TOF) *m*/*z*: C_17_H_24_NaO_2_^+^ [M + Na]^+^: calcd: 283.1669; found: 283.1677.

### 3.7. Synthesis of Compound **20**

To a solution of ketone **18** (0.40 g, 1.5 mmol) in anhydrous THF (20.0 mL), (*R*)-Me-CBS (2.3 mL, 2.3 mmol, 1.0 M in THF) was added under an argon atmosphere, and the mixture was stirred for 10 min at room temperature. After cooling the reaction mixture to −78 °C, BH_3_⸱Me_2_S (0.20 mL, 2.0 mmol, 10.0 M in THF) was added, and the mixture was stirred for 3 h. The reaction mixture was then warmed to −20 °C and stirred for 0.5 h. The reaction mixture was quenched with MeOH (0.10 mL) and saturated NH_4_Cl (aq.) (5.0 mL) at 0 °C, and the aqueous phase was extracted with ethyl acetate (20.0 mL × 3). The combined organic layers were then washed with saturated NH_4_Cl (aq.) and brine, dried over anhydrous Na_2_SO_4_, filtered, and concentrated. The resulting residue was purified by flash chromatography on silica gel (PE/EA = 30:1), yielding alcohol **S2** (0.40 g, 89%) as a colorless oil. $[\alpha {]}_{D}^{25.0}$ = +4.1 (*c* 1.0, MeOH); ^1^H NMR (500 MHz, CDCl_3_) δ 7.42–7.28 (m, 5H), 6.00–5.86 (m, 1H), 5.25–5.12 (m, 2H), 4.62–4.46 (m, 2H), 3.68–3.57 (m, 2H), 2.49–2.38 (m, 1H), 2.22–2.08 (m, 1H), 2.00–1.89 (m, 1H), 1.68–1.57 (m, 1H), 1.57–1.49 (m, 2H), 1.48–1.40 (m, 1H), 0.95 (t, *J* = 7.2 Hz, 3H), 0.88 (d, *J* = 7.0 Hz, 3H); ^13^C NMR (126 MHz, CDCl_3_) δ 138.5, 135.2, 128.4, 127.9, 127.6, 117.8, 82.2, 73.17, 7.35, 4.55, 39.2, 32.3 18.0, 14.4, 11.9; HRMS (ESI-TOF) *m*/*z*: C_17_H_26_NaO_2_^+^ [M + Na]^+^: calcd: 285.1825; found: 285.1832.

To a solution of alcohol **S2** (2.22 g, 8.5 mmol) in anhydrous CH_2_Cl_2_ (20.0 mL), Et_3_N (4.7 mL, 33.8 mmol) and TBSOTf (3.9 mL, 16.9 mmol) were added at room temperature under an argon atmosphere, and the mixture was stirred for 1 h. The reaction mixture was quenched with saturated NH_4_Cl (aq.), and the aqueous phase was extracted with ethyl acetate (30.0 mL × 3). The combined organic layers were then washed with saturated NH_4_Cl (aq.) and brine, dried over anhydrous Na_2_SO_4_, filtered, and concentrated under reduced pressure. The resulting residue was purified by flash chromatography on silica gel (PE/EA = 30:1), yielding compound **20** (2.80 g, 89%) as a colorless oil. $[\alpha {]}_{D}^{25.0}$ = +4.5 (*c* 0.9, MeOH); ^1^H NMR (500 MHz, CDCl_3_) δ 7.45–7.24 (m, 5H), 5.95–5.83 (m, 1H), 5.11–5.01 (m, 2H), 4.56–4.44 (m, 2H), 3.82 (td, *J* = 6.1, 4.5 Hz, 1H), 3.53 (ddd, *J* = 8.6, 5.7, 3.2 Hz, 1H), 2.39–2.26 (m, 1H), 2.26–2.18 (m, 1H), 2.11–1.99 (m, 1H), 1.63–1.54 (m, 1H), 1.54–1.42 (m, 2H), 1.42–1.28 (m, 1H), 0.94 (t, *J* = 6.9 Hz, 3H), 0.92 (s, 9H), 0.86 (d, *J* = 7.0 Hz, 3H), 0.08 (s, 3H), 0.05 (s, 3H); ^13^C NMR (126 MHz, CDCl_3_) δ 139.2, 135.4, 128.3, 127.8, 127.3, 116.7, 79.8, 72.8, 71.0, 40.2, 38.1, 32.3, 25.9, 18.9, 18.1, 14.5, 10.1, −4.0, −4.7; HRMS (ESI-TOF) *m*/*z*: C_23_H_40_NaO_2_Si^+^ [M + Na]^+^: calcd: 399.2690; found: 399.2699.

### 3.8. Synthesis of Alcohol **4**

To a solution of compound **20** (1.62 g, 4.3 mmol) in wet DCE (25.0 mL, analytically pure), 2,3-dichloro-5,6-dicyano-1,4-benzoquinone (3.89 g, 17.2 mmol) was added at 50 °C, and the mixture was stirred for 1 h. The reaction mixture was cooled to room temperature and quenched with saturated Na_2_S_2_O_3_ (aq.) (20.0 mL), after which the aqueous phase was extracted with DCM (50.0 mL × 3). The combined organic layers were washed with 1 M NaOH (aq.)-saturated Na_2_S_2_O_3_ (aq.) (V*_NaOH_*: V*_Na2S2O3_* = 1:1) and brine, dried over anhydrous Na_2_SO_4_, filtered, and concentrated under reduced pressure. The resulting residue was purified by flash chromatography on silica gel (PE/EA = 20:1), yielding alcohol **4** (0.85 g, 69%) as a colorless oil. $[\alpha {]}_{D}^{25.0}$ = +3.3 (*c* 1.2, MeOH); ^1^H NMR (500 MHz, CDCl_3_) δ 5.94–5.82 (m, 1H), 5.18–4.99 (m, 2H), 3.80 (q, *J* = 5.4 Hz, 1H), 3.61–3.54 (m, 1H), 2.74 (s, 1H), 2.41–2.33 (m, 1H), 2.32–2.23 (m, 1H), 1.76–1.68 (m, 1H), 1.58–1.49 (m, 2H), 1.43–1.33 (m, 2H), 0.95 (t, *J* = 6.6 Hz, 3H), 0.93 (s, 9H), 0.84 (d, *J* = 7.0 Hz, 3H), 0.11 (s, 6H); ^13^C NMR (126 MHz, CDCl_3_) δ 135.0, 117.1, 75.9, 73.8, 43.1, 39.3, 36.5, 25.9, 18.6, 18.0, 14.2, 12.8, −4.2, −4.7; HRMS (ESI-TOF) *m*/*z*: C_16_H_34_NaO_2_Si^+^ [M + Na]^+^: calcd: 309.2220; found: 309.2226.

### 3.9. Synthesis of Acetonide **21**

To a solution of compound **4** (53.0 mg, 0.18 mmol) in anhydrous THF (3.0 mL), TBAF (0.4 mL, 0.4 mmol) was added at room temperature under an argon atmosphere, and the mixture was stirred for 30 min. The reaction mixture was concentrated under reduced pressure. The resulting residue was purified by flash chromatography on silica gel (PE/EA = 1:1), yielding diol **S4** as a colorless oil.

To a solution of the diol **S4** obtained above in anhydrous CH_2_Cl_2_ (1.0 mL), 2,2-dimethoxypropane (3.0 mL, 25.1 mmol) and PTSA (2.0 mg, 11.6 mmol) were added at room temperature under an argon atmosphere, and the mixture was stirred for 5 min. The reaction mixture was quenched with saturated Et_3_N (0.5 mL, 3.6 mmol), and the solvent was concentrated under reduced pressure. The resulting residue was purified by flash chromatography on silica gel (PE/EA = 50:1), yielding acetonide **21** (36.7 mg, 93%) as a colorless oil. $[\alpha {]}_{D}^{20.0}$ = +14.7 (*c* 0.8, MeOH); ^1^H NMR (500 MHz, CDCl_3_) δ 5.93 (ddt, *J* = 17.2, 10.3, 6.8 Hz, 1H), 5.11–5.02 (m, 2H), 3.54 (ddd, *J* = 10.2, 7.0, 3.1 Hz, 1H), 3.46 (ddd, *J* = 10.3, 8.0, 2.4 Hz, 1H), 2.39 (dddd, *J* = 12.8, 6.5, 3.2, 1.6 Hz, 1H), 2.20 (dt, *J* = 14.6, 7.1 Hz, 1H), 1.60–1.48 (m, 2H), 1.43 (s, 3H), 1.39 (s, 3H), 1.37–1.29 (m, 3H), 0.91 (t, *J* = 7.0 Hz, 3H), 0.79 (d, *J* = 6.6 Hz, 3H); ^13^C NMR (126 MHz, CDCl_3_) δ 135.2, 116.2, 97.8, 74.1, 73.7, 37.7, 37.5, 35.2, 30.2, 19.6, 18.2, 14.0, 12.2.

### 3.10. Synthesis of Ester **22**

To a solution of Fmoc-*N*-Me-ʟ-Ala-OH (3.17 g, 9.8 mmol) in anhydrous CH_2_Cl_2_ (15.0 mL) at 0 °C, oxalyl chloride (2.8 mL, 32.5 mmol) and catalytic DMF (32 μL, 0.41 mmol) were added, and the mixture was warmed to room temperature. The reaction mixture was stirred for 1 h and then concentrated under reduced pressure to yield the crude acyl chloride, which was used directly in the subsequent step. To a solution of alcohol **4** (932 mg, 3.3 mmol) and DMAP (0.99 g, 8.1 mmol) in anhydrous CH_2_Cl_2_ (15.0 mL), Et_3_N (4.5 mL, 32.5 mmol) was added at room temperature under an argon atmosphere. The reaction mixture was cooled to 0 °C, and a solution of the crude acyl chloride obtained above in anhydrous CH_2_Cl_2_ (15.0 mL) was added. The mixture was then warmed to room temperature and stirred for 1 h. The reaction was quenched with methanol (1.0 mL) and saturated NH_4_Cl (aq.) (5.0 mL), and the aqueous phase was extracted with ethyl acetate (30.0 mL × 3). The combined organic layers were washed with 1 M HCl (aq.), saturated NH_4_Cl (aq.), and brine and then dried over anhydrous Na_2_SO_4_, filtered, and concentrated. The resulting residue was purified by flash chromatography on silica gel (PE/EA = 20:1), yielding ester **22** (1.26 g, 66%) as a colorless oil. $[\alpha {]}_{D}^{20.0}$ = −6.0 (*c* 0.7, MeOH); ^1^H NMR (500 MHz, CDCl_3_) exists as rotational conformers: δ 7.85–7.77 (m, 2H), 7.70–7.57 (m, 2H), 7.46–7.37 (m, 2H), 7.37–7.30 (m, 2H), 5.91–5.78 (m, 1H), 5.20–4.97 (m, 3H), 4.97–4.91 (m, 0.7H), 4.85–4.79 (m, 0.3H), 4.49–4.35 (m, 2H), 4.33–4.23 (m, 1H), 3.72–3.63 (m, 1H), 2.97–2.91 (m, 3H), 2.34–2.24 (m, 1H), 2.22–2.13 (m, 1H), 1.99–1.90 (m, 1H), 1.64–1.48 (m, 2H), 1.45 (d, *J* = 7.3 Hz, 3H), 1.40–1.20 (m, 2H), 1.00–0.82 (m, 15H), 0.12–0.01 (m, 6H); ^13^C NMR (126 MHz, CDCl_3_) exists as rotational conformers: δ 171.5, 171.3, 156.5, 155.9, 144.1, 144.9, 141.3, 135.4, 135.0, 127.7, 127.1, 125.1, 125.1, 120.0, 119.8, 116.9, 76.1, 76.0, 72.9, 67.9, 67.8, 54.2, 47.3, 41.6, 41.5, 37.9, 37.7, 32.8, 32.5, 30.4, 30.2, 25.9, 18.8, 18.7, 18.1, 15.4, 14.9, 14.1, 10.4, −4.1, −4.7; HRMS (ESI-TOF) *m*/*z*: C_35_H_51_NNaO_5_Si^+^ [M + Na]^+^: calcd: 616.3429; found: 616.3436.

### 3.11. Synthesis of Ester **26**

In a sealed tube, α-methylacrolein (11.1 mL, 3.5 mmol) and Grubbs II catalyst (8.9 mg, 10.0 μmol) were added to a solution of ester **22** (1.22 g, 2.1 mmol) in anhydrous CH_2_Cl_2_ (10.0 mL) at room temperature. After purging with nitrogen gas for 30 min, the reaction mixture was warmed to 50 °C and stirred overnight. The mixture was then concentrated under reduced pressure. The resulting residue was purified by flash chromatography on silica gel (PE/EA = 10:1), yielding aldehyde **S3** (1.06 g, 81%) as a colorless oil. $[\alpha {]}_{D}^{25.0}$ = −21.3 (*c* 0.8, MeOH); ^1^H NMR (500 MHz, CDCl_3_) exists as rotational conformers: δ 9.38 (s, 0.73H), 9.35 (s, 0.27H), 7.87–7.74 (m, 2H), 7.68–7.55 (m, 2H), 7.52–7.41 (m, 2H), 7.41–7.30 (m, 2H), 6.67–6.48 (m, 1H), 5.14–5.00 (m, 1H), 4.94–4.81 (m, 1H), 4.52–4.33 (m, 2H), 4.33–4.20 (m, 1H), 3.95–3.79 (m, 1H), 2.96 (s, 2.2H), 2.92 (s, 0.8H), 2.59–2.42 (m, 2H), 2.02–1.93 (m, 1H), 1.74 (s, 3H), 1.65–1.51 (m, 2H), 1.49–1.42 (m, 3H), 1.41–1.22 (m, 2H), 0.99–0.84 (m, 15H), 0.13–−0.02 (m, 6H); ^13^C NMR (126 MHz, CDCl_3_) δ exists as rotational conformers: 195.3, 195.0, 171.9, 156.5, 152.0, 151.0, 144.0, 143.9, 141.3, 140.3, 127.7, 127.1, 127.1, 125.0, 120.0, 120.0, 76.0, 75.8, 71.6, 71.4, 68.0, 67.8, 54.2, 47.2, 42.6, 42.3, 33.4, 32.2, 30.2, 25.8, 18.6, 18.5, 18.0, 15.3, 14.9, 14.2, 14.1, 10.3, 10.2, 9.4. −4.4, −4.7; HRMS (ESI-TOF) *m*/*z*: C_37_H_53_NNaO_6_Si^+^ [M + Na]^+^: calcd: 658.3534; found: 658.3541.

To a stirred solution of aldehyde **S3** (1.05 g, 1.7 mmol) and 2-methyl-2-butene (16 mL) in *t*-BuOH/pH = 7 phosphate buffer (2:1, 24.0 mL), NaClO_2_ (4.33 g, 49.5 mmol) and NaH_2_PO_4_ (9.53 g, 61.1 mmol) were added at room temperature, and the mixture was stirred for 1 h. The reaction mixture was acidified to pH = 3 with 1 M HCl (aq.), and the aqueous phase was then extracted with ethyl acetate (30.0 mL × 3). The combined organic layers were washed with saturated NH_4_Cl (aq.) and brine, dried over anhydrous Na_2_SO_4_, filtered, and concentrated. The resulting residue was purified by flash chromatography on silica gel (PE/EA = 1:1), yielding acid **3** (1.05 g, 98%) as a colorless oil.

To a solution of acid **3** (353 mg, 0.58 mmol), alcohol **25** (300 mg, 1.70 mmol), and DMAP (0.35 g, 2.90 mmol) in anhydrous PhMe (10.0 mL) at −20 °C, 2,4,6-trichlorobenzoyl chloride (0.30 mL, 1.70 mmol) was added, and the mixture was stirred for 0.5 h. The reaction mixture was then warmed to room temperature and stirred overnight. The mixture was acidified to pH = 3 with 1 M HCl (aq.), and the organic phase was extracted with ethyl acetate (30.0 mL × 3). The combined organic layers were washed with 1 M HCl (aq.), saturated NaHCO_3_ (aq.), and brine, dried over anhydrous Na_2_SO_4_, filtered, and concentrated. The resulting residue was purified by flash chromatography on silica gel (PE/EA = 1:3), yielding ester **26** (318 mg, 68%) as a colorless oil. $[\alpha {]}_{D}^{25.0}$ = −25.0 (c 0.8, MeOH); ^1^H NMR (600 MHz, CDCl_3_) exists as rotational conformers: δ 7.84–7.76 (m, 2H), 7.72–7.56 (m, 2H), 7.48–7.39 (m, 2H), 7.39–7.31 (m, 2H), 7.00–6.88 (m, 1H), 5.91 (ddt, J = 16.5, 10.9, 5.6 Hz, 1H), 5.33 (d, J = 17.4 Hz, 1H), 5.24 (d, J = 10.3 Hz, 1H), 5.08 (d, J = 3.5 Hz, 1H), 5.07–5.03 (m, 1H), 4.95–4.86 (m, 0.66H), 4.83–4.79 (m, 0.34H), 4.75–4.60 (m, 2H), 4.49–4.36 (m, 2H), 4.32–4.23 (m, 1H), 3.86–3.79 (m, 1H), 2.93 (s, 2H), 2.91 (s, 1H), 2.42–2.30 (m, 2H), 2.10–2.00 (m, 1H), 2.00–1.91 (m, 1H), 1.88 (s, 2.5H), 1.85 (s, 0.5H), 1.63–1.51 (m, 3H), 1.51–1.39 (m, 4H), 1.37–1.23 (m, 2H), 0.99 (d, J = 6.9 Hz, 3H), 0.98–0.81 (m, 18H), 0.10–0.00 (m, 6H);^13^C NMR (151 MHz, CDCl_3_) exists as rotational conformers: δ 171.6, 171.2, 169.9, 167.4, 156.4, 155.8, 144.1, 143.9, 141.3, 141.3, 140.6, 140.2, 131.8, 127.7, 127.1, 125.1, 125.1, 120.0, 118.5, 76.2, 76.0, 74.8, 71.7, 71.6, 67.9, 67.8, 65.5, 54.3, 54.1, 47.2, 42.5, 42.4, 36.8, 33.4, 33.1, 32.4, 32.3, 30.2, 29.7, 26.1, 25.8, 18.5, 17.9, 15.3, 14.9, 14.5, 14.1, 12.7, 11.7, 10.4. −4.3, −4.7; HRMS (ESI-TOF) m/z: C_46_H_67_NNaO_9_Si^+^ [M + Na]^+^: calcd: 828.4477; found: 828.4487.

### 3.12. Synthesis of Compound **27**

To a solution of compound **26** (0.14 g, 0.17 mmol) and Pd(PPh_3_)_4_ (19.6 mg, 17 μmol) in anhydrous THF (2.0 mL), PhNHMe (37.7 μL, 0.34 mmol) was added. The resulting mixture was stirred at room temperature for 1 h. The reaction was quenched with TFA (75.6 μL, 1.02 mmol), diluted with ethyl acetate (30.0 mL), and washed sequentially with 1 M HCl (aq.) (3.0 mL × 2). The organic phase was washed with brine, dried over anhydrous Na_2_SO_4_, filtered, and concentrated to afford the crude acid, which was used in the next step without further purification. The crude acid was dissolved in anhydrous DMF (5.0 mL); HOAt (22.74 mg, 0.17 mmol), DIPEA (0.29 mL, 1.7 mmol), amine **15** (0.11 g, 0.15 mmol), and HATU (0.12 g, 0.34 mmol) were added at room temperature; and the mixture was stirred overnight. The reaction mixture was quenched with saturated NH_4_Cl (aq.), and the aqueous phase was extracted with ethyl acetate (20.0 mL × 3). The combined organic layers were then washed with water and brine, dried over anhydrous Na_2_SO_4_, filtered, and concentrated. The resulting residue was purified by flash chromatography on silica gel (PE/EA = 1:1), yielding compound **27** (0.17 g, 73%) as a colorless oil. $[\alpha {]}_{D}^{20.0}$ = −12.9 (*c* 0.6, MeOH); ^1^H NMR (600 MHz, Chloroform-*d*) exists as rotational conformers: δ 7.83–7.74 (m, 4H), 7.66–7.53 (m, 4H), 7.49–7.38 (m, 4H), 7.37–7.30 (m, 4H), 7.27–7.15 (m, 5H), 6.95–6.89 (m, 1H), 6.65 (d, *J* = 7.4 Hz, 1H), 6.52 (d, *J* = 8.6 Hz, 1H), 5.84 (dd, *J* = 9.5, 6.5 Hz, 0.7H), 5.76 (t, *J* = 8.2 Hz, 0.3H), 5.23–5.18 (m, 1H), 5.07–4.98 (m, 1H), 4.93–4.87 (m, 1H), 4.75–4.68 (m, 1H), 4.59–4.51 (m, 3H), 4.45–4.37 (m, 2H), 4.30–4.20 (m, 2H), 4.06–3.94 (m, 2H), 3.90–3.82 (m, 1H), 3.22–3.12 (m, 2H), 3.12–2.89 (m, 9H), 2.83 (s, 1H), 2.39–2.33 (m, 2H), 2.00–1.93 (m, 2H), 1.90–1.87 (m, 3H), 1.85–1.78 (m, 1H), 1.61–1.52 (m, 2H), 1.48–1.21 (m, 7H), 1.21–0.55 (m, 31H), 0.11–−0.02 (m, 6H); ^13^C NMR (151 MHz, CDCl_3_) exists as rotational conformers: 172.4, 171.7, 171.6, 170.8, 170.8, 169.5, 168.1, 166.7, 156.4, 144.1, 143.9, 143.6, 143.4, 141.4, 141.3, 136.5, 129.3, 128.3, 127.9, 127.9, 127.7, 127.2, 127.1, 124.8, 120.1, 76.1, 76.1, 75.9, 71.7, 67.8, 66.7, 56.6, 56.4, 56.3, 54.3, 54.1, 53.8, 53.7, 52.7, 52.2, 47.2, 46.8, 45.0, 42.6, 38.6, 37.8, 37.7, 37.6, 37.5, 36.7, 36.5, 35.2, 35.1, 33.4, 32.5, 31.9, 30.6, 30.4, 30.2, 29.7, 29.7, 29.4, 26.1, 25.8, 24.9, 18.5, 18.0, 17.3, 17.2, 15.3, 14.9, 14.1, 14.1, 13.9, 12.8, 11.7, 11.6, 11.5, 10.4, −4.2, −4.7. HRMS (ESI-TOF) *m*/*z*: C_79_H_105_N_5_NaO_13_Si^+^ [M + Na]^+^: calcd: 1382.7370; found: 1382.7390.

### 3.13. Synthesis of Compound **31**

To a solution of compound **14** (28 mg, 36 μmol) in anhydrous CH_3_CN (2.0 mL), diethylamine (Et_2_NH) (1.0 mL) was added, and the mixture was stirred for 2 h at room temperature. The reaction mixture was then concentrated under reduced pressure, and the residue was dissolved in ethyl acetate (50.0 mL) and washed with 1 M HCl (aq.) (3.0 mL × 2) and brine. The organic phase was then dried over anhydrous Na_2_SO_4_, filtered, and concentrated to afford crude **29**, which was used in the next step without further purification. To a solution of compound **26** (16 mg, 20 μmol) in anhydrous CH_3_CN (2.0 mL), diethylamine (Et_2_NH) (1.0 mL) was added, and the mixture was stirred for 2 h at room temperature. The mixture was then concentrated under reduced pressure to afford crude **30**, which was used in the next step without further purification. The above crude acid **29** and amine **30** were dissolved in anhydrous THF (3.0 mL), and DEPBT (13 mg, 45 μmol) and DIPEA (7.8 μL, 45 μmol) were added successively at room temperature. The mixture was then stirred overnight. The reaction mixture was quenched with saturated NH_4_Cl (5.0 mL), and the organic phase was extracted with ethyl acetate (30.0 mL × 3). The combined organic layers were washed with brine, dried over anhydrous Na_2_SO_4_, filtered, and concentrated. The resulting residue was purified by flash chromatography on silica gel (PE/EA = 2:3), yielding compound **31** (11.5 mg, 53%) as a colorless oil. $[\alpha {]}_{D}^{20.0}$ = −16.4 (*c* 0.5, MeOH); ^1^H NMR (600 MHz, CDCl_3_) exists as rotational conformers: δ 7.39–7.13 (m, 5H), 7.13–7.03 (m, 1H), 7.02–6.91 (m, 1H), 6.81–6.66 (m, 1H), 5.98–5.87 (m, 1H), 5.78–5.68 (m, 1H), 5.67–5.59 (m, 1H), 5.38–5.30 (m, 1H), 5.30–5.22 (m, 1H), 5.13–5.07 (m, 1H), 5.07–4.96 (m, 1H), 4.96–4.82 (m, 1H), 4.71–4.61 (m, 2H), 4.59–4.36 (m, 2H), 4.34–4.26 (m, 1H), 3.94–3.84 (m, 1H), 3.84–3.72 (m, 1H), 3.32–3.11 (m, 2H), 3.11–2.80 (m, 8H), 2.42–2.27 (m, 2H), 2.10–2.01 (m, 1H), 1.97–1.80 (m, 4H), 1.79–1.38 (m, 18H), 1.38–1.06 (m, 15H), 1.04–0.82 (m, 18H), 0.09–−0.02 (m, 6H); ^13^C NMR (151 MHz, CDCl_3_) exists as rotational conformers: δ 173.1, 172.0, 171.3, 170.9, 170.7, 169.8, 167.9, 167.5, 155.3, 140.8, 136.5, 131.8, 129.9, 129.4, 128.4, 128.3, 126.8, 118.6, 79.4, 76.1, 74.9, 71.8, 65.5, 54.2, 53.2, 52.2, 51.9, 46.6, 42.8, 37.6, 36.8, 36.4, 35.3, 33.4, 32.4, 31.9, 31.3, 30.4, 29.7, 29.7, 29.4, 28.3, 28.3, 26.1, 25.8, 24.0, 22.7, 18.7, 18.0, 17.7, 15.5, 14.5, 14.5, 14.1, 14.1, 12.6, 11.7, 11.2, 10.4, −4.3, −4.7, −4.7; HRMS (ESI-TOF) *m*/*z*: C_58_H_97_N_5_NaO_13_Si^+^ [M + Na]^+^: calcd: 1122.6744; found: 1122.6726.

### 3.14. Synthesis of Lagunamide D (1)

To a solution of compound **31** (26 mg, 24 μmol) and Pd(PPh_3_)_4_ (2.7 mg, 2.4 μmol) in anhydrous THF (2.0 mL), PhNHMe (5.2 μL, 48 μmol) was added. The resulting mixture was stirred at room temperature for 1 h. The reaction was quenched with TFA (10 μL, 144 μmol), diluted with ethyl acetate (30.0 mL), and washed sequentially with 1 M HCl (aq.) (3.0 mL × 2). The organic phase was washed with brine, dried over anhydrous Na_2_SO_4_, filtered, and concentrated to afford the crude acid **32**, which was used in the next step without further purification. To a solution of the crude acid in anhydrous CH_2_Cl_2_ (2.5 mL), TFA (0.50 mL) was added, and the mixture was stirred at room temperature for 2 h. The mixture was concentrated under reduced pressure, the residue was dissolved in DMF (12.0 mL), and HOAt (16 mg, 0.12 mmol), 2,4,6-collidine (94 μL, 0.72 mmol), and HATU (90 mg, 0.24 mmol) were added successively. The mixture was stirred for 48 h and then concentrated under reduced pressure. The residue was dissolved in ethyl acetate and washed with 1 M HCl (aq.), water, and brine. The organic phase was then dried over anhydrous Na_2_SO_4_, filtered, and concentrated. The resulting residue was purified by flash chromatography on silica gel (PE/EA = 2:3 to pure EA), yielding lagunamide D (13 mg, 67%) as a colorless foam. $[\alpha {]}_{D}^{20.0}$ = −57.1 (*c* 0.07, MeOH); ^1^H NMR (600 MHz, DMSO-*d*_6_) exists as rotational conformers: δ 8.49 (d, *J* = 6.4 Hz, 0.3H), 8.34 (d, *J* = 8.3 Hz, 0.1H), 7.23–7.05 (m, 5.1H), 6.92–6.74 (m, 0.2H), 6.74–6.54 (m, 0.2H), 5.81–5.67 (m, 0.2H), 5.28 (dd, *J* = 10.2, 5.3 Hz, 0.6H), 5.23–4.99 (m, 0.6H), 4.92 (d, *J* = 7.6 Hz, 0.6H), 4.90–4.81 (m, 1.0H), 4.81–4.72 (m, 0.7H), 4.72–4.44 (m, 0.9H), 4.31 (dq, *J* = 6.3, 6.3 Hz, 0.6H), 4.25–4.07 (m, 0.8H), 3.98 (d, *J* = −18.4 Hz, 0.5H), 3.92 (q, *J* = 6.6 Hz, 0.6H), 3.87–3.72 (m, 0.3H), 3.72–3.38 (m, 0.7H), 3.21 (s, 2.4H), 3.16–3.06 (m, 0.6H), 3.06–2.88 (m, 1.9H), 2.85 (s, 2.7H), 2.80–2.76 (m, 0.8H), 2.73 (s, 2.2H), 2.64 (s, 1.2H), 2.45–2.18 (m, 1.2H), 2.10 (ddd, *J* = −14.0, 9.8, 9.8 Hz, 0.9H), 1.95–1.85 (m, 1.2H), 1.85–1.64 (m, 4.9H), 1.62–1.08 (m, 11.0H), 1.06–0.41 (m, 21.0H); ^13^C NMR (151 MHz, DMSO-*d*_6_) δ 172.45, 170.80, 170.14, 170.11, 169.52, 168.23, 168.01, 144.45, 137.17, 129.18, 127.48, 126.83, 125.84, 75.36, 74.62, 69.30, 58.07, 52.45, 51.1, 50.40, 44.65, 40.89, 37.29, 37.10, 36.27, 35.79, 34.42, 33.86, 29.60, 29.36, 25.83, 23.19, 17.05, 14.89, 14.69, 14.06, 13.92, 12.93, 11.98, 11.40, 10.27, 9.60; HRMS (ESI-TOF) *m*/*z*: C_44_H_69_N_5_NaO_10_^+^ [M + Na]^+^: calcd: 850.4937; found: 850.4909.

## 4. Conclusions

In summary, we have accomplished the total synthesis of lagunamide D and confirmed its absolute stereochemical configuration. Key features of the synthesis include Ghosh’s TiCl_4_-promoted anti-aldol reaction to establish the C38 and C39 chiral centers, a CBS reduction to construct the C37 chiral hydroxyl group, and a cross-metathesis reaction followed by a Pinnick oxidation to build the unsaturated carboxylic acid moiety. This synthetic strategy was completed in a 14-step longest linear sequence, starting from the known intermediate **17**, with an overall yield of 4.6%, which demonstrated an efficient route for access to the structurally unique 26-membered cytotoxic cyclic depsipeptide.

## Data Availability

Data are contained within the article or Supplementary Materials.

## Supplementary Materials

The following supporting information can be downloaded at https://www.mdpi.com/article/10.3390/md23030099/s1: Figure S1: Comparison of ^1^H NMR (600 MHz) for Natural and Synthetic Lagunamide D in DMSO-*d6*; Table S1: Comparison of ^13^C NMR (600 MHz) for Natural and Synthetic Lagunamide D in DMSO-*d6*.; NMR and spectra of compounds **1**, **2**, **4**, **10**, **12**, **14**, **18**, **20**–**22**, **26**, **27**, **31** and **S1**–**S3**.
