# Supplementary material for: Total Synthesis of the Marine Cyclic Depsipeptide Lagunamide D
Source: Mar Drugs. 2025 Feb 24;23(3):99. doi: 10.3390/md23030099 (PMC11944260; doi:10.3390/md23030099)
Supplement: Supplementary file 1 [file marinedrugs-23-00099-s001.zip › marinedrugs-3480754-supplementary.pdf]

Supporting Information

Total Synthesis of the Marine Cyclic Depsipeptide Lagunamide D

Huiru Nan[1], Xiong-En Long[1], Jianfei He[1], Hailiang Xing[1], Min-Jing Cheng[4], Jin-Bao Peng[1], Tao Ye[2,3,*] , Jia-Lei Yan[1,*] and Junyang Liu[1,2,*]

1 School of Pharmacy and Food Engineering, Wuyi University, Jiangmen 529020, China; huiru_nan@foxmail.com (H.N.); wananlxe@163.com (X.-E.L.); 18230816752@163.com (J.H.); 18255186096@163.com (H.X.); pengjb_05@126.com (J.-B.P.)

2 State Key Laboratory of Chemical Oncogenomics, Key Laboratory of Chemical Genomics, Peking University Shenzhen Graduate School, Shenzhen, 518055, China

3 Qian Yan (Shenzhen) Pharmatech. Ltd., Shenzhen 518172, China

4 Center for Bioactive Natural Molecules and Innovative Drugs, and Guangdong Province Key Laboratory of Pharmacodynamic Constituents of TCM and New Drugs Research, College of Pharmacy, Jinan University, Guangzhou 510632, China; chengmj1235@jnu.edu.cn

* Correspondence: yet@pkusz.edu.cn (T.Y.); yanjialei@wyu.edu.cn (J.-L.Y.); liujy@wyu.edu.cn (J.L.)

Table of Contents

1. Table S1. Comparison of ^{13}C NMR (600 MHz) for Natural and Synthetic Lagunamide D in DMSO-*d6*. S1

2. NMR Spectra S3

3. Figure S1. Comparison of ^{1}H NMR (600 MHz) for Natural and Synthetic lagunamide D in DMSO-*d6* S37

1. **Table S1**. Comparison of ^{13}C NMR (600 MHz) for Natural and Synthetic Lagunamide D in DMSO-*d6*.

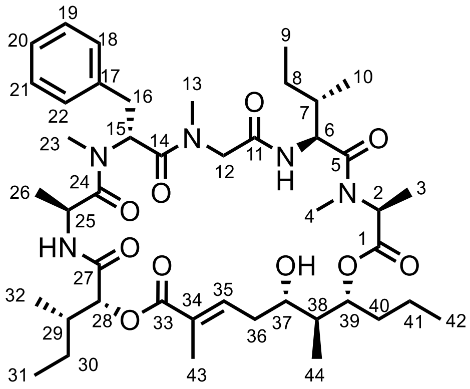

Lagunamide D (**1**)

C No.	δC*i* (Natural)/ppm	δC*s* (Synthetic)/ppm	ΔδC (δC*s*-δC*i*)/ppm
**24**	172.45	172.45	0.00
**5**	170.88	170.80	-0.08
**14**	170.18	170.14	-0.04
**1**	170.17	170.11	-0.06
**27**	169.49	169.52	0.03
**33**	168.34	168.23	-0.11
**11**	168.18	168.01	-0.17
**35**	144.45	144.45	0.00
**17**	137.16	137.17	0.01
**18/22**	129.3	129.18	-0.12
**19/21**	127.51	127.48	-0.03
**34**	126.83	126.83	0.00
**20**	125.93	125.84	-0.09
**28**	75.30	75.36	0.06
**39**	74.56	74.62	0.06
**37**	69.24	69.30	0.06
**2**	57.93	58.07	0.14
**15**	52.33	52.45	0.12
**6**	51.07	51.10	0.03
**12a**	50.38	50.4	0.02
**25**	44.51	44.65	0.14
**38**	40.85	40.89	0.04
**7**	37.20	37.29	0.09
**4**	36.95	37.10	0.15
**29**	36.21	36.27	0.06
**13**	35.65	35.79	0.14
**16a**	34.25	34.42	0.17
**40a**	33.62	33.86	0.24
**36a**	29.56	29.6	0.04
**23**	29.24	29.36	0.12

**30a**	25.65	25.83	0.18
**8a**	23.00	23.19	0.19
**41**	16.84	17.05	0.21
**26**	14.68	14.89	0.21
**10**	14.55	14.69	0.14
**42**	14.12	14.06	-0.06
**32**	14.00	13.92	-0.08
**3**	12.74	12.93	0.19
**43**	11.84	11.98	0.14
**31**	11.52	11.40	-0.12
**9**	10.13	10.27	0.14
**44**	9.40	9.60	0.20

2. NMR Spectra

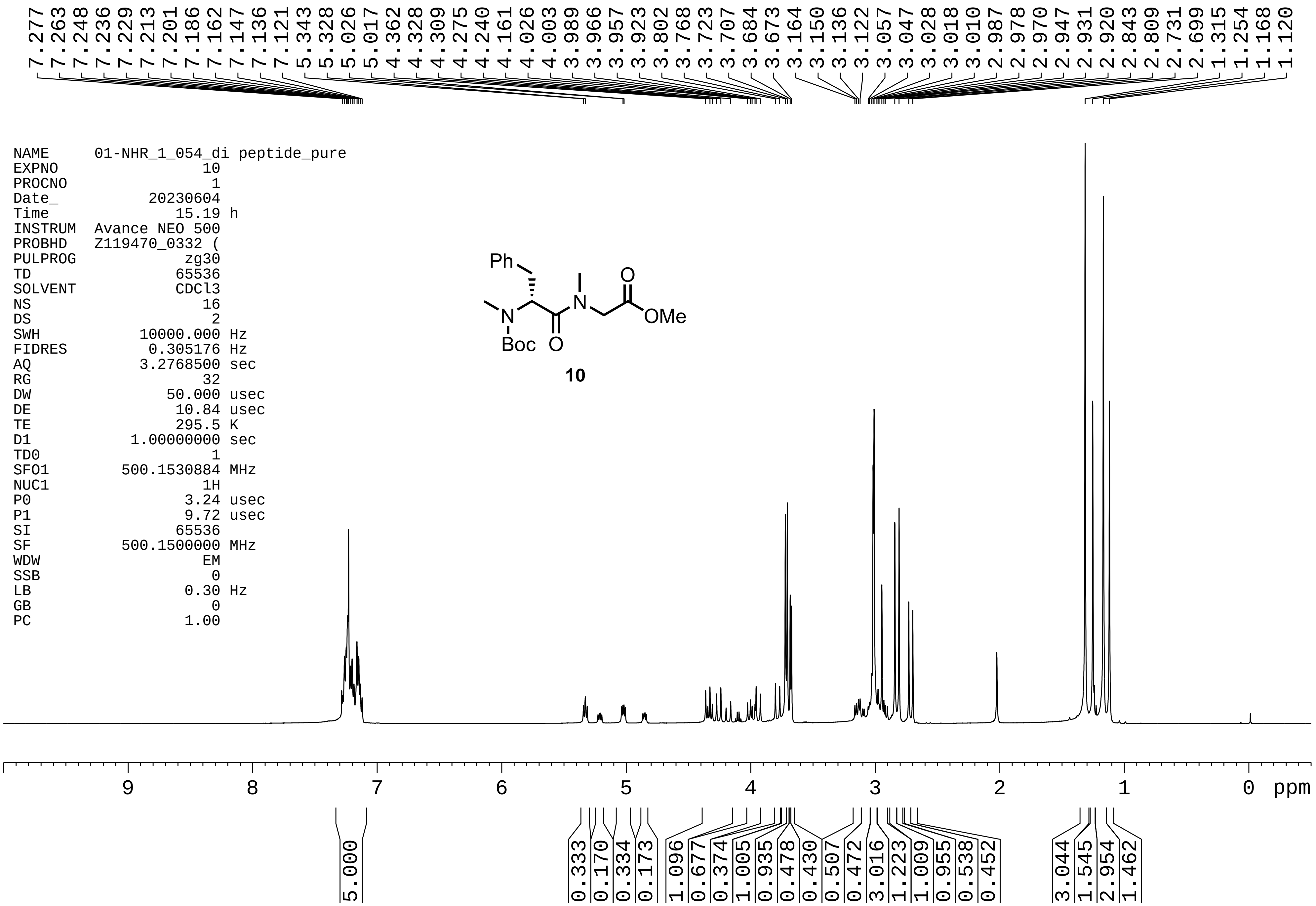

NAME	01-NHR_1_054_di peptide_pure
EXPNO	11
PROCNO	1
Date_	20230604
Time	15.31 h
INSTRUM	Avance NEO 500
PROBHD	Z119470_0332 (
PULPROG	zgpg30
TD	65536
SOLVENT	CDCl3
NS	200
DS	4
SWH	30120.482 Hz
FIDRES	0.919204 Hz
AQ	1.0879476 sec
RG	101
DW	16.600 usec
DE	6.50 usec
TE	296.2 K
D1	2.00000000 sec
D11	0.03000000 sec
TD0	1
SFO1	125.7753938 MHz
NUC1	13C
P0	3.33 usec
P1	10.00 usec
SI	32768
SF	125.7628175 MHz
WDW	EM
SSB	0
LB	1.00 Hz
GB	0
PC	1.40

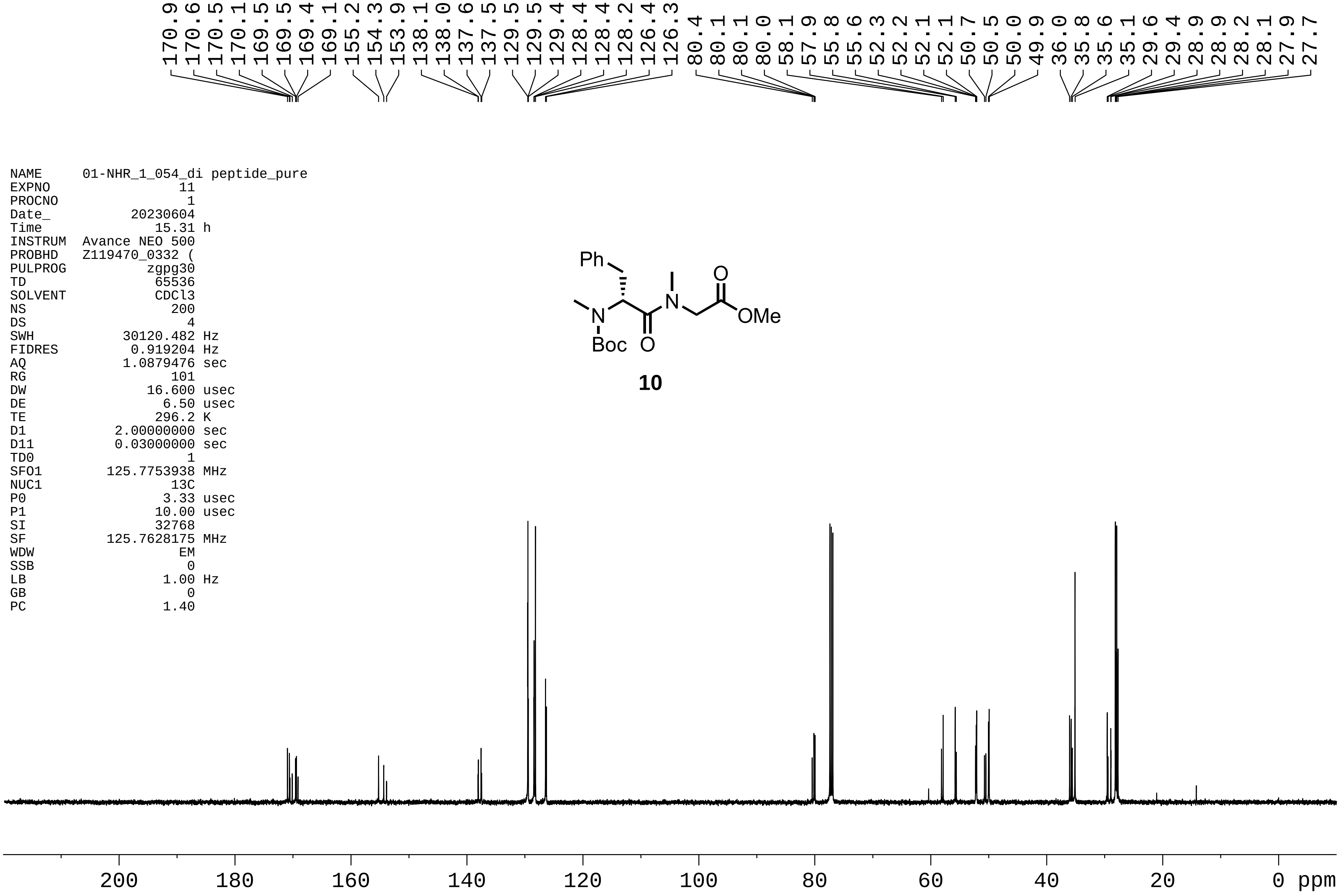

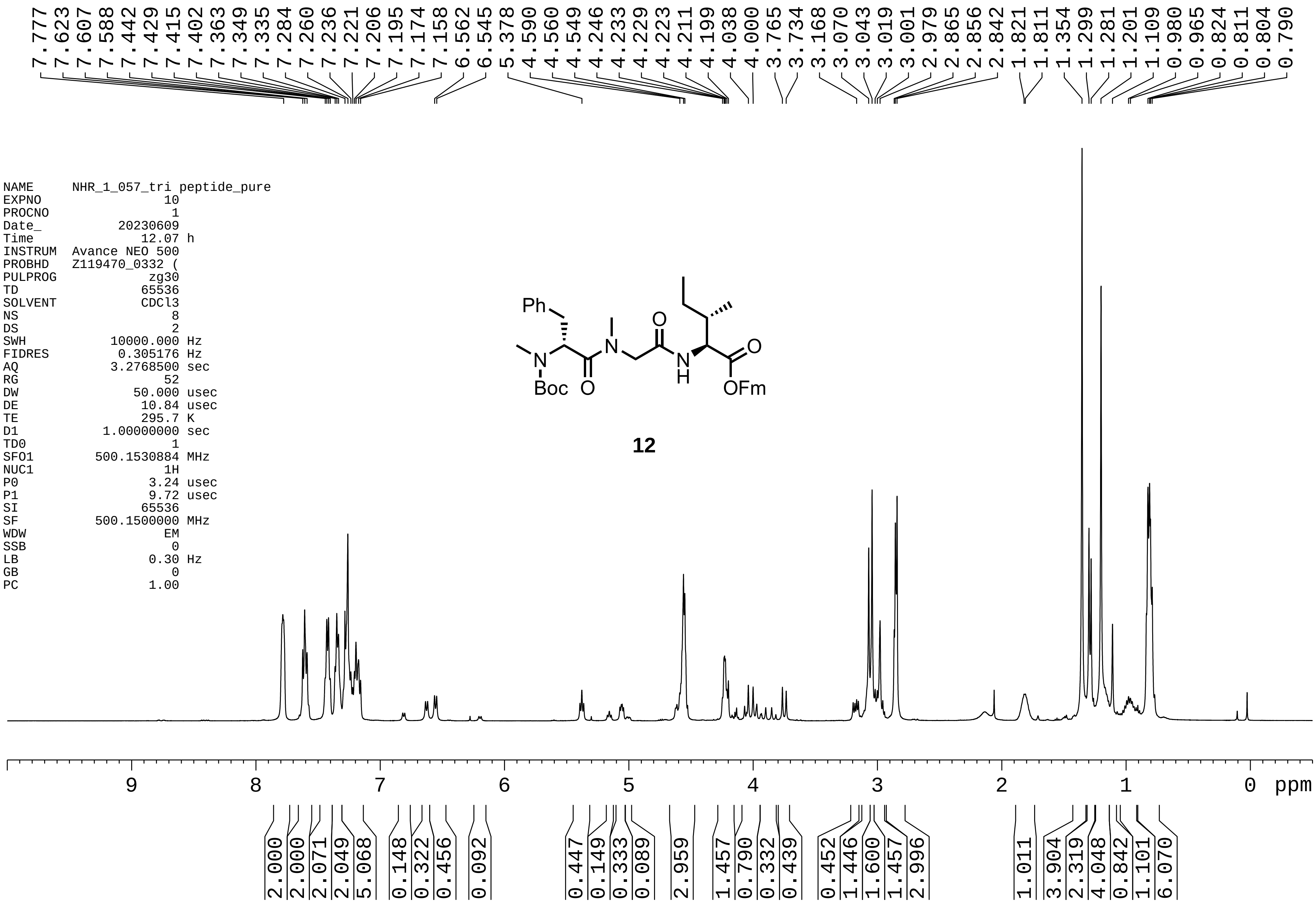
NAME NHR_1_057_tri peptide_pure
EXPNO 10
PROCNO 1
Date_ 20230609
Time 12.07 h
INSTRUM Avance NEO 500
PROBHD Z119470_0332 (
PULPROG zg30
TD 65536
SOLVENT CDCl3
NS 8
DS 2
SWH 10000.000 Hz
FIDRES 0.305176 Hz
AQ 3.2768500 sec
RG 52
DW 50.000 usec
DE 10.84 usec
TE 295.7 K
D1 1.00000000 sec
TD0 1
SFO1 500.1530884 MHz
NUC1 1H
P0 3.24 usec
P1 9.72 usec
SI 65536
SF 500.1500000 MHz
WDW EM
SSB 0
LB 0.30 Hz
GB 0
PC 1.00
Ph
N
Boc
O
N
O
N
H
O
OFm
12
7.777 7.623 7.607 7.588 7.442 7.429 7.415 7.402 7.363 7.349 7.335 7.284 7.260 7.236 7.221 7.206 7.195 7.174 7.158 6.562 6.545 5.378 4.590 4.560 4.549 4.246 4.233 4.229 4.223 4.211 4.199 4.038 4.000 3.765 3.734 3.168 3.070 3.043 3.019 3.001 2.979 2.865 2.856 2.842 1.821 1.811 1.354 1.299 1.281 1.201 1.109 0.980 0.965 0.824 0.811 0.804 0.790
9 8 7 6 5 4 3 2 1 0 ppm
2.000 2.000 2.071 2.049 5.068 0.148 0.322 0.456 0.092 0.447 0.149 0.333 0.089 2.959 1.457 0.790 0.332 0.439 0.452 1.446 1.600 1.457 2.996 1.011 3.904 2.319 4.048 0.842 1.101 6.070

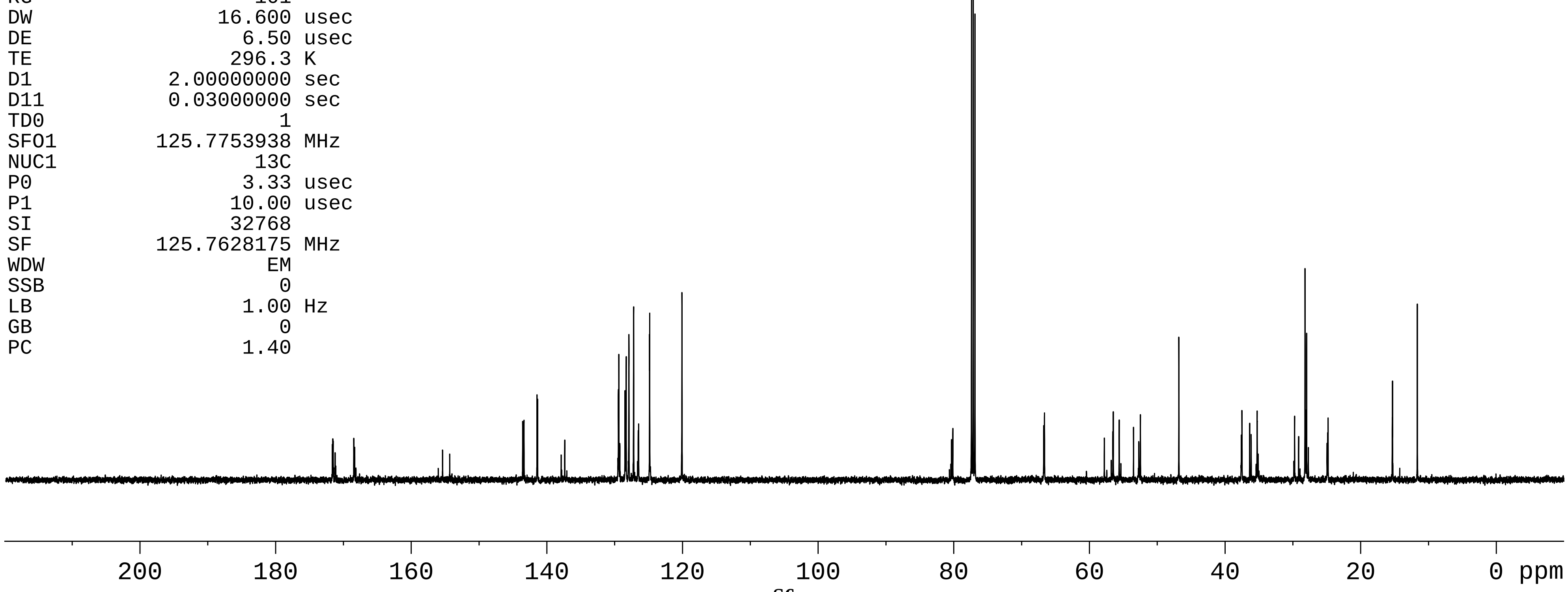

NAME	02-NHR_1_057_tri peptide	
EXPNO	11	
PROCNO	1	
Date_	20230608	
Time	10.54	h
INSTRUM	Avance NEO 500	
PROBHD	Z119470_0332 (	
PULPROG	zgpg30	
TD	65536	
SOLVENT	CDCl3	
NS	188	
DS	4	
SWH	30120.482	Hz
FIDRES	0.919204	Hz
AQ	1.0879476	sec
RG	101	
DW	16.600	usec
DE	6.50	usec
TE	296.3	K
D1	2.00000000	sec
D11	0.03000000	sec
TD0	1	
SFO1	125.7753938	MHz
NUC1	13C	
P0	3.33	usec
P1	10.00	usec
SI	32768	
SF	125.7628175	MHz
WDW	EM	
SSB	0	
LB	1.00	Hz
GB	0	
PC	1.40	

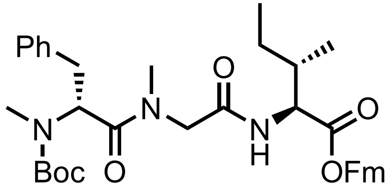

12

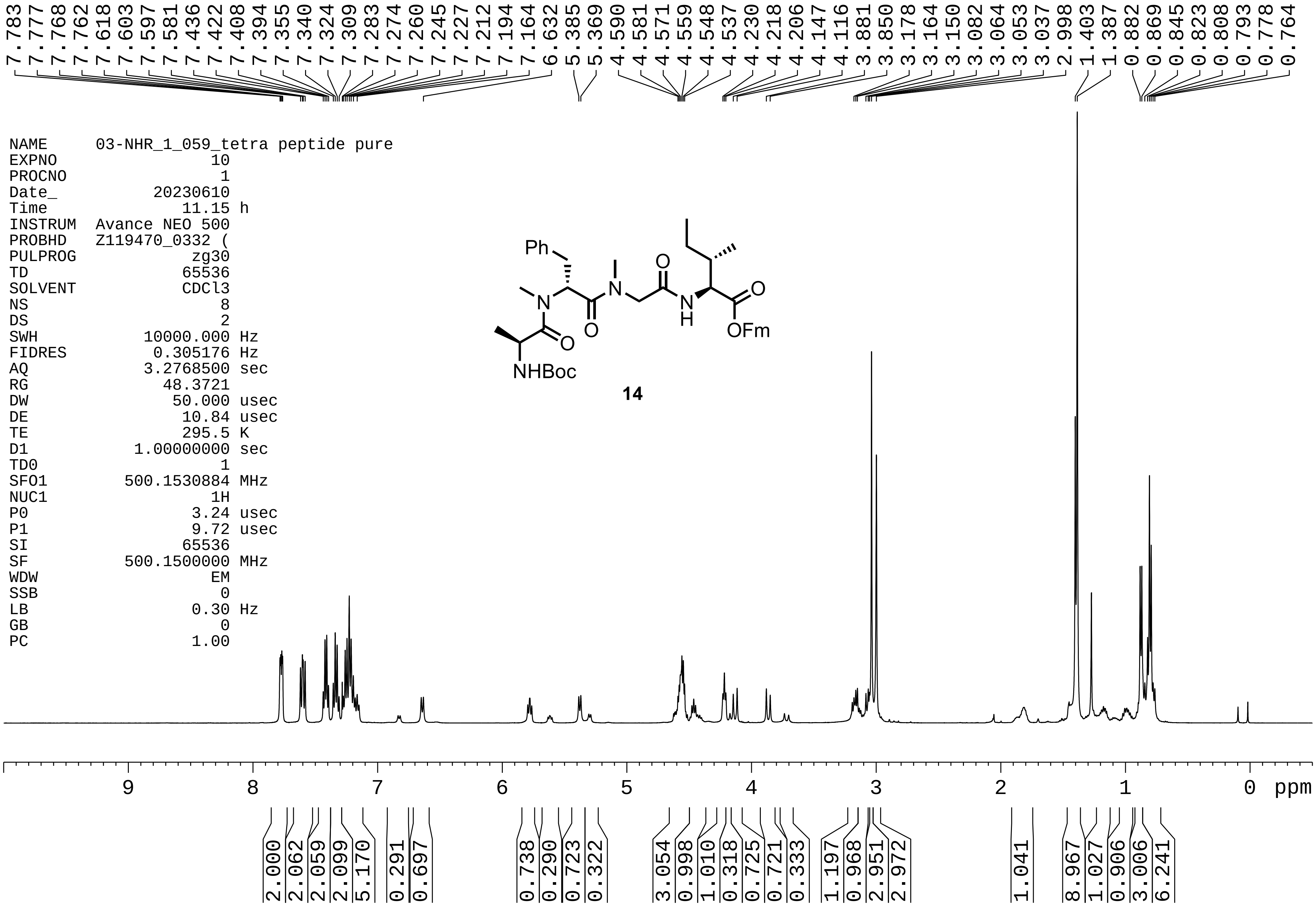
7.783 7.777 7.768 7.762 7.618 7.603 7.597 7.581 7.436 7.422 7.408 7.394 7.355 7.340 7.324 7.309 7.283 7.274 7.260 7.245 7.227 7.212 7.194 7.164 6.632 5.385 5.369 4.590 4.581 4.571 4.559 4.548 4.537 4.230 4.218 4.206 4.147 4.116 3.881 3.850 3.178 3.164 3.150 3.082 3.064 3.053 3.037 2.998 1.403 1.387 0.882 0.869 0.845 0.823 0.808 0.793 0.778 0.764
NAME 03-NHR_1_059_tetra peptide pure
EXPNO 10
PROCNO 1
Date_ 20230610
Time 11.15 h
INSTRUM Avance NEO 500
PROBHD Z119470_0332 (
PULPROG zg30
TD 65536
SOLVENT CDCl3
NS 8
DS 2
SWH 10000.000 Hz
FIDRES 0.305176 Hz
AQ 3.2768500 sec
RG 48.3721
DW 50.000 usec
DE 10.84 usec
TE 295.5 K
D1 1.00000000 sec
TD0 1
SFO1 500.1530884 MHz
NUC1 1H
P0 3.24 usec
P1 9.72 usec
SI 65536
SF 500.1500000 MHz
WDW EM
SSB 0
LB 0.30 Hz
GB 0
PC 1.00
Ph
N
O
NH
H
OFm
NHBoc
14
9 8 7 6 5 4 3 2 1 0 ppm
2.000 2.062 2.059 2.099 5.170 0.291 0.697 0.738 0.290 0.723 0.322 3.054 0.998 1.010 0.318 0.725 0.721 0.333 1.197 0.968 2.951 2.972 1.041 8.967 1.027 0.906 3.006 6.241

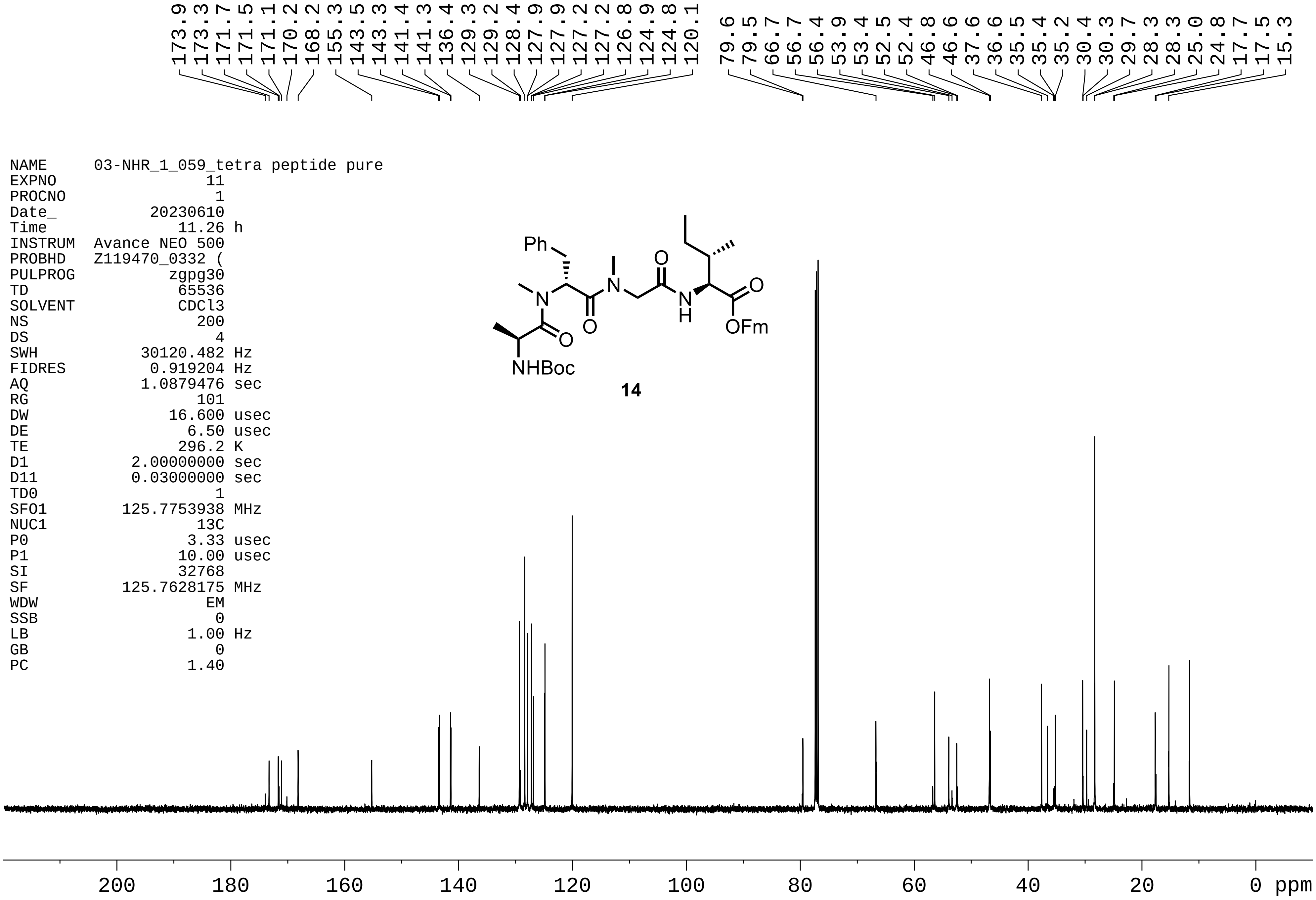
173.9
173.3
171.7
171.5
171.1
170.2
168.2
155.3
143.5
143.3
141.4
141.3
136.4
129.3
129.2
128.4
127.9
127.9
127.2
127.2
126.8
124.9
124.8
120.1
79.6
79.5
66.7
56.7
56.4
53.9
53.4
52.5
52.4
46.8
46.6
37.6
36.6
35.5
35.4
35.2
30.4
30.3
29.7
28.3
28.3
25.0
24.8
17.7
17.5
15.3
NAME 03-NHR_1_059_tetra peptide pure
EXPNO 11
PROCNO 1
Date_ 20230610
Time 11.26 h
INSTRUM Avance NEO 500
PROBHD Z119470_0332 (
PULPROG zgpg30
TD 65536
SOLVENT CDCl3
NS 200
DS 4
SWH 30120.482 Hz
FIDRES 0.919204 Hz
AQ 1.0879476 sec
RG 101
DW 16.600 usec
DE 6.50 usec
TE 296.2 K
D1 2.00000000 sec
D11 0.03000000 sec
TD0 1
SFO1 125.7753938 MHz
NUC1 13C
P0 3.33 usec
P1 10.00 usec
SI 32768
SF 125.7628175 MHz
WDW EM
SSB 0
LB 1.00 Hz
GB 0
PC 1.40
Ph
O
N
N
N
H
O
O
O
OFm
NHBoc
14
200
180
160
140
120
100
80
60
40
20
0 ppm

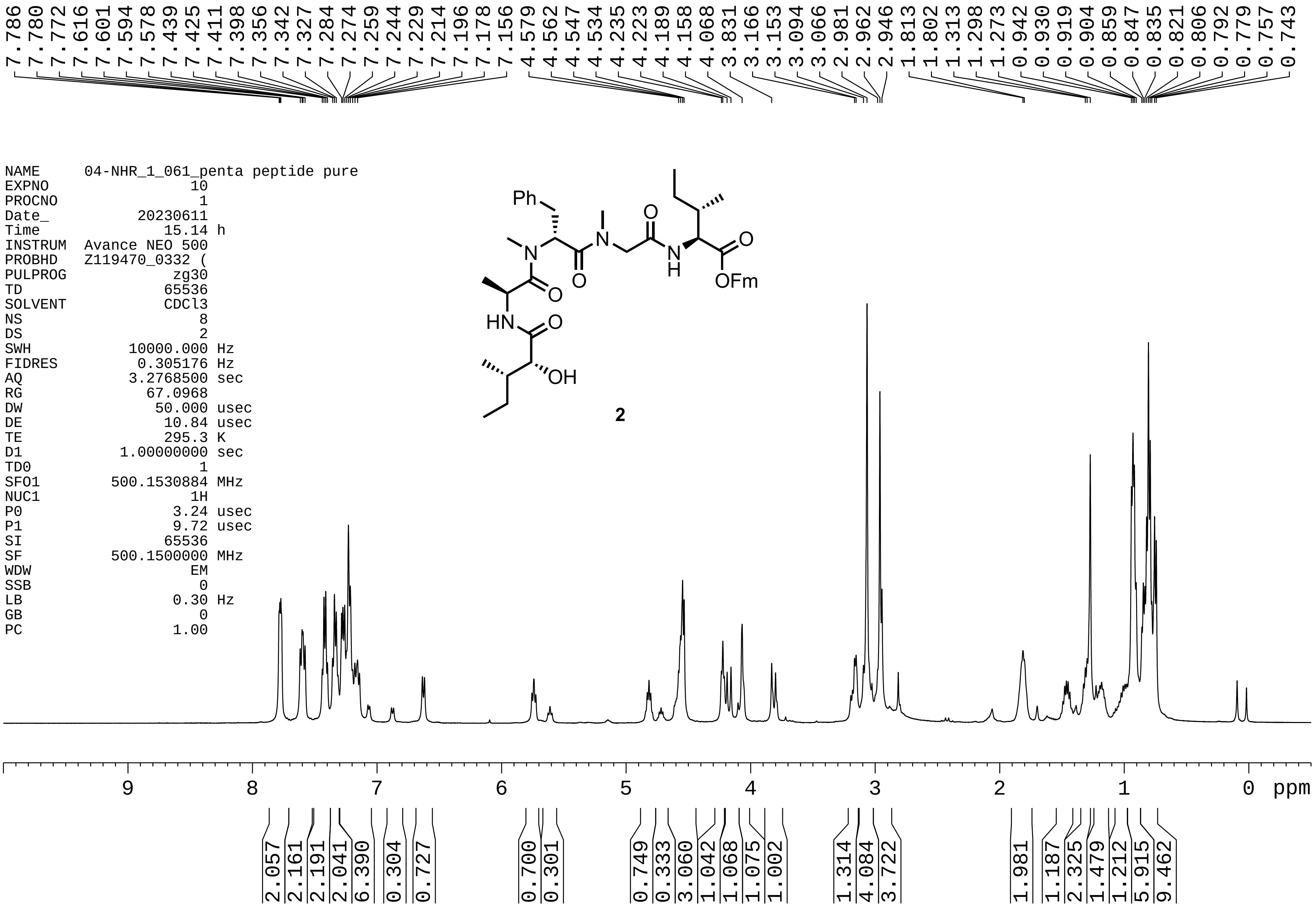
7.786 7.780 7.772 7.616 7.601 7.594 7.578 7.439 7.425 7.411 7.398 7.356 7.342 7.327 7.284 7.274 7.259 7.244 7.229 7.214 7.196 7.178 7.156 4.579 4.562 4.547 4.534 4.235 4.223 4.189 4.158 4.068 3.831 3.166 3.153 3.094 3.066 2.981 2.962 2.946 1.813 1.802 1.313 1.298 1.273 0.942 0.930 0.919 0.904 0.859 0.847 0.835 0.821 0.806 0.792 0.779 0.757 0.743
NAME 04-NHR_1_061_penta peptide pure
EXPNO 10
PROCNO 1
Date_ 20230611
Time 15.14 h
INSTRUM Avance NEO 500
PROBHD Z119470_0332 (
PULPROG zg30
TD 65536
SOLVENT CDCl3
NS 8
DS 2
SWH 10000.000 Hz
FIDRES 0.305176 Hz
AQ 3.2768500 sec
RG 67.0968
DW 50.000 usec
DE 10.84 usec
TE 295.3 K
D1 1.00000000 sec
TD0 1
SFO1 500.1530884 MHz
NUC1 1H
P0 3.24 usec
P1 9.72 usec
SI 65536
SF 500.1500000 MHz
WDW EM
SSB 0
LB 0.30 Hz
GB 0
PC 1.00
Ph
N
O
N
H
O
OFm
HN
O
OH
2
9 8 7 6 5 4 3 2 1 0 ppm
2.057 2.161 2.191 2.041 6.390 0.304 0.727 0.700 0.301 0.749 0.333 3.060 1.042 1.068 1.075 1.002 1.314 4.084 3.722 1.981 1.187 2.325 1.479 1.212 5.915 9.462

NAME 04-NHR_1_061_penta peptide pure
EXPNO 11
PROCNO 1
Date_ 20230611
Time 15.26 h
INSTRUM Avance NEO 500
PROBHD Z119470_0332 (
PULPROG zgpg30
TD 65536
SOLVENT CDCl3
NS 200
DS 4
SWH 30120.482 Hz
FIDRES 0.919204 Hz
AQ 1.0879476 sec
RG 101
DW 16.600 usec
DE 6.50 usec
TE 296.1 K
D1 2.00000000 sec
D11 0.03000000 sec
TD0 1
SFO1 125.7753938 MHz
NUC1 13C
P0 3.33 usec
P1 10.00 usec
SI 32768
SF 125.7628175 MHz
WDW EM
SSB 0
LB 1.00 Hz
GB 0
PC 1.40

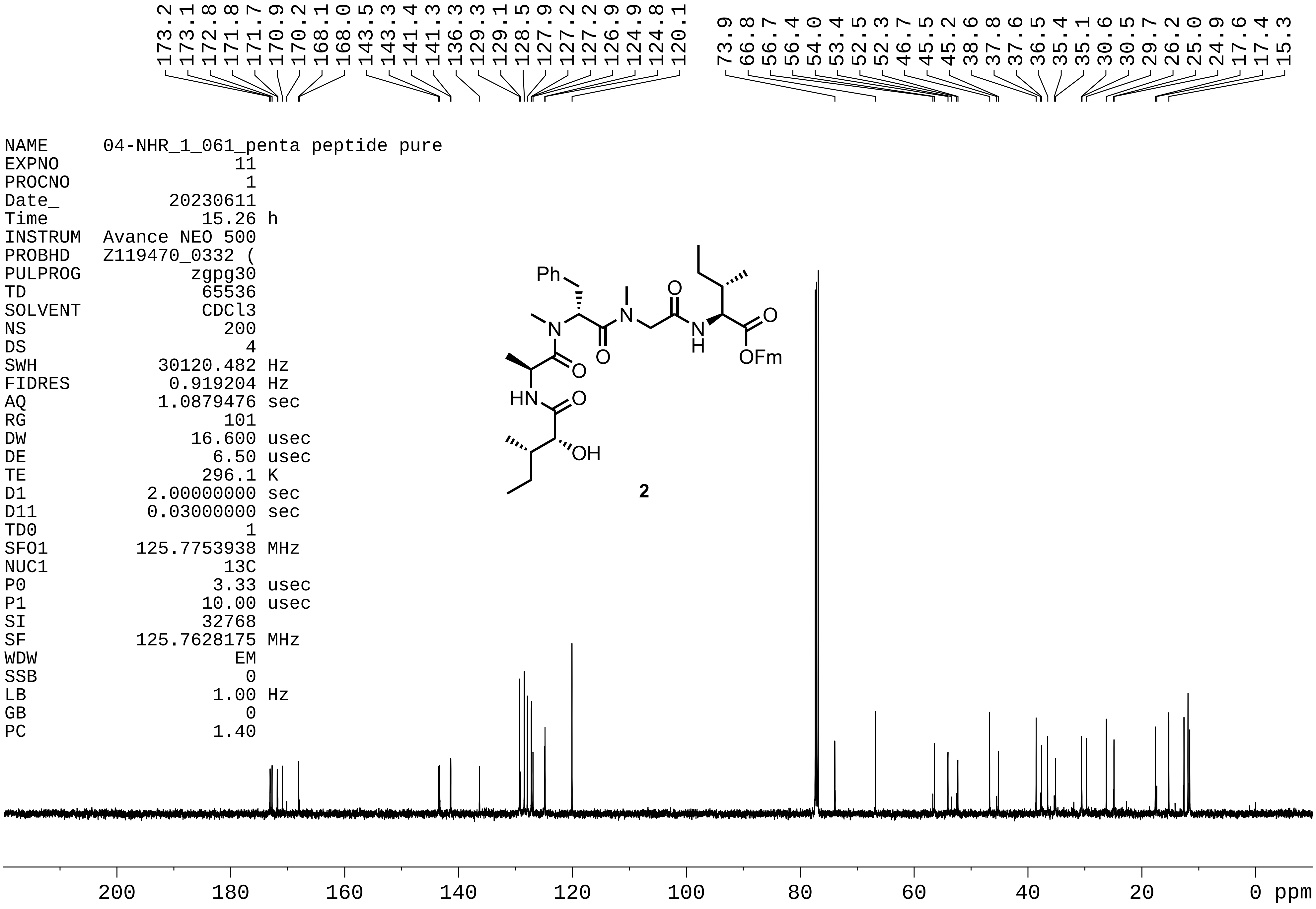

2

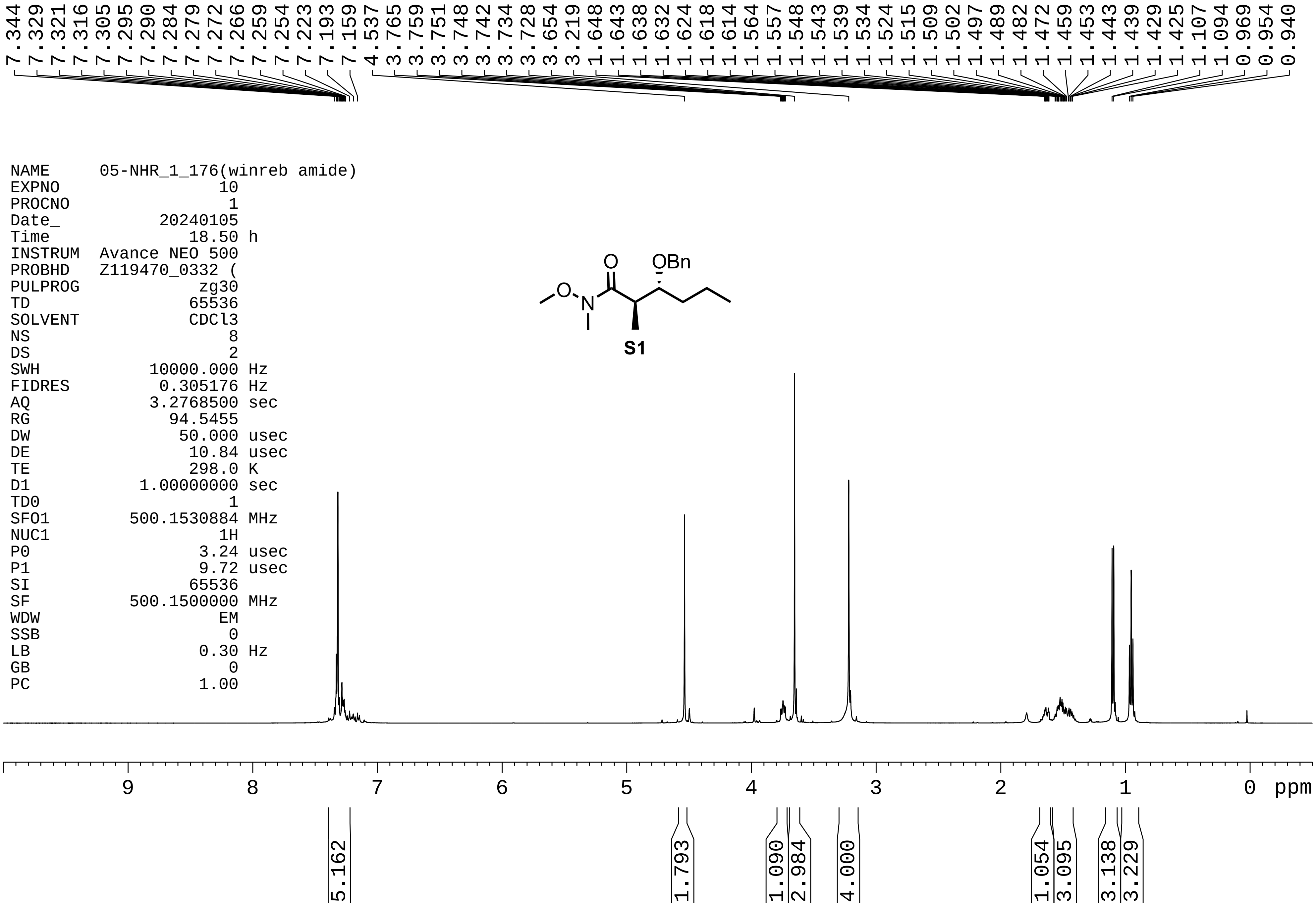
7.344 7.329 7.321 7.316 7.305 7.295 7.290 7.284 7.279 7.272 7.266 7.259 7.254 7.223 7.193 7.159 4.537 3.765 3.759 3.751 3.748 3.742 3.734 3.728 3.654 3.219 1.648 1.643 1.638 1.632 1.624 1.618 1.614 1.564 1.557 1.548 1.543 1.539 1.534 1.524 1.515 1.509 1.502 1.497 1.489 1.482 1.472 1.459 1.453 1.443 1.439 1.429 1.425 1.107 1.094 0.969 0.954 0.940
NAME 05-NHR_1_176(winreb amide)
EXPNO 10
PROCNO 1
Date_ 20240105
Time 18.50 h
INSTRUM Avance NEO 500
PROBHD Z119470_0332 (
PULPROG zg30
TD 65536
SOLVENT CDCl3
NS 8
DS 2
SWH 10000.000 Hz
FIDRES 0.305176 Hz
AQ 3.2768500 sec
RG 94.5455
DW 50.000 usec
DE 10.84 usec
TE 298.0 K
D1 1.00000000 sec
TD0 1
SFO1 500.1530884 MHz
NUC1 1H
P0 3.24 usec
P1 9.72 usec
SI 65536
SF 500.1500000 MHz
WDW EM
SSB 0
LB 0.30 Hz
GB 0
PC 1.00
O
OBn
O
N
S1
9 8 7 6 5 4 3 2 1 0 ppm
5.162 1.793 1.090 2.984 4.000 1.054 3.095 3.138 3.229

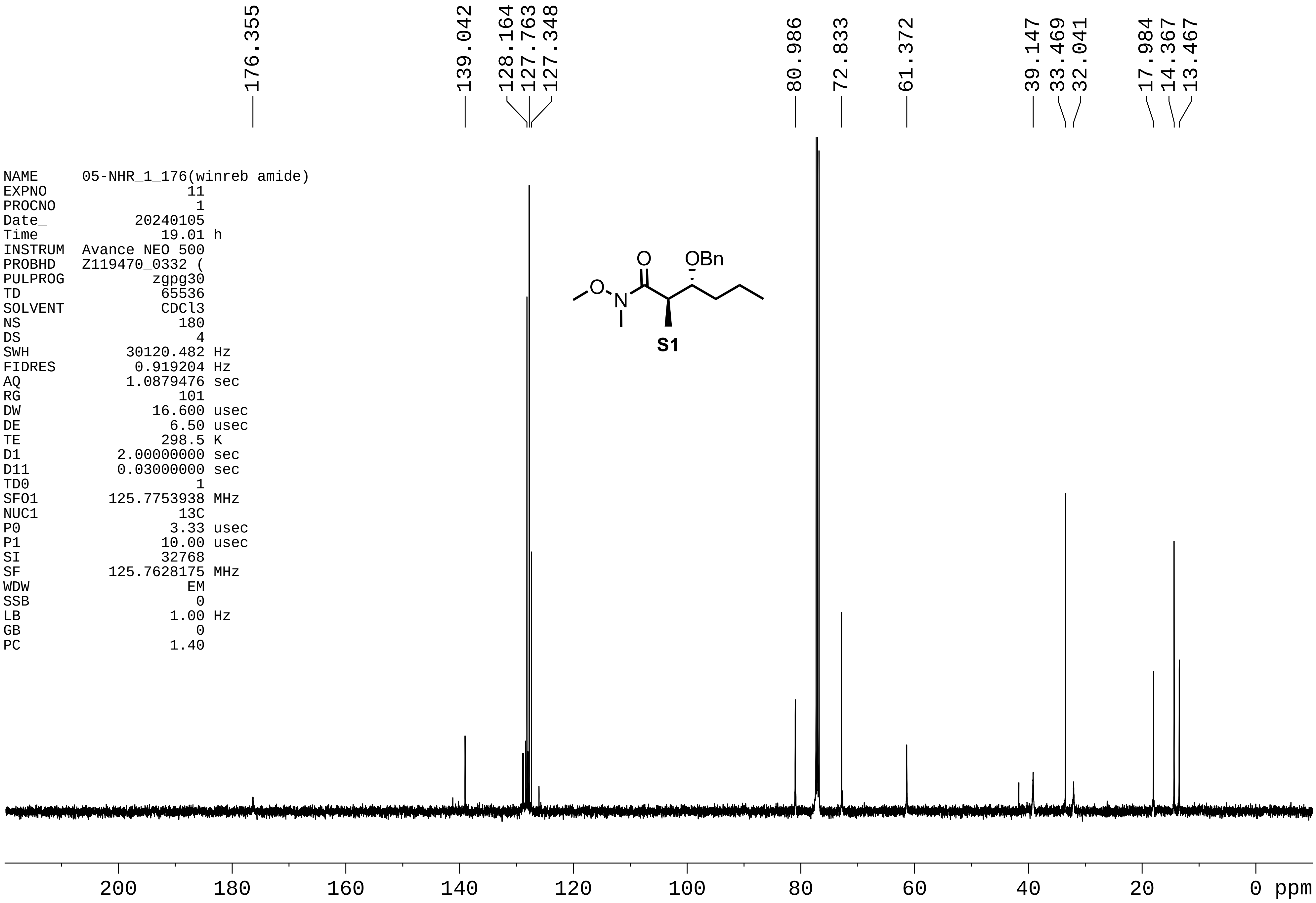
176.355
139.042
128.164
127.763
127.348
80.986
72.833
61.372
39.147
33.469
32.041
17.984
14.367
13.467
NAME 05-NHR_1_176(winreb amide)
EXPNO 11
PROCNO 1
Date_ 20240105
Time 19.01 h
INSTRUM Avance NEO 500
PROBHD Z119470_0332 (
PULPROG zgpg30
TD 65536
SOLVENT CDCl3
NS 180
DS 4
SWH 30120.482 Hz
FIDRES 0.919204 Hz
AQ 1.0879476 sec
RG 101
DW 16.600 usec
DE 6.50 usec
TE 298.5 K
D1 2.00000000 sec
D11 0.03000000 sec
TD0 1
SFO1 125.7753938 MHz
NUC1 13C
P0 3.33 usec
P1 10.00 usec
SI 32768
SF 125.7628175 MHz
WDW EM
SSB 0
LB 1.00 Hz
GB 0
PC 1.40
O
OBn
O
N
S1
200
180
160
140
120
100
80
60
40
20
0 ppm

NAME	06-NHR_1_082(3) ketone
EXPNO	10
PROCNO	1
Date_	20230719
Time	8.39 h
INSTRUM	Avance NEO 500
PROBHD	Z119470_0332 (
PULPROG	zg30
TD	65536
SOLVENT	CDCl3
NS	8
DS	2
SWH	10000.000 Hz
FIDRES	0.305176 Hz
AQ	3.2768500 sec
RG	80
DW	50.000 usec
DE	10.84 usec
TE	296.3 K
D1	1.00000000 sec
TD0	1
SFO1	500.1530884 MHz
NUC1	1H
P0	3.24 usec
P1	9.72 usec
SI	65536
SF	500.1500000 MHz
WDW	EM
SSB	0
LB	0.30 Hz
GB	0
PC	1.00

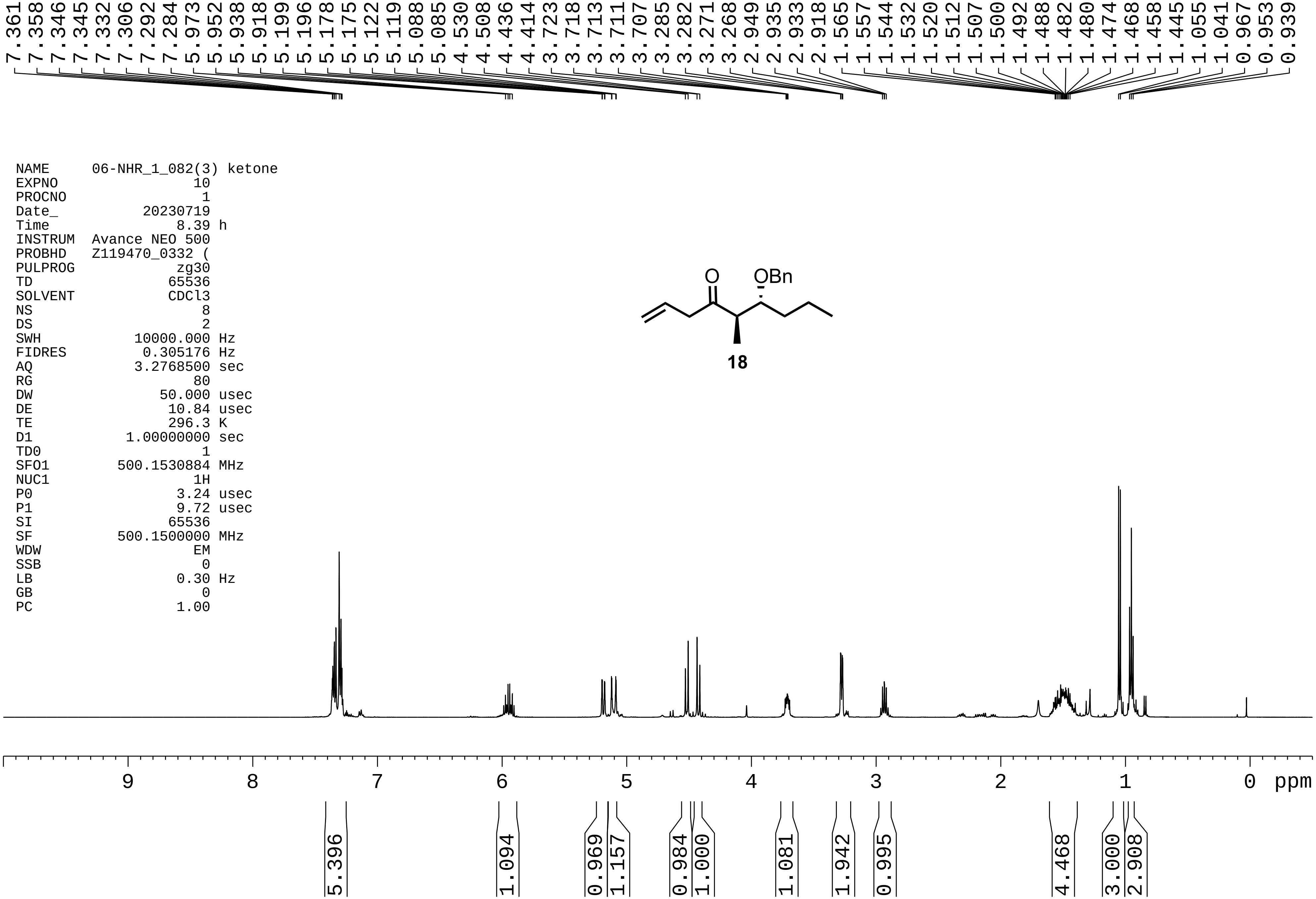

```
NAME     06-NHR_1_082(3) ketone
EXPNO              11
PROCNO              1
Date_        20230719
Time             8.49 h
INSTRUM  Avance NEO 500
PROBHD   Z119470_0332 (
PULPROG        zgpg30
TD              65536
SOLVENT         CDCl3
NS                188
DS                  4
SWH         30120.482 Hz
FIDRES       0.919204 Hz
AQ          1.0879476 sec
RG                101
DW             16.600 usec
DE               6.50 usec
TE              296.8 K
D1         2.00000000 sec
D11        0.03000000 sec
TD0                 1
SFO1      125.7753938 MHz
NUC1              13C
P0               3.33 usec
P1              10.00 usec
SI              32768
SF        125.7628175 MHz
WDW                EM
SSB                 0
LB               1.00 Hz
GB                  0
PC               1.40
```

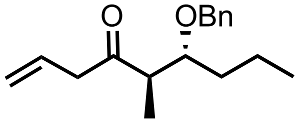

18

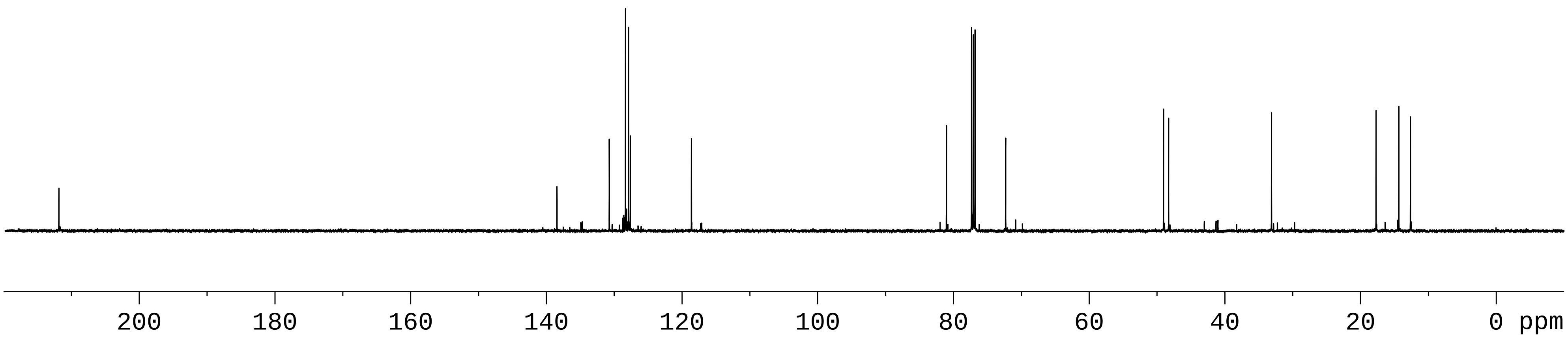

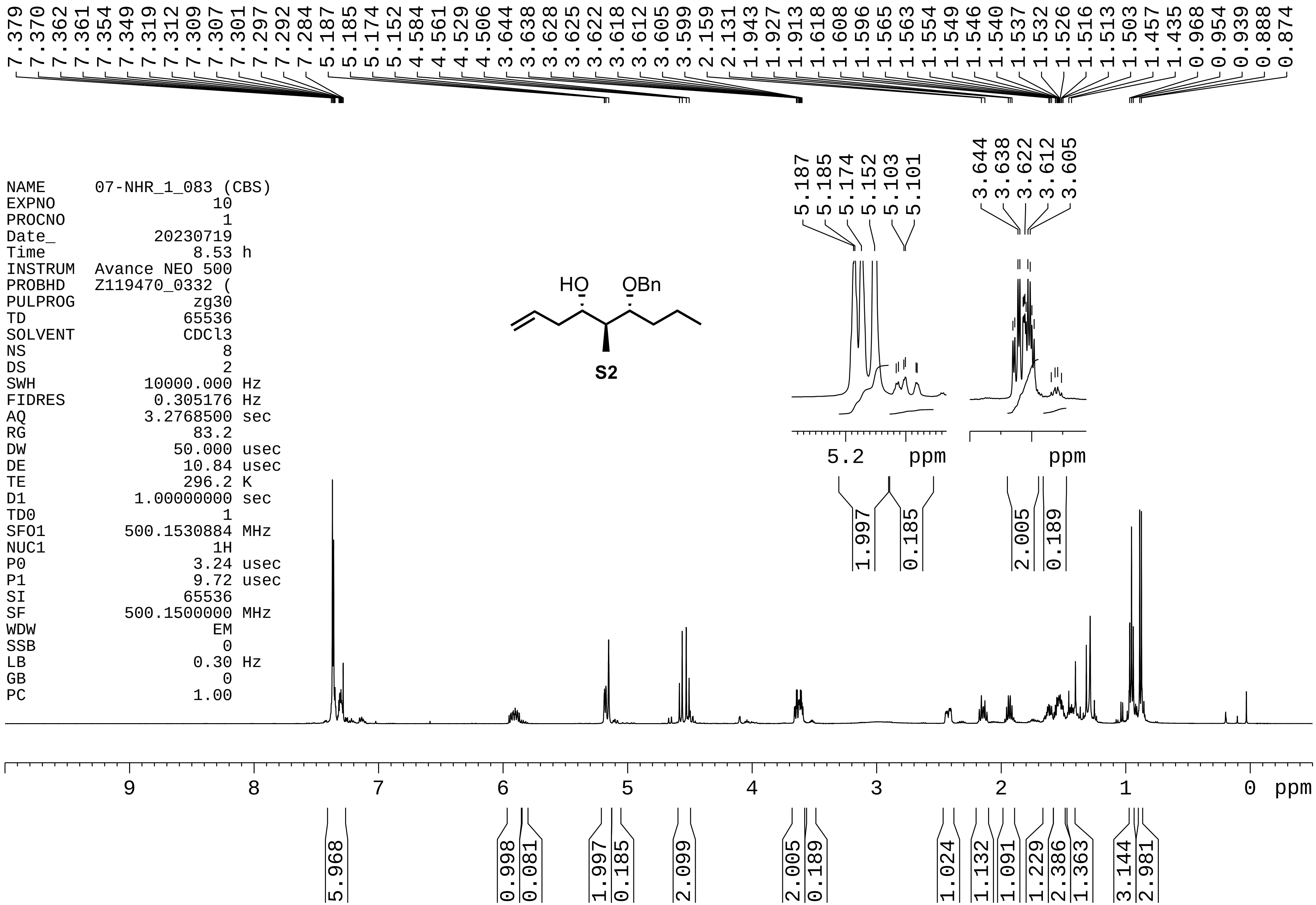
7.379 7.370 7.362 7.361 7.354 7.349 7.319 7.312 7.309 7.307 7.301 7.297 7.292 7.284 5.187 5.185 5.174 5.152 4.584 4.561 4.529 4.506 3.644 3.638 3.628 3.625 3.622 3.618 3.612 3.605 3.599 2.159 2.131 1.943 1.927 1.913 1.618 1.608 1.596 1.565 1.563 1.554 1.549 1.546 1.540 1.537 1.532 1.526 1.516 1.513 1.503 1.457 1.435 0.968 0.954 0.939 0.888 0.874
5.187 5.185 5.174 5.152 5.103 5.101
3.644 3.638 3.622 3.612 3.605
NAME 07-NHR_1_083 (CBS)
EXPNO 10
PROCNO 1
Date_ 20230719
Time 8.53 h
INSTRUM Avance NEO 500
PROBHD Z119470_0332 (
PULPROG zg30
TD 65536
SOLVENT CDCl3
NS 8
DS 2
SWH 10000.000 Hz
FIDRES 0.305176 Hz
AQ 3.2768500 sec
RG 83.2
DW 50.000 usec
DE 10.84 usec
TE 296.2 K
D1 1.00000000 sec
TD0 1
SFO1 500.1530884 MHz
NUC1 1H
P0 3.24 usec
P1 9.72 usec
SI 65536
SF 500.1500000 MHz
WDW EM
SSB 0
LB 0.30 Hz
GB 0
PC 1.00
HO
OBn
S2
5.2 ppm
ppm
1.997 0.185
2.005 0.189
9 8 7 6 5 4 3 2 1 0 ppm
5.968 0.998 0.081 1.997 0.185 2.099 2.005 0.189 1.024 1.132 1.091 1.229 2.386 1.363 3.144 2.981

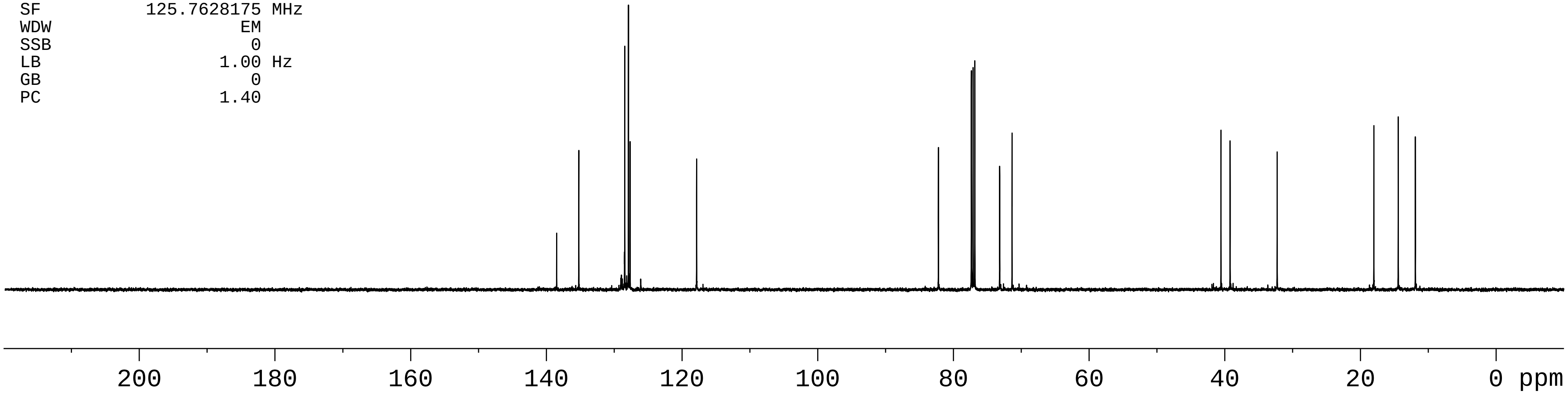

NAME	07-NHR_1_179(2.26) CBS
EXPNO	11
PROCNO	1
Date_	20240305
Time	11.10 h
INSTRUM	Avance NEO 500
PROBHD	Z119470_0332 (
PULPROG	zgpg30
TD	65536
SOLVENT	CDCl3
NS	200
DS	4
SWH	30120.482 Hz
FIDRES	0.919204 Hz
AQ	1.0879476 sec
RG	101
DW	16.600 usec
DE	6.50 usec
TE	294.8 K
D1	2.00000000 sec
D11	0.03000000 sec
TD0	1
SFO1	125.7753938 MHz
NUC1	13C
P0	3.33 usec
P1	10.00 usec
SI	32768
SF	125.7628175 MHz
WDW	EM
SSB	0
LB	1.00 Hz
GB	0
PC	1.40

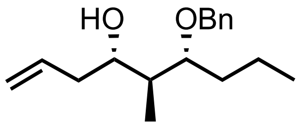

S2

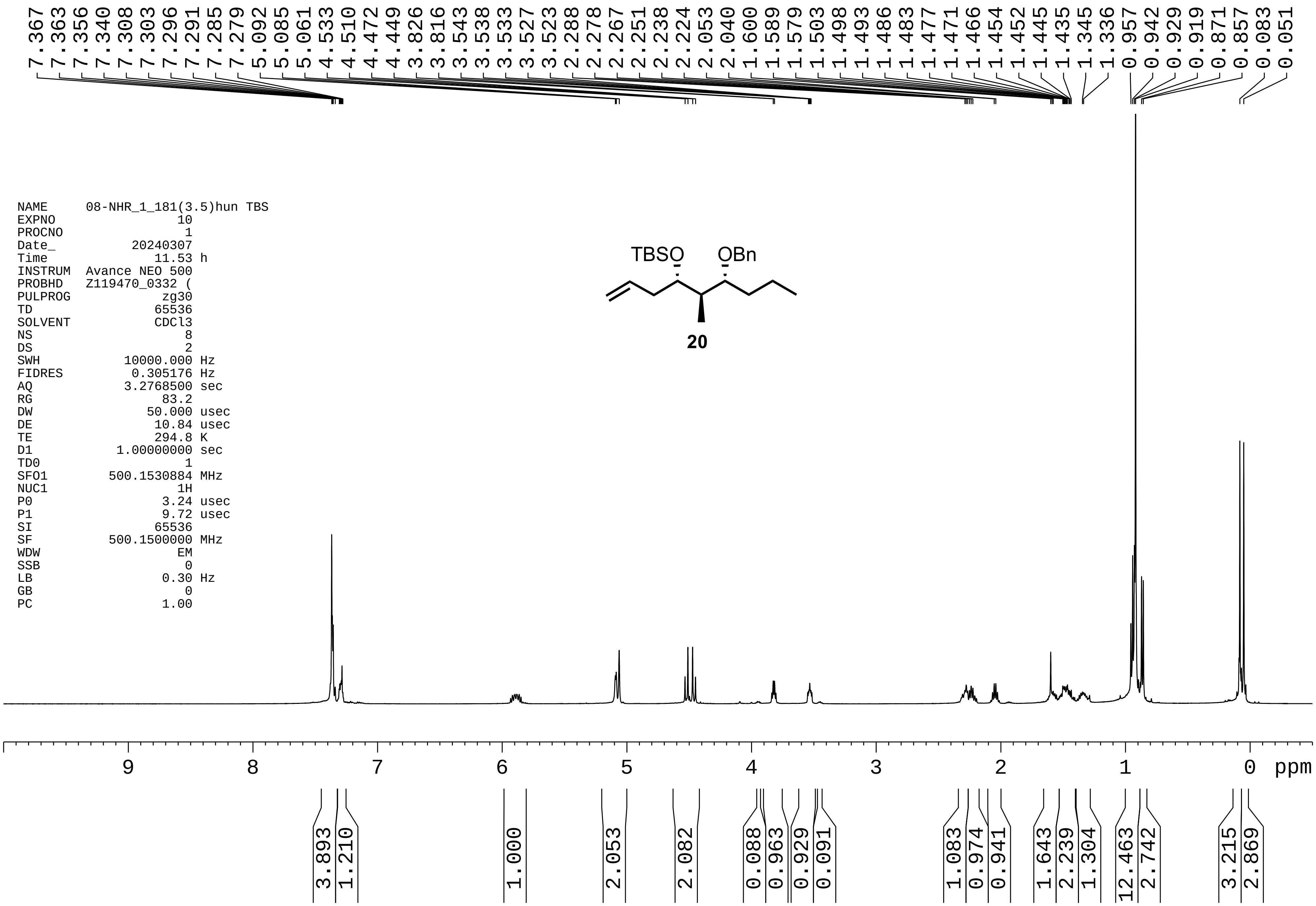
7.367 7.363 7.356 7.340 7.308 7.303 7.296 7.291 7.285 7.279 5.092 5.085 5.061 4.533 4.510 4.472 4.449 3.826 3.816 3.543 3.538 3.533 3.527 3.523 2.288 2.278 2.267 2.251 2.238 2.224 2.053 2.040 1.600 1.589 1.579 1.503 1.498 1.493 1.486 1.483 1.477 1.471 1.466 1.454 1.452 1.445 1.435 1.345 1.336 0.957 0.942 0.929 0.919 0.871 0.857 0.083 0.051
NAME 08-NHR_1_181(3.5)hun TBS
EXPNO 10
PROCNO 1
Date_ 20240307
Time 11.53 h
INSTRUM Avance NEO 500
PROBHD Z119470_0332 (
PULPROG zg30
TD 65536
SOLVENT CDCl3
NS 8
DS 2
SWH 10000.000 Hz
FIDRES 0.305176 Hz
AQ 3.2768500 sec
RG 83.2
DW 50.000 usec
DE 10.84 usec
TE 294.8 K
D1 1.00000000 sec
TD0 1
SFO1 500.1530884 MHz
NUC1 1H
P0 3.24 usec
P1 9.72 usec
SI 65536
SF 500.1500000 MHz
WDW EM
SSB 0
LB 0.30 Hz
GB 0
PC 1.00
TBSO
OBn
20
9 8 7 6 5 4 3 2 1 0 ppm
3.893 1.210 1.000 2.053 2.082 0.088 0.963 0.929 0.091 1.083 0.974 0.941 1.643 2.239 1.304 12.463 2.742 3.215 2.869

NAME 08-NHR_1_181(3.5)hun TBS 1.8
EXPNO 11
PROCNO 1
Date_ 20240307
Time 12.04 h
INSTRUM Avance NEO 500
PROBHD Z119470_0332 (
PULPROG zgpg30
TD 65536
SOLVENT CDCl3
NS 200
DS 4
SWH 30120.482 Hz
FIDRES 0.919204 Hz
AQ 1.0879476 sec
RG 101
DW 16.600 usec
DE 6.50 usec
TE 295.5 K
D1 2.00000000 sec
D11 0.03000000 sec
TD0 1
SFO1 125.7753938 MHz
NUC1 13C
P0 3.33 usec
P1 10.00 usec
SI 32768
SF 125.7628175 MHz
WDW EM
SSB 0
LB 1.00 Hz
GB 0
PC 1.40

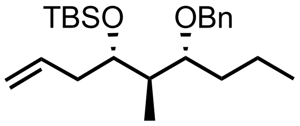

20

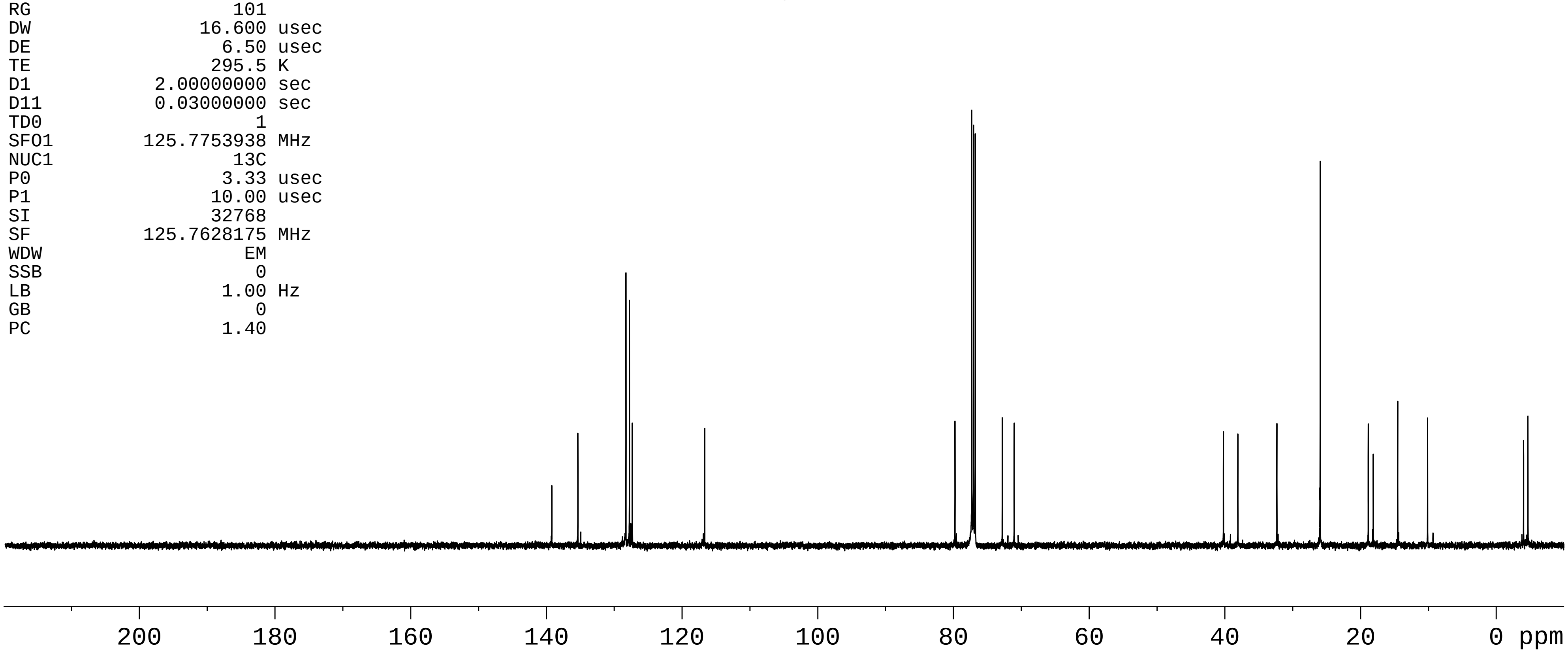

```
NAME       09-NHR_1_182(3.7)DDQ de Bn
EXPNO                  10
PROCNO                  1
Date_            20240308
Time                11.42 h
INSTRUM    Avance NEO 500
PROBHD     Z119470_0332 (
PULPROG              zg30
TD                  65536
SOLVENT             CDCl3
NS                      8
DS                      2
SWH             10000.000 Hz
FIDRES           0.305176 Hz
AQ              3.2768500 sec
RG                    101
DW                 50.000 usec
DE                  10.84 usec
TE                  294.6 K
D1             1.00000000 sec
TD0                     1
SFO1          500.1530884 MHz
NUC1                   1H
P0                   3.24 usec
P1                   9.72 usec
SI                  65536
SF            500.1500000 MHz
WDW                    EM
SSB                     0
LB                   0.30 Hz
GB                      0
PC                   1.00
```

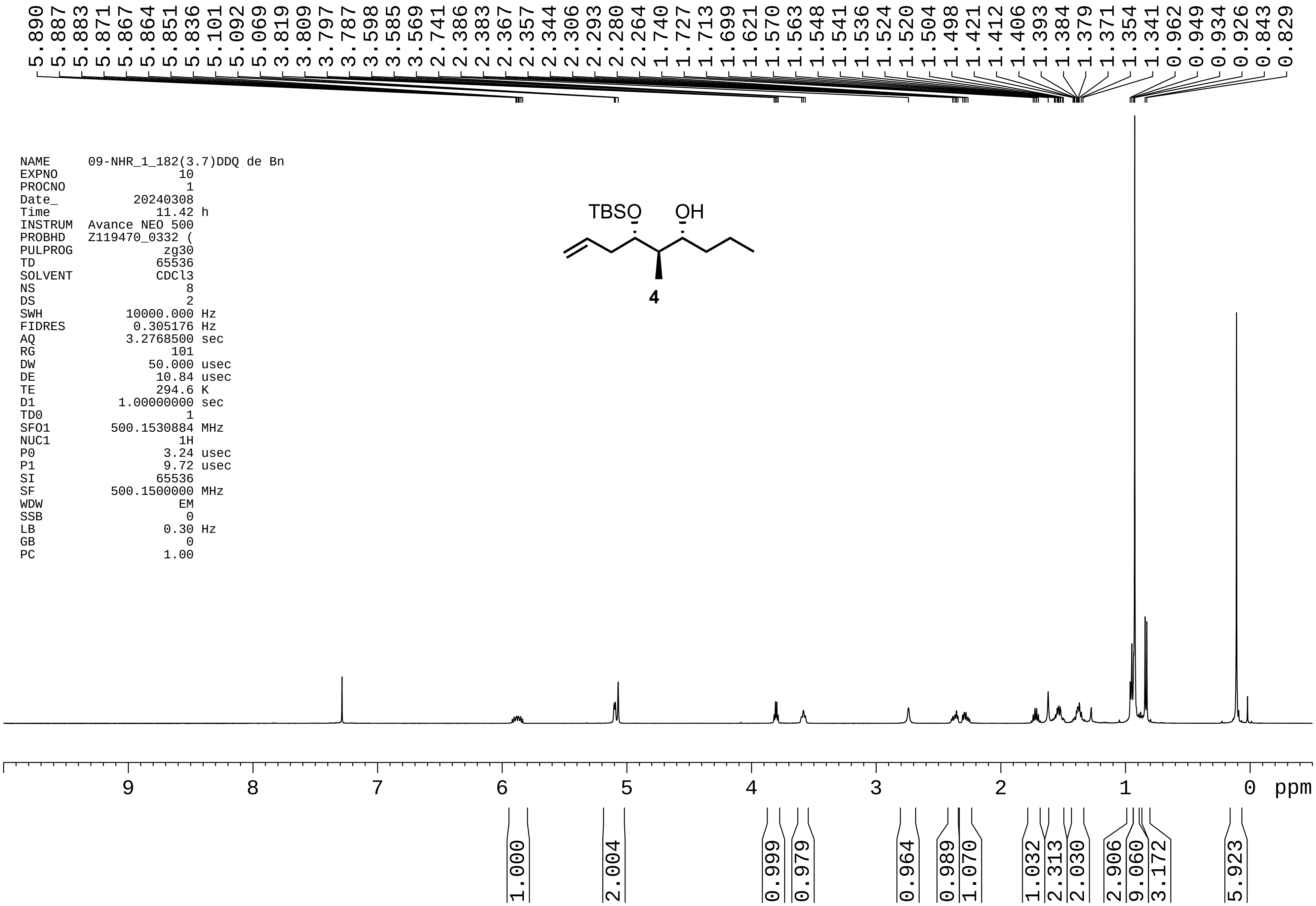

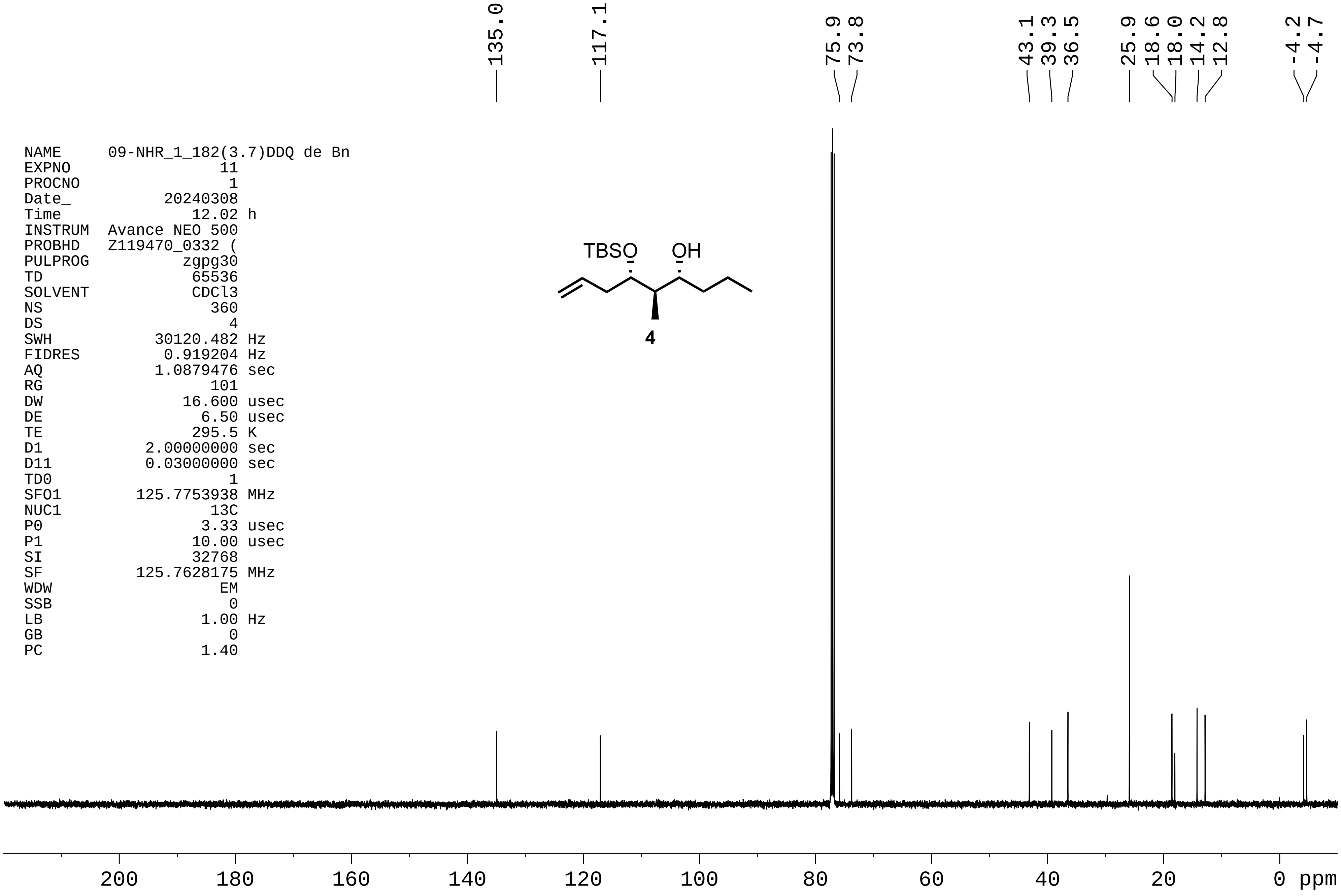

135.0
117.1
75.9
73.8
43.1
39.3
36.5
25.9
18.6
18.0
14.2
12.8
-4.2
-4.7
NAME 09-NHR_1_182(3.7)DDQ de Bn
EXPNO 11
PROCNO 1
Date_ 20240308
Time 12.02 h
INSTRUM Avance NEO 500
PROBHD Z119470_0332 (
PULPROG zgpg30
TD 65536
SOLVENT CDCl3
NS 360
DS 4
SWH 30120.482 Hz
FIDRES 0.919204 Hz
AQ 1.0879476 sec
RG 101
DW 16.600 usec
DE 6.50 usec
TE 295.5 K
D1 2.00000000 sec
D11 0.03000000 sec
TD0 1
SFO1 125.7753938 MHz
NUC1 13C
P0 3.33 usec
P1 10.00 usec
SI 32768
SF 125.7628175 MHz
WDW EM
SSB 0
LB 1.00 Hz
GB 0
PC 1.40
TBSO
OH
4
200
180
160
140
120
100
80
60
40
20
0 ppm

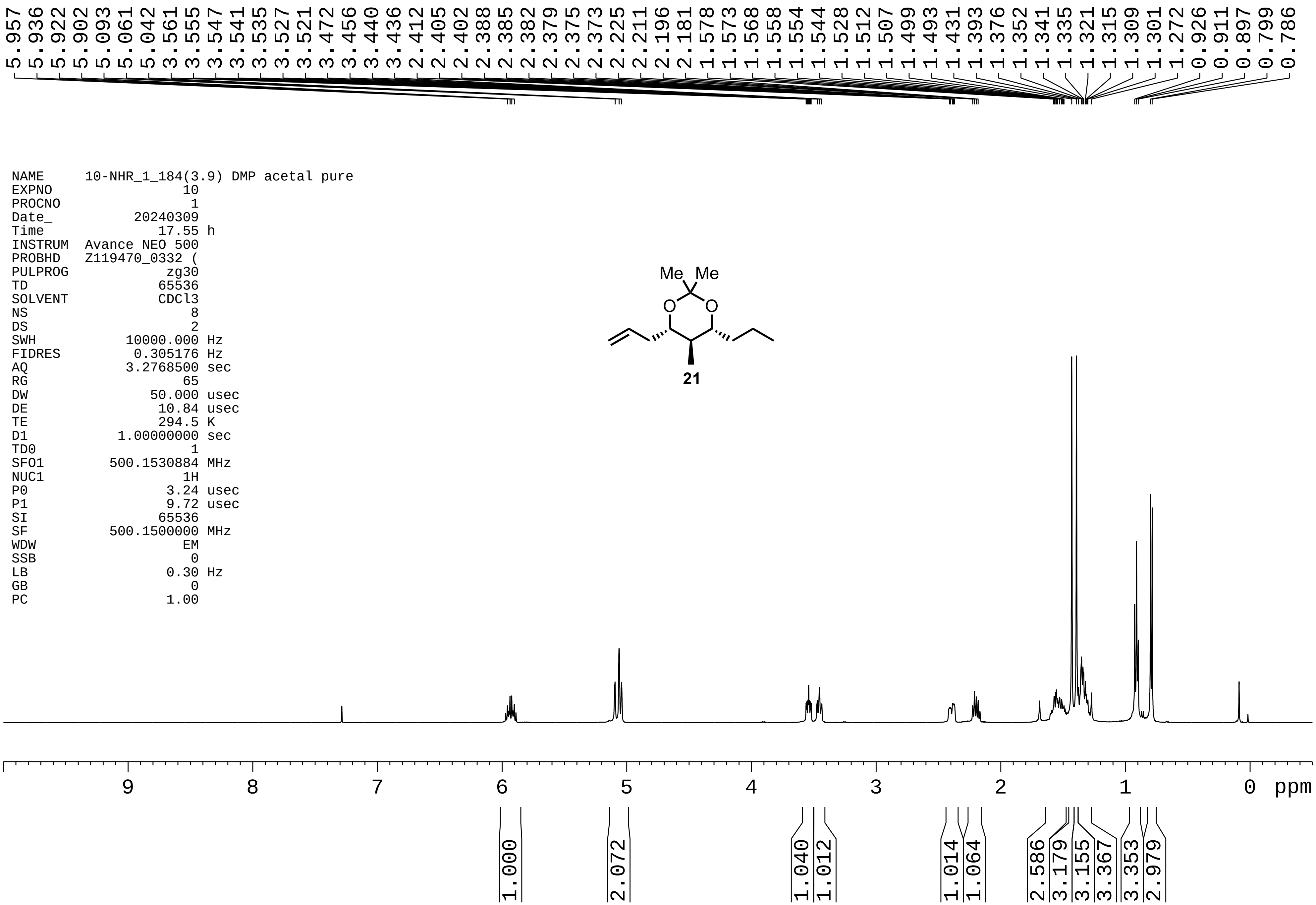
5.957 5.936 5.922 5.902 5.093 5.061 5.042 3.561 3.555 3.547 3.541 3.535 3.527 3.521 3.472 3.456 3.440 3.436 2.412 2.405 2.402 2.388 2.385 2.382 2.379 2.375 2.373 2.225 2.211 2.196 2.181 1.578 1.573 1.568 1.558 1.554 1.544 1.528 1.512 1.507 1.499 1.493 1.431 1.393 1.376 1.352 1.341 1.335 1.321 1.315 1.309 1.301 1.272 0.926 0.911 0.897 0.799 0.786
NAME 10-NHR_1_184(3.9) DMP acetal pure
EXPNO 10
PROCNO 1
Date_ 20240309
Time 17.55 h
INSTRUM Avance NEO 500
PROBHD Z119470_0332 (
PULPROG zg30
TD 65536
SOLVENT CDCl3
NS 8
DS 2
SWH 10000.000 Hz
FIDRES 0.305176 Hz
AQ 3.2768500 sec
RG 65
DW 50.000 usec
DE 10.84 usec
TE 294.5 K
D1 1.00000000 sec
TD0 1
SFO1 500.1530884 MHz
NUC1 1H
P0 3.24 usec
P1 9.72 usec
SI 65536
SF 500.1500000 MHz
WDW EM
SSB 0
LB 0.30 Hz
GB 0
PC 1.00
Me Me
O O
21
9 8 7 6 5 4 3 2 1 0 ppm
1.000 2.072 1.040 1.012 1.014 1.064 2.586 3.179 3.155 3.367 3.353 2.979

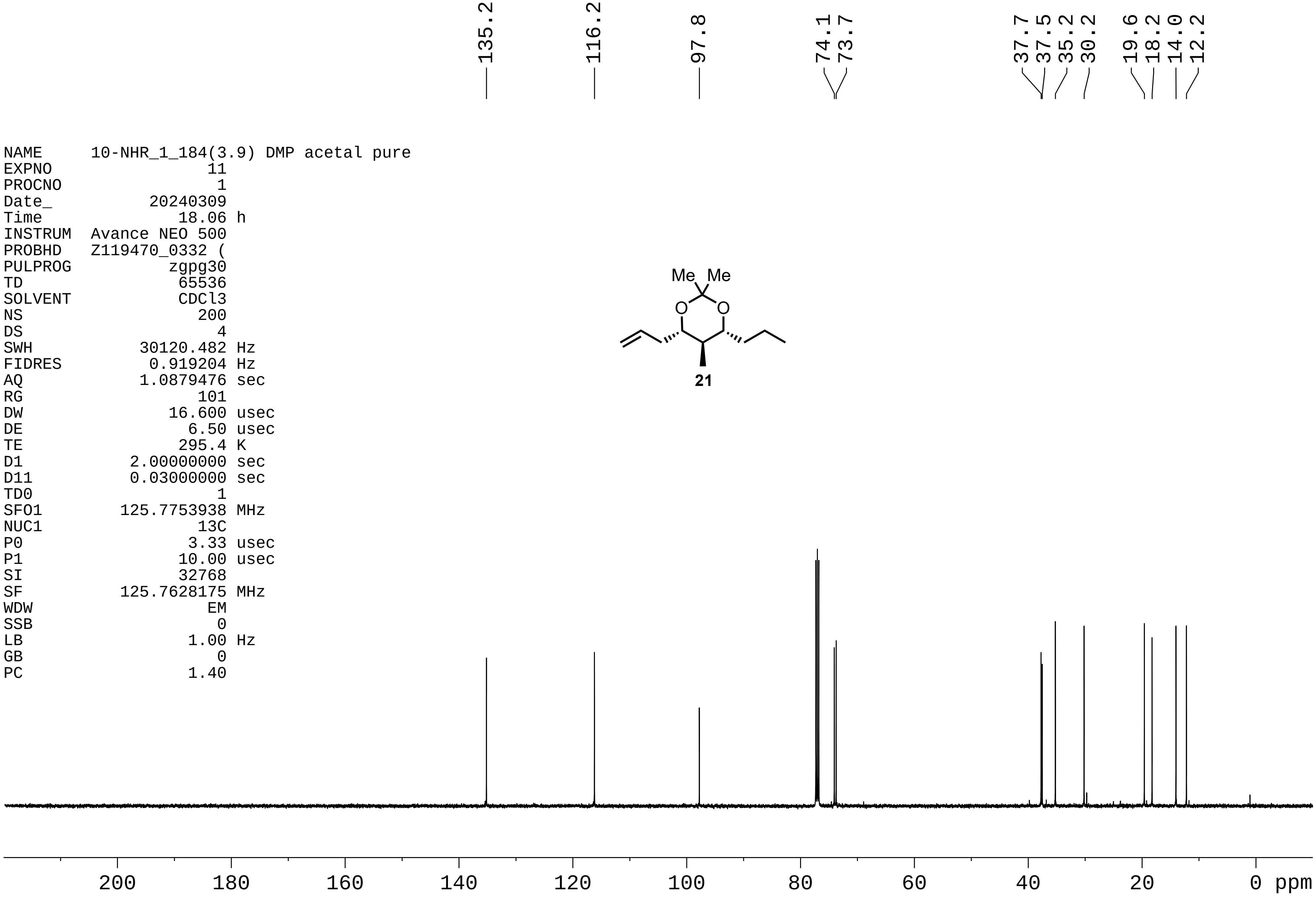
135.2
116.2
97.8
74.1
73.7
37.7
37.5
35.2
30.2
19.6
18.2
14.0
12.2
NAME 10-NHR_1_184(3.9) DMP acetal pure
EXPNO 11
PROCNO 1
Date_ 20240309
Time 18.06 h
INSTRUM Avance NEO 500
PROBHD Z119470_0332 (
PULPROG zgpg30
TD 65536
SOLVENT CDCl3
NS 200
DS 4
SWH 30120.482 Hz
FIDRES 0.919204 Hz
AQ 1.0879476 sec
RG 101
DW 16.600 usec
DE 6.50 usec
TE 295.4 K
D1 2.00000000 sec
D11 0.03000000 sec
TD0 1
SFO1 125.7753938 MHz
NUC1 13C
P0 3.33 usec
P1 10.00 usec
SI 32768
SF 125.7628175 MHz
WDW EM
SSB 0
LB 1.00 Hz
GB 0
PC 1.40
Me
Me
O
O
21
200
180
160
140
120
100
80
60
40
20
0 ppm

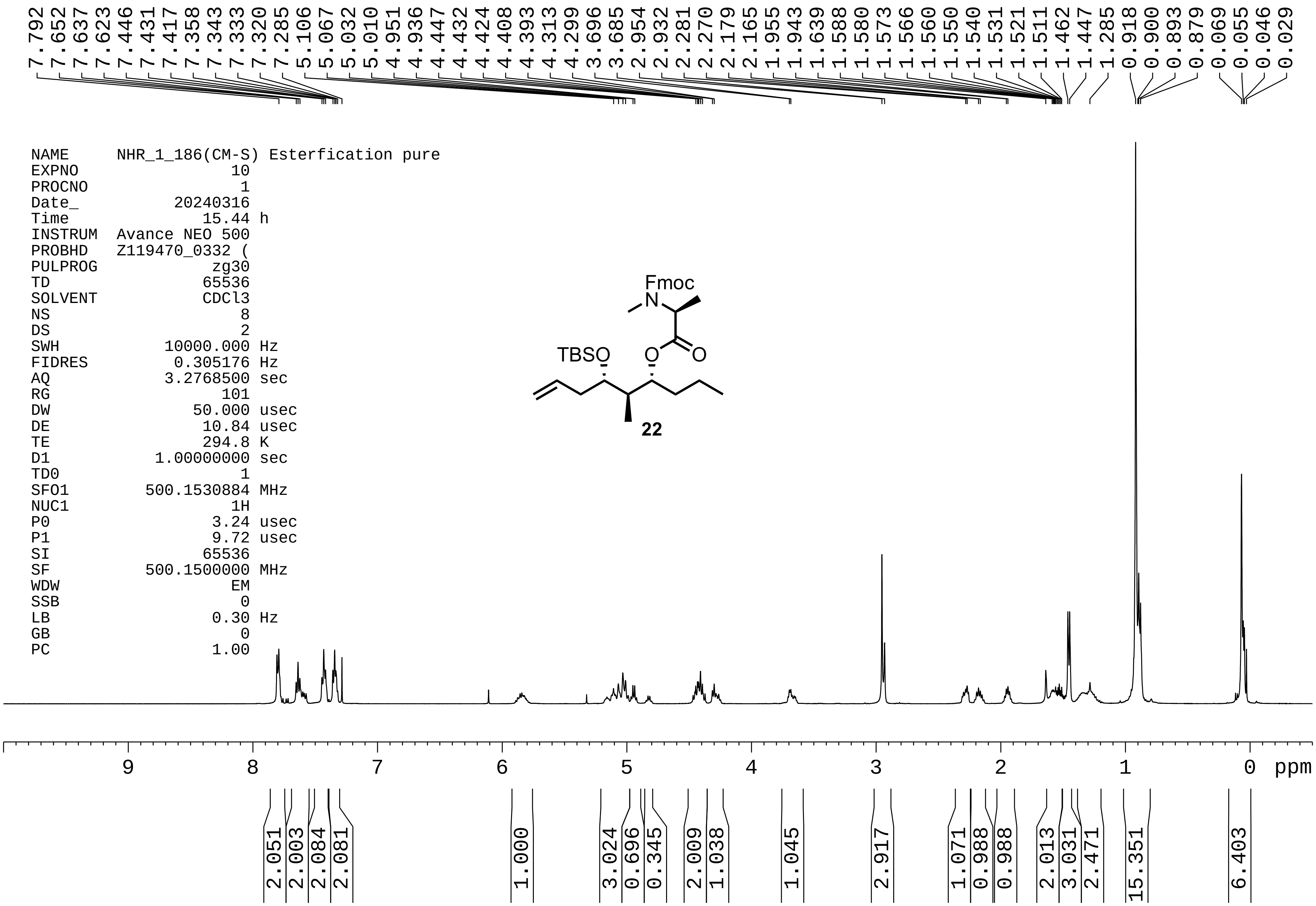
7.792
7.652
7.637
7.623
7.446
7.431
7.417
7.358
7.343
7.333
7.320
7.285
5.106
5.067
5.032
5.010
4.951
4.936
4.447
4.432
4.424
4.408
4.393
4.313
4.299
3.696
3.685
2.954
2.932
2.281
2.270
2.179
2.165
1.955
1.943
1.639
1.588
1.580
1.573
1.566
1.560
1.550
1.540
1.531
1.521
1.511
1.462
1.447
1.285
0.918
0.900
0.893
0.879
0.069
0.055
0.046
0.029
NAME NHR_1_186(CM-S) Esterfication pure
EXPNO 10
PROCNO 1
Date_ 20240316
Time 15.44 h
INSTRUM Avance NEO 500
PROBHD Z119470_0332 (
PULPROG zg30
TD 65536
SOLVENT CDCl3
NS 8
DS 2
SWH 10000.000 Hz
FIDRES 0.305176 Hz
AQ 3.2768500 sec
RG 101
DW 50.000 usec
DE 10.84 usec
TE 294.8 K
D1 1.00000000 sec
TD0 1
SFO1 500.1530884 MHz
NUC1 1H
P0 3.24 usec
P1 9.72 usec
SI 65536
SF 500.1500000 MHz
WDW EM
SSB 0
LB 0.30 Hz
GB 0
PC 1.00
Fmoc
N
TBSO
O
O
22
9
8
7
6
5
4
3
2
1
0
ppm
2.051
2.003
2.084
2.081
1.000
3.024
0.696
0.345
2.009
1.038
1.045
2.917
1.071
0.988
0.988
2.013
3.031
2.471
15.351
6.403

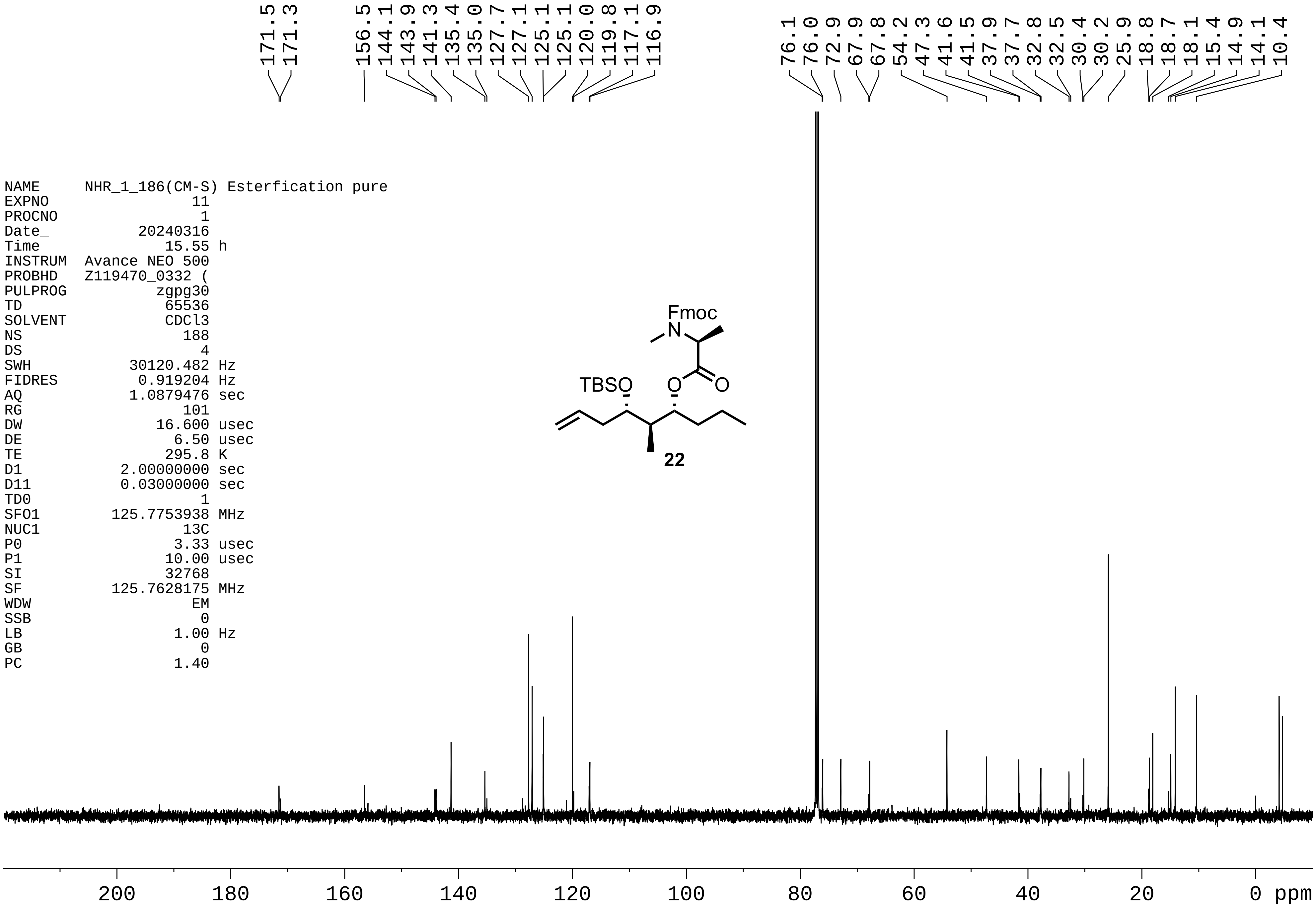

22

```
NAME      NHR_1_186(CM-S) Esterfication pure
EXPNO                  11
PROCNO                  1
Date_            20240316
Time                15.55 h
INSTRUM   Avance NEO 500
PROBHD    Z119470_0332 (
PULPROG           zgpg30
TD                 65536
SOLVENT            CDCl3
NS                   188
DS                     4
SWH           30120.482 Hz
FIDRES         0.919204 Hz
AQ            1.0879476 sec
RG                   101
DW               16.600 usec
DE                 6.50 usec
TE                295.8 K
D1           2.00000000 sec
D11          0.03000000 sec
TD0                    1
SFO1        125.7753938 MHz
NUC1                 13C
P0                  3.33 usec
P1                 10.00 usec
SI                 32768
SF          125.7628175 MHz
WDW                   EM
SSB                    0
LB                  1.00 Hz
GB                     0
PC                  1.40
```

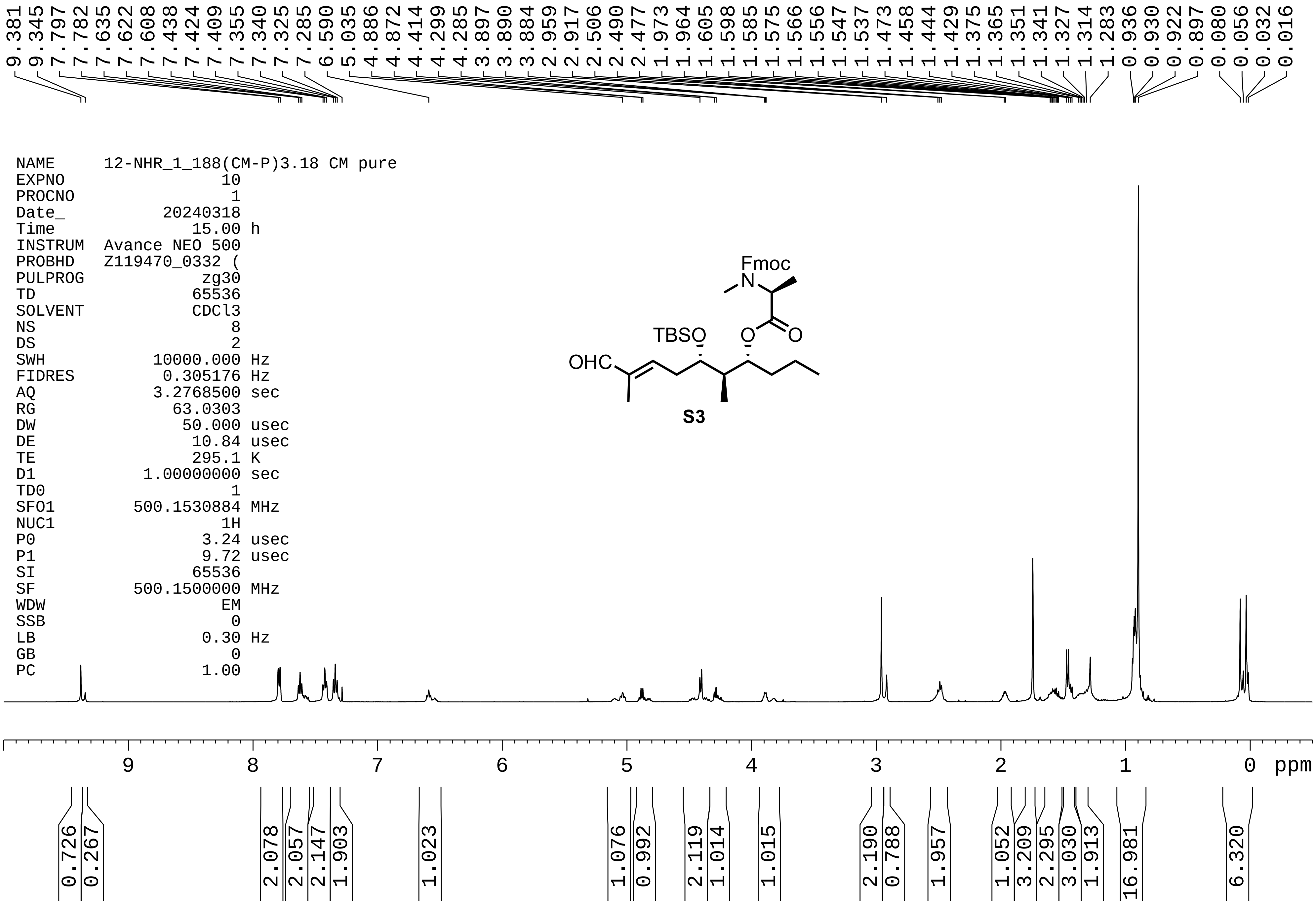
9.381
9.345
7.797
7.782
7.635
7.622
7.608
7.438
7.424
7.409
7.355
7.340
7.325
7.285
6.590
5.035
4.886
4.872
4.414
4.299
4.285
3.897
3.890
3.884
2.959
2.917
2.506
2.490
2.477
1.973
1.964
1.605
1.598
1.585
1.575
1.566
1.556
1.547
1.537
1.473
1.458
1.444
1.429
1.375
1.365
1.351
1.341
1.327
1.314
1.283
0.936
0.930
0.922
0.897
0.080
0.056
0.032
0.016
NAME 12-NHR_1_188(CM-P)3.18 CM pure
EXPNO 10
PROCNO 1
Date_ 20240318
Time 15.00 h
INSTRUM Avance NEO 500
PROBHD Z119470_0332 (
PULPROG zg30
TD 65536
SOLVENT CDCl3
NS 8
DS 2
SWH 10000.000 Hz
FIDRES 0.305176 Hz
AQ 3.2768500 sec
RG 63.0303
DW 50.000 usec
DE 10.84 usec
TE 295.1 K
D1 1.00000000 sec
TD0 1
SFO1 500.1530884 MHz
NUC1 1H
P0 3.24 usec
P1 9.72 usec
SI 65536
SF 500.1500000 MHz
WDW EM
SSB 0
LB 0.30 Hz
GB 0
PC 1.00
9
8
7
6
5
4
3
2
1
0 ppm
0.726
0.267
2.078
2.057
2.147
1.903
1.023
1.076
0.992
2.119
1.014
1.015
2.190
0.788
1.957
1.052
3.209
2.295
3.030
1.913
16.981
6.320
Fmoc
N
O
O
TBSO
O
OHC

S3

```
NAME      12-NHR_1_188(CM-P)3.18 CM pure
EXPNO                    11
PROCNO                    1
Date_              20240318
Time                  15.11 h
INSTRUM  Avance NEO 500
PROBHD   Z119470_0332 (
PULPROG              zgpg30
TD                    65536
SOLVENT               CDCl3
NS                      188
DS                        4
SWH               30120.482 Hz
FIDRES             0.919204 Hz
AQ                1.0879476 sec
RG                      101
DW                   16.600 usec
DE                     6.50 usec
TE                    295.8 K
D1               2.00000000 sec
D11              0.03000000 sec
TD0                       1
SFO1            125.7753938 MHz
NUC1                    13C
P0                     3.33 usec
P1                    10.00 usec
SI                    32768
SF              125.7628175 MHz
WDW                      EM
SSB                       0
LB                     1.00 Hz
GB                        0
PC                     1.40
```

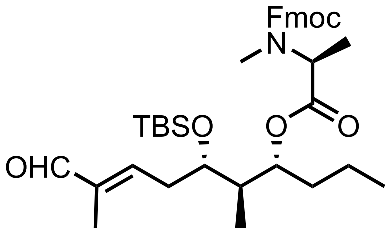

S3

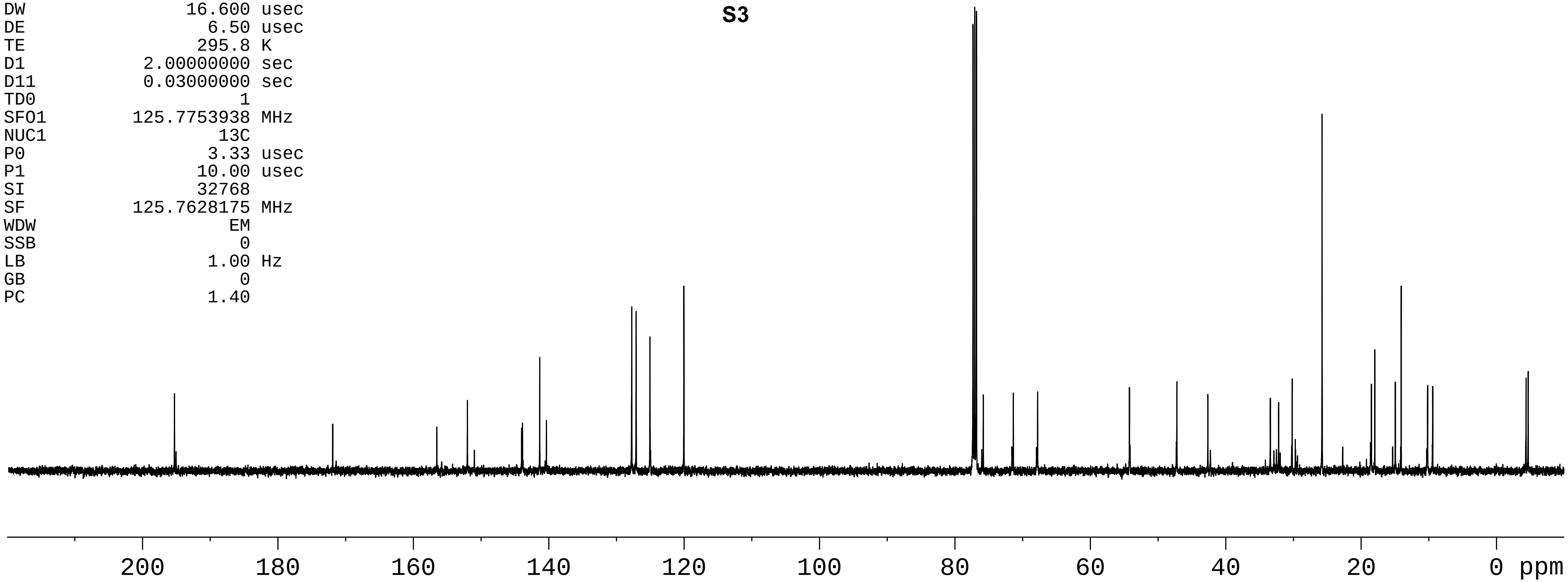

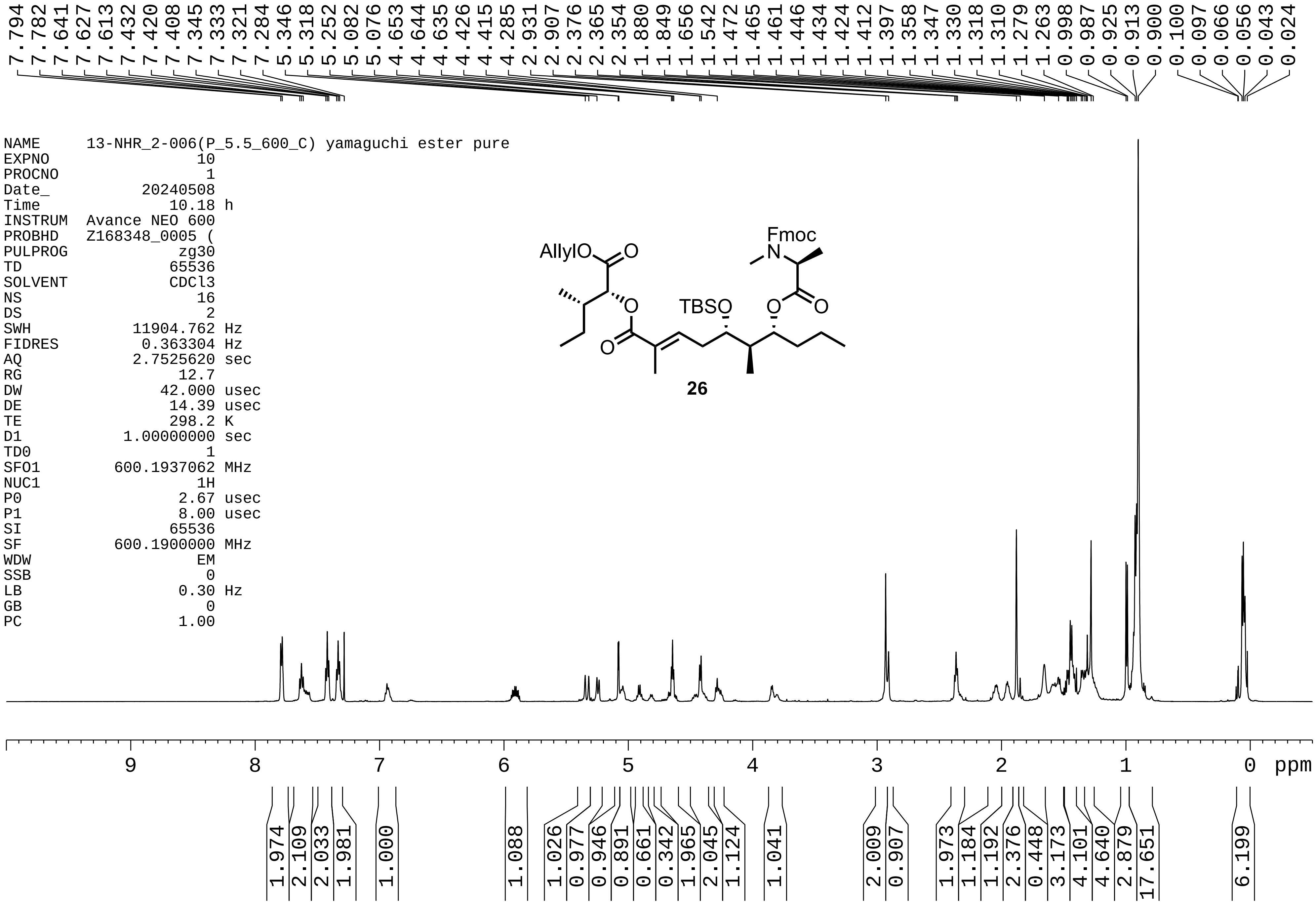
7.794 7.782 7.641 7.627 7.613 7.432 7.420 7.408 7.345 7.333 7.321 7.284 5.346 5.318 5.252 5.082 5.076 4.653 4.644 4.635 4.426 4.415 4.285 2.931 2.907 2.376 2.365 2.354 1.880 1.849 1.656 1.542 1.472 1.465 1.461 1.446 1.434 1.424 1.412 1.397 1.358 1.347 1.330 1.318 1.310 1.279 1.263 0.998 0.987 0.925 0.913 0.900 0.100 0.097 0.066 0.056 0.043 0.024
NAME 13-NHR_2-006(P_5.5_600_C) yamaguchi ester pure
EXPNO 10
PROCNO 1
Date_ 20240508
Time 10.18 h
INSTRUM Avance NEO 600
PROBHD Z168348_0005 (
PULPROG zg30
TD 65536
SOLVENT CDCl3
NS 16
DS 2
SWH 11904.762 Hz
FIDRES 0.363304 Hz
AQ 2.7525620 sec
RG 12.7
DW 42.000 usec
DE 14.39 usec
TE 298.2 K
D1 1.00000000 sec
TD0 1
SFO1 600.1937062 MHz
NUC1 1H
P0 2.67 usec
P1 8.00 usec
SI 65536
SF 600.1900000 MHz
WDW EM
SSB 0
LB 0.30 Hz
GB 0
PC 1.00
Fmoc
AllylO
O
N
TBSO
O
O
O
26
9 8 7 6 5 4 3 2 1 0 ppm
1.974 2.109 2.033 1.981 1.000 1.088 1.026 0.977 0.946 0.891 0.661 0.342 1.965 2.045 1.124 1.041 2.009 0.907 1.973 1.184 1.192 2.376 0.448 3.173 4.101 4.640 2.879 17.651 6.199

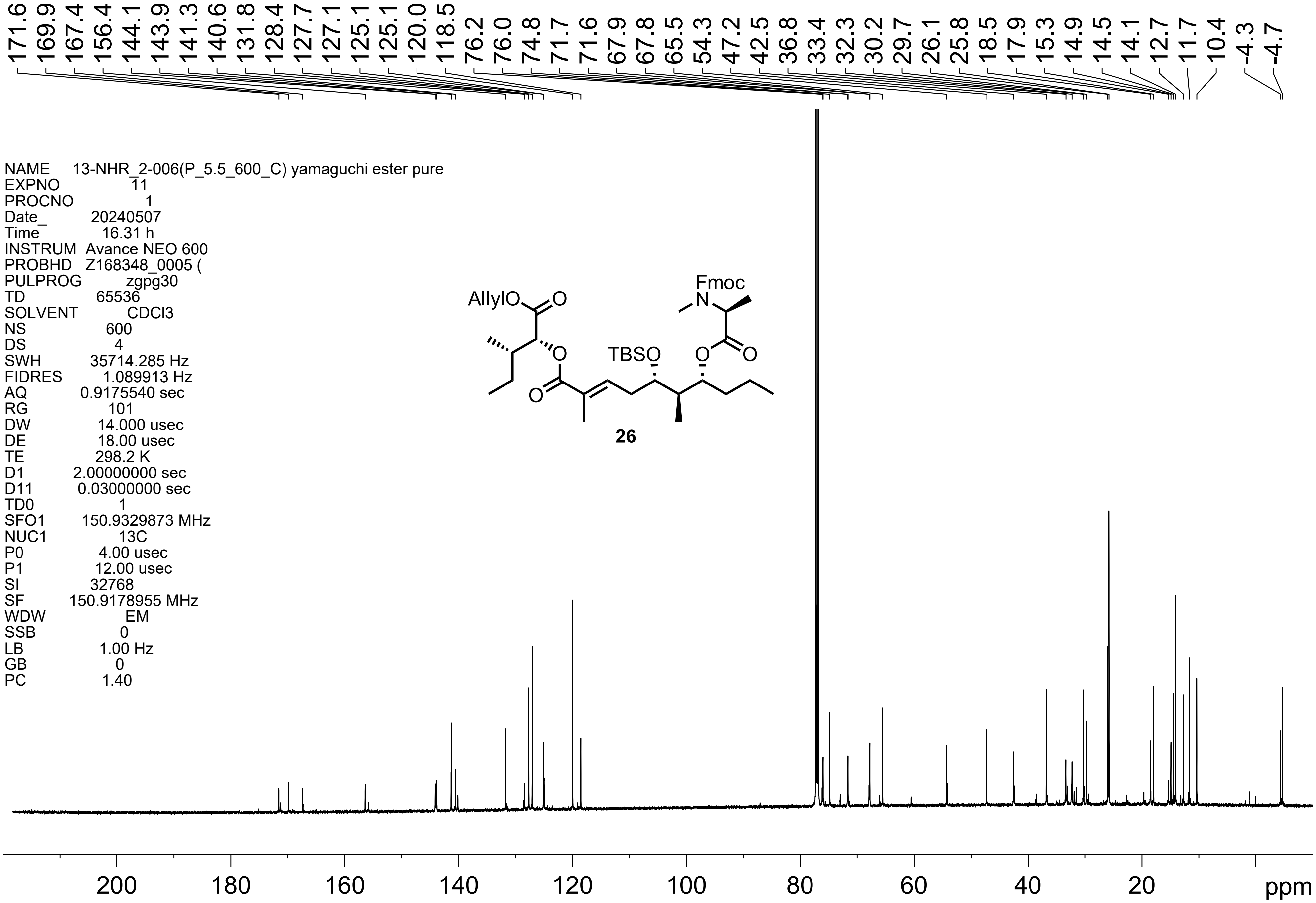

NAME 13-NHR_2-006(P_5.5_600_C) yamaguchi ester pure
EXPNO 11
PROCNO 1
Date_ 20240507
Time 16.31 h
INSTRUM Avance NEO 600
PROBHD Z168348_0005 (
PULPROG zgpg30
TD 65536
SOLVENT CDCl3
NS 600
DS 4
SWH 35714.285 Hz
FIDRES 1.089913 Hz
AQ 0.9175540 sec
RG 101
DW 14.000 usec
DE 18.00 usec
TE 298.2 K
D1 2.00000000 sec
D11 0.03000000 sec
TD0 1
SFO1 150.9329873 MHz
NUC1 13C
P0 4.00 usec
P1 12.00 usec
SI 32768
SF 150.9178955 MHz
WDW EM
SSB 0
LB 1.00 Hz
GB 0
PC 1.40

26

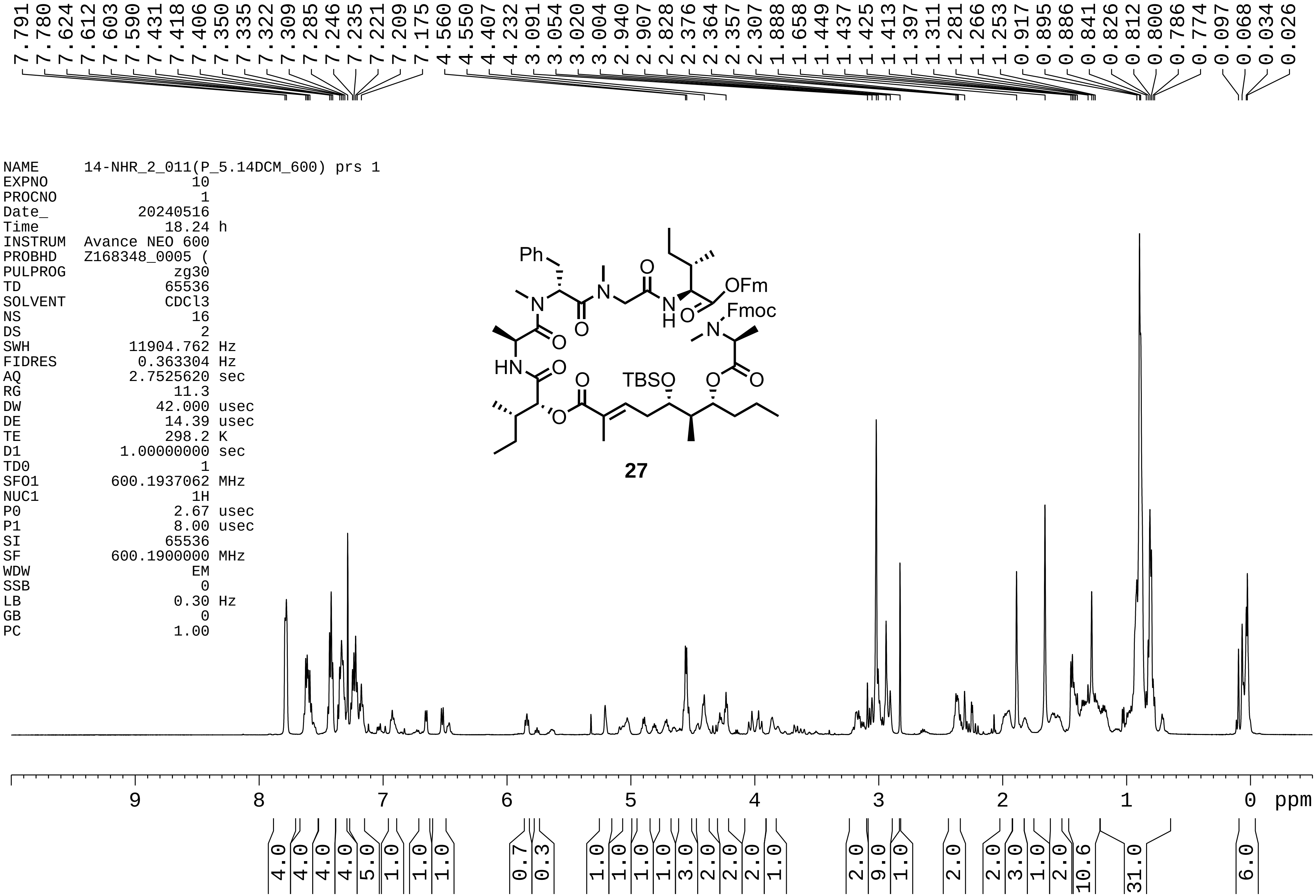

NAME 14-NHR_2_011(P_5.14DCM_600) prs 1
EXPNO 10
PROCNO 1
Date_ 20240516
Time 18.24 h
INSTRUM Avance NEO 600
PROBHD Z168348_0005 (
PULPROG zg30
TD 65536
SOLVENT CDCl3
NS 16
DS 2
SWH 11904.762 Hz
FIDRES 0.363304 Hz
AQ 2.7525620 sec
RG 11.3
DW 42.000 usec
DE 14.39 usec
TE 298.2 K
D1 1.00000000 sec
TD0 1
SFO1 600.1937062 MHz
NUC1 1H
P0 2.67 usec
P1 8.00 usec
SI 65536
SF 600.1900000 MHz
WDW EM
SSB 0
LB 0.30 Hz
GB 0
PC 1.00

27

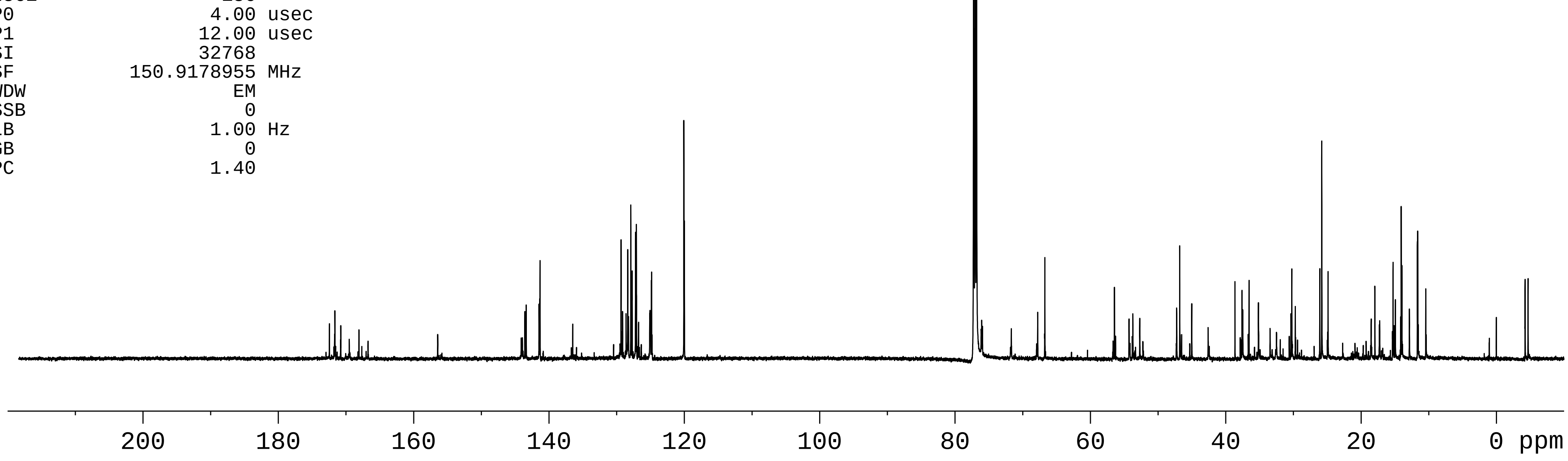

NAME 14-NHR_2_011(P_5.14DCM_600) prs 1
EXPNO 11
PROCNO 1
Date_ 20240516
Time 22.04 h
INSTRUM Avance NEO 600
PROBHD Z168348_0005 (
PULPROG zgpg30
TD 65536
SOLVENT CDCl3
NS 4400
DS 4
SWH 35714.285 Hz
FIDRES 1.089913 Hz
AQ 0.9175540 sec
RG 101
DW 14.000 usec
DE 18.00 usec
TE 298.2 K
D1 2.00000000 sec
D11 0.03000000 sec
TD0 1
SFO1 150.9329873 MHz
NUC1 13C
P0 4.00 usec
P1 12.00 usec
SI 32768
SF 150.9178955 MHz
WDW EM
SSB 0
LB 1.00 Hz
GB 0
PC 1.40

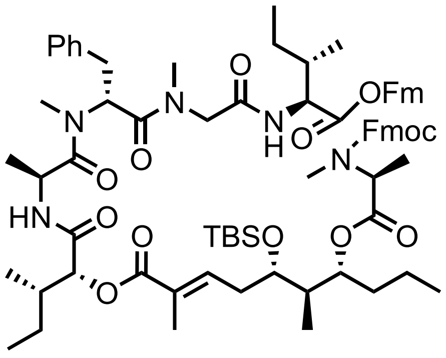

27

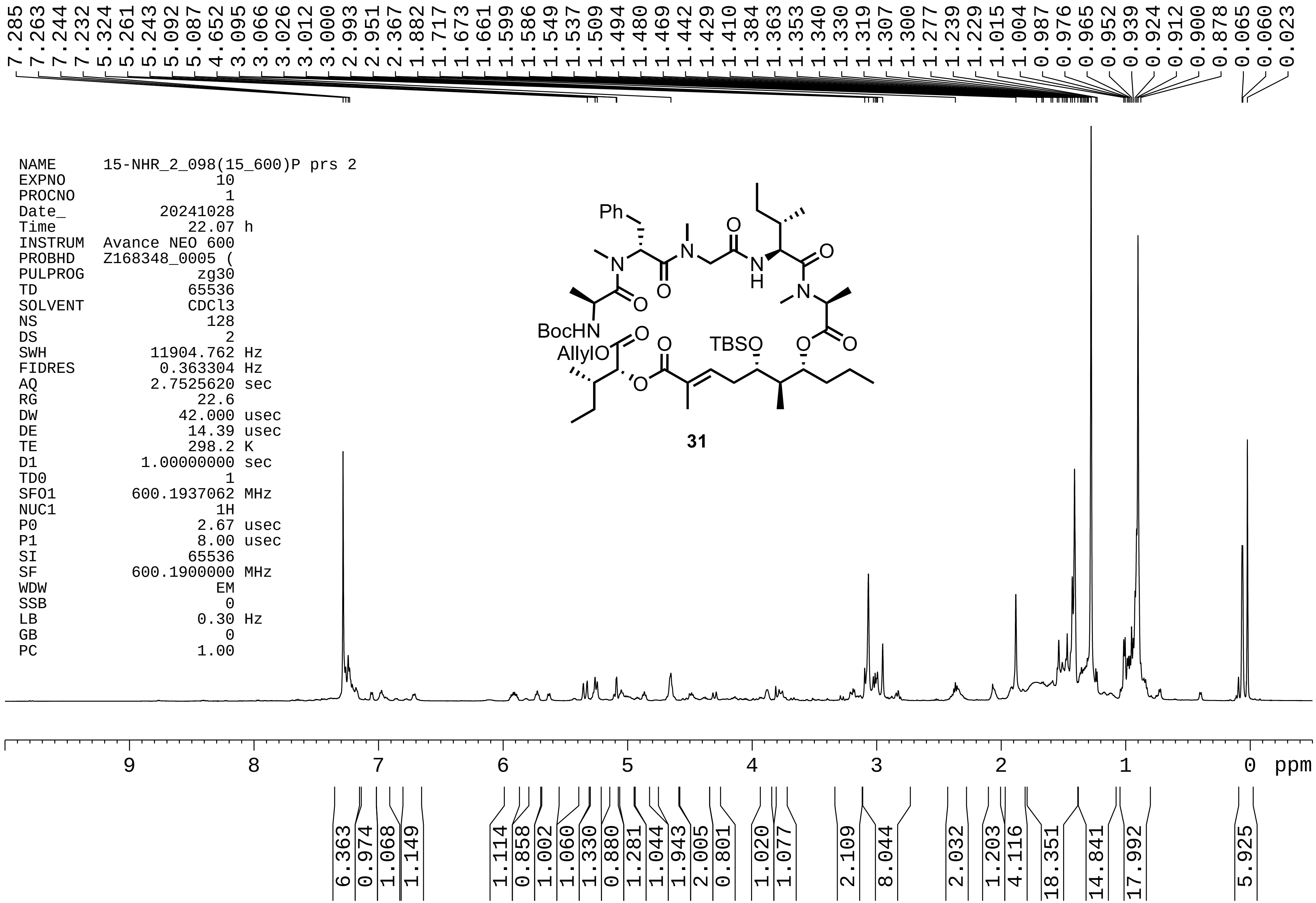
7.285 7.263 7.244 7.232 5.324 5.261 5.243 5.092 5.087 4.652 3.095 3.066 3.026 3.012 3.000 2.993 2.951 2.367 1.882 1.717 1.673 1.661 1.599 1.586 1.549 1.537 1.509 1.494 1.480 1.469 1.442 1.429 1.410 1.384 1.363 1.353 1.340 1.330 1.319 1.307 1.300 1.277 1.239 1.229 1.015 1.004 0.987 0.976 0.965 0.952 0.939 0.924 0.912 0.900 0.878 0.065 0.060 0.023
NAME 15-NHR_2_098(15_600)P prs 2
EXPNO 10
PROCNO 1
Date_ 20241028
Time 22.07 h
INSTRUM Avance NEO 600
PROBHD Z168348_0005 (
PULPROG zg30
TD 65536
SOLVENT CDCl3
NS 128
DS 2
SWH 11904.762 Hz
FIDRES 0.363304 Hz
AQ 2.7525620 sec
RG 22.6
DW 42.000 usec
DE 14.39 usec
TE 298.2 K
D1 1.00000000 sec
TD0 1
SFO1 600.1937062 MHz
NUC1 1H
P0 2.67 usec
P1 8.00 usec
SI 65536
SF 600.1900000 MHz
WDW EM
SSB 0
LB 0.30 Hz
GB 0
PC 1.00
Ph
BocHN
AllylO
TBSO
31
9 8 7 6 5 4 3 2 1 0 ppm
6.363 0.974 1.068 1.149 1.114 0.858 1.002 1.060 1.330 0.880 1.281 1.044 1.943 2.005 0.801 1.020 1.077 2.109 8.044 2.032 1.203 4.116 18.351 14.841 17.992 5.925

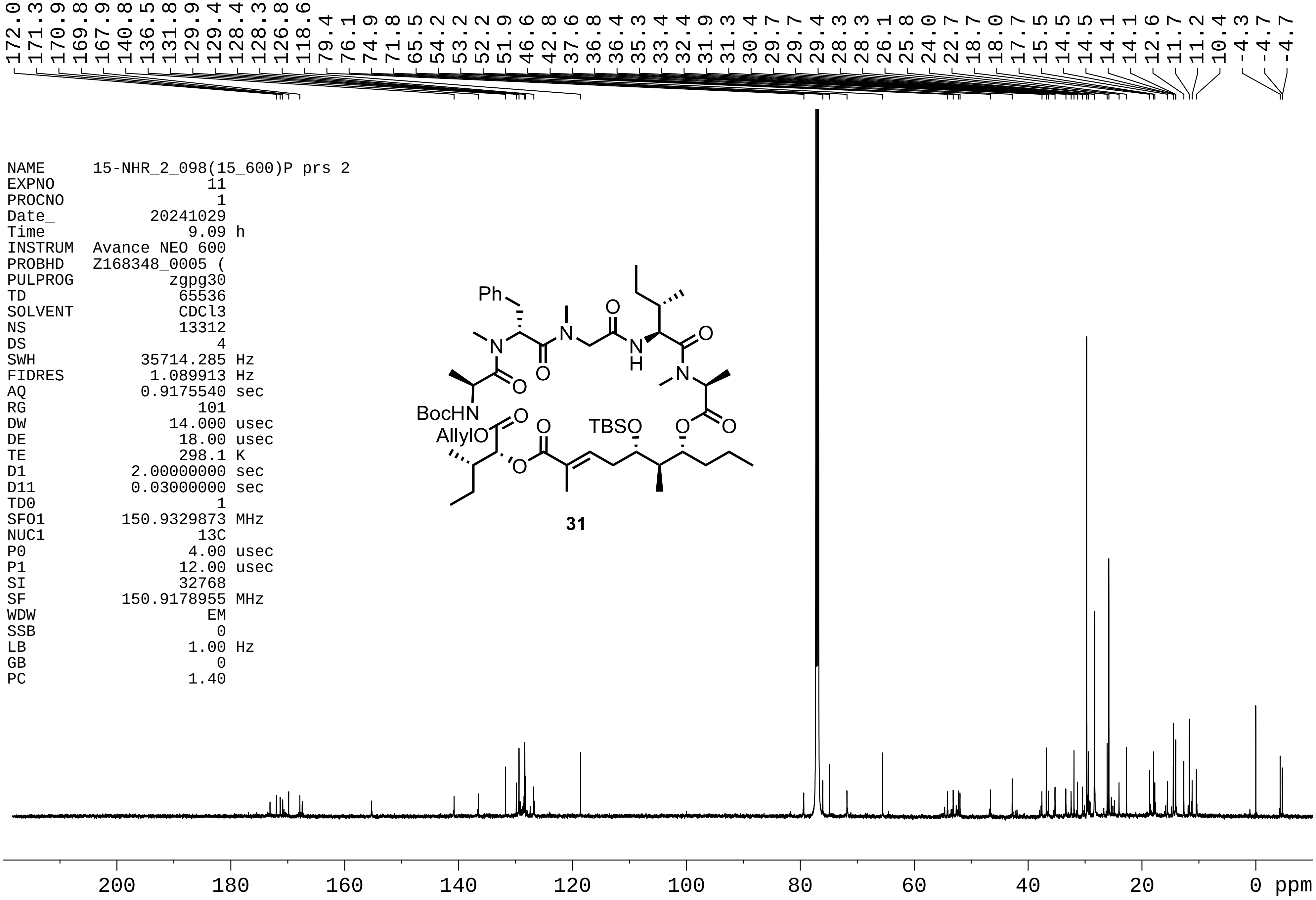
172.0
171.3
170.9
169.8
167.9
140.8
136.5
131.8
129.9
129.4
128.4
128.3
126.8
118.6
79.4
76.1
74.9
71.8
65.5
54.2
53.2
52.2
51.9
46.6
42.8
37.6
36.8
36.4
35.3
33.4
32.4
31.9
31.3
30.4
29.7
29.7
29.4
28.3
28.3
26.1
25.8
24.0
22.7
18.7
18.0
17.7
15.5
14.5
14.5
14.1
14.1
12.6
11.7
11.2
10.4
-4.3
-4.7
-4.7
NAME 15-NHR_2_098(15_600)P prs 2
EXPNO 11
PROCNO 1
Date_ 20241029
Time 9.09 h
INSTRUM Avance NEO 600
PROBHD Z168348_0005 (
PULPROG zgpg30
TD 65536
SOLVENT CDCl3
NS 13312
DS 4
SWH 35714.285 Hz
FIDRES 1.089913 Hz
AQ 0.9175540 sec
RG 101
DW 14.000 usec
DE 18.00 usec
TE 298.1 K
D1 2.00000000 sec
D11 0.03000000 sec
TD0 1
SFO1 150.9329873 MHz
NUC1 13C
P0 4.00 usec
P1 12.00 usec
SI 32768
SF 150.9178955 MHz
WDW EM
SSB 0
LB 1.00 Hz
GB 0
PC 1.40
Ph
O
N
H
O
N
O
N
O
BocHN
O
AllylO
O
TBSO
O
O
O
O
31
200
180
160
140
120
100
80
60
40
20
0 ppm

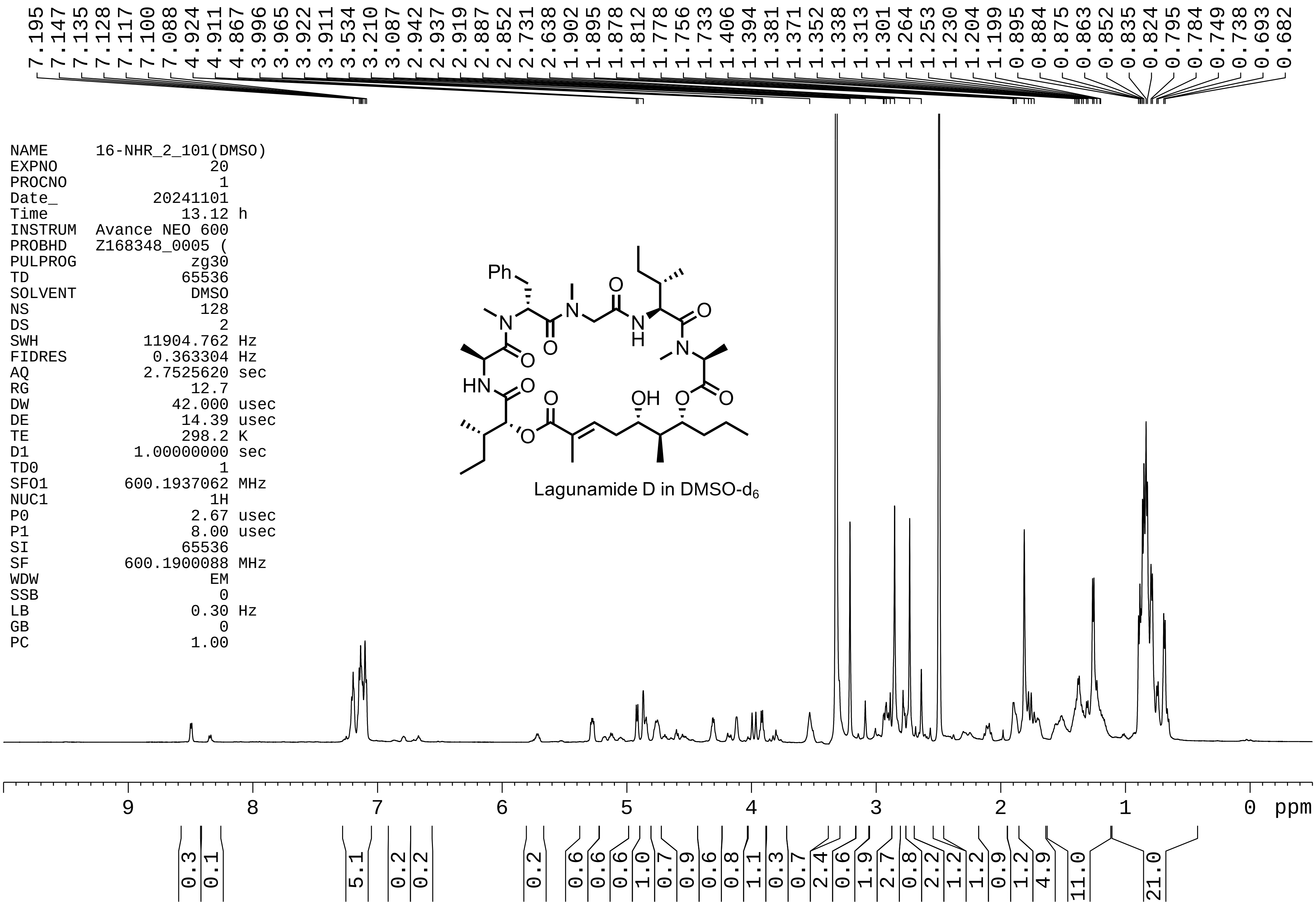
7.195
7.147
7.135
7.128
7.117
7.100
7.088
4.924
4.911
4.867
3.996
3.965
3.922
3.911
3.534
3.210
3.087
2.942
2.937
2.919
2.887
2.852
2.731
2.638
1.902
1.895
1.878
1.812
1.778
1.756
1.733
1.406
1.394
1.381
1.371
1.352
1.338
1.313
1.301
1.264
1.253
1.230
1.204
1.199
0.895
0.884
0.875
0.863
0.852
0.835
0.824
0.795
0.784
0.749
0.738
0.693
0.682
NAME 16-NHR_2_101(DMSO)
EXPNO 20
PROCNO 1
Date_ 20241101
Time 13.12 h
INSTRUM Avance NEO 600
PROBHD Z168348_0005 (
PULPROG zg30
TD 65536
SOLVENT DMSO
NS 128
DS 2
SWH 11904.762 Hz
FIDRES 0.363304 Hz
AQ 2.7525620 sec
RG 12.7
DW 42.000 usec
DE 14.39 usec
TE 298.2 K
D1 1.00000000 sec
TD0 1
SFO1 600.1937062 MHz
NUC1 1H
P0 2.67 usec
P1 8.00 usec
SI 65536
SF 600.1900088 MHz
WDW EM
SSB 0
LB 0.30 Hz
GB 0
PC 1.00
Ph
N
O
H
HN
OH
Lagunamide D in DMSO-d_6
9
8
7
6
5
4
3
2
1
0
ppm
0.3
0.1
5.1
0.2
0.2
0.2
0.6
0.6
0.6
1.0
0.7
0.9
0.6
0.8
1.1
0.3
0.7
2.4
0.6
1.9
2.7
0.8
2.2
1.2
1.2
0.9
1.2
4.9
11.0
21.0

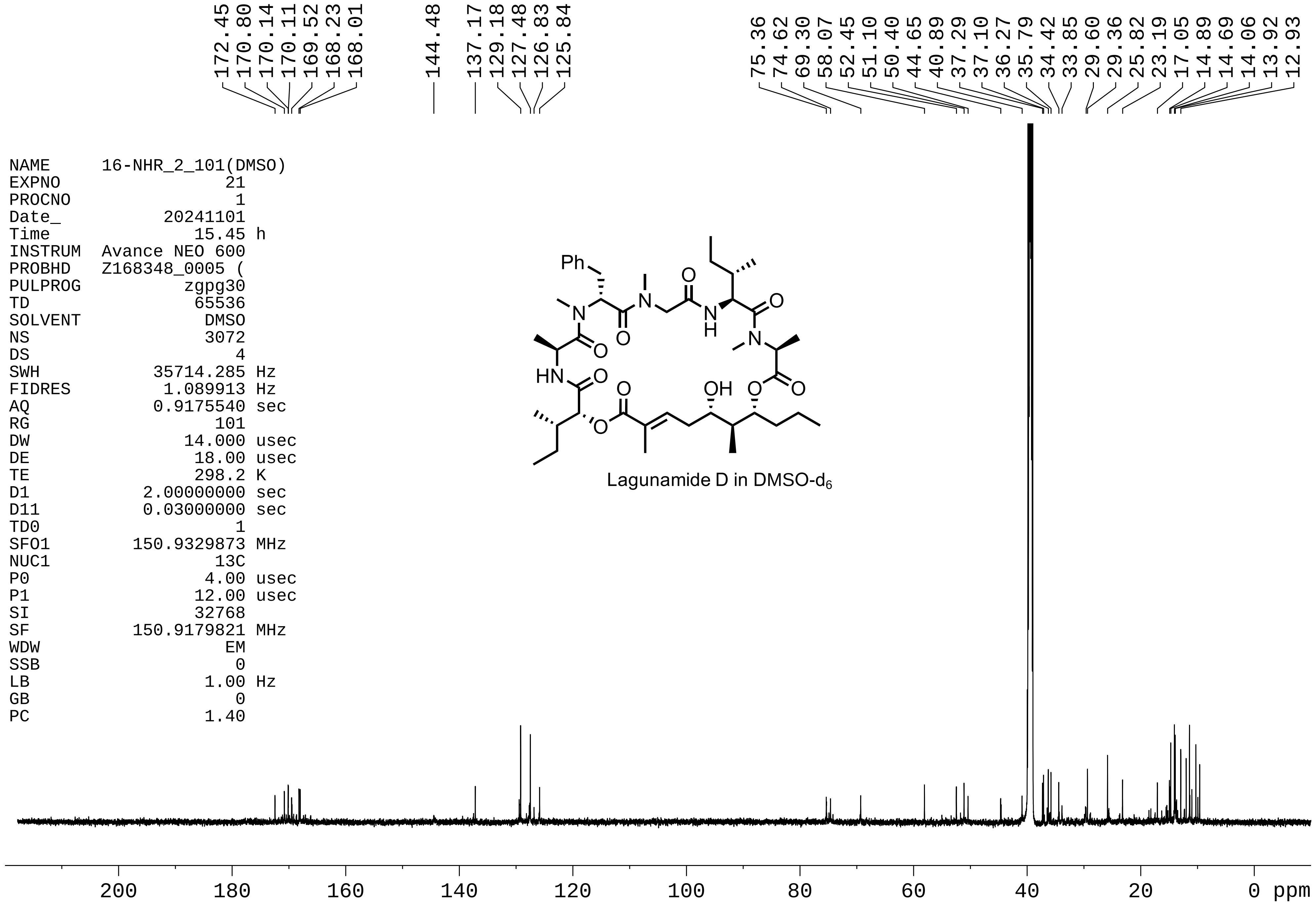
172.45
170.80
170.14
170.11
169.52
168.23
168.01
144.48
137.17
129.18
127.48
126.83
125.84
75.36
74.62
69.30
58.07
52.45
51.10
50.40
44.65
40.89
37.29
37.10
36.27
35.79
34.42
33.85
29.60
29.36
25.82
23.19
17.05
14.89
14.69
14.06
13.92
12.93
NAME 16-NHR_2_101(DMSO)
EXPNO 21
PROCNO 1
Date_ 20241101
Time 15.45 h
INSTRUM Avance NEO 600
PROBHD Z168348_0005 (
PULPROG zgpg30
TD 65536
SOLVENT DMSO
NS 3072
DS 4
SWH 35714.285 Hz
FIDRES 1.089913 Hz
AQ 0.9175540 sec
RG 101
DW 14.000 usec
DE 18.00 usec
TE 298.2 K
D1 2.00000000 sec
D11 0.03000000 sec
TD0 1
SFO1 150.9329873 MHz
NUC1 13C
P0 4.00 usec
P1 12.00 usec
SI 32768
SF 150.9179821 MHz
WDW EM
SSB 0
LB 1.00 Hz
GB 0
PC 1.40
Ph
N
O
HN
OH
Lagunamide D in DMSO-d_6
200
180
160
140
120
100
80
60
40
20
0 ppm

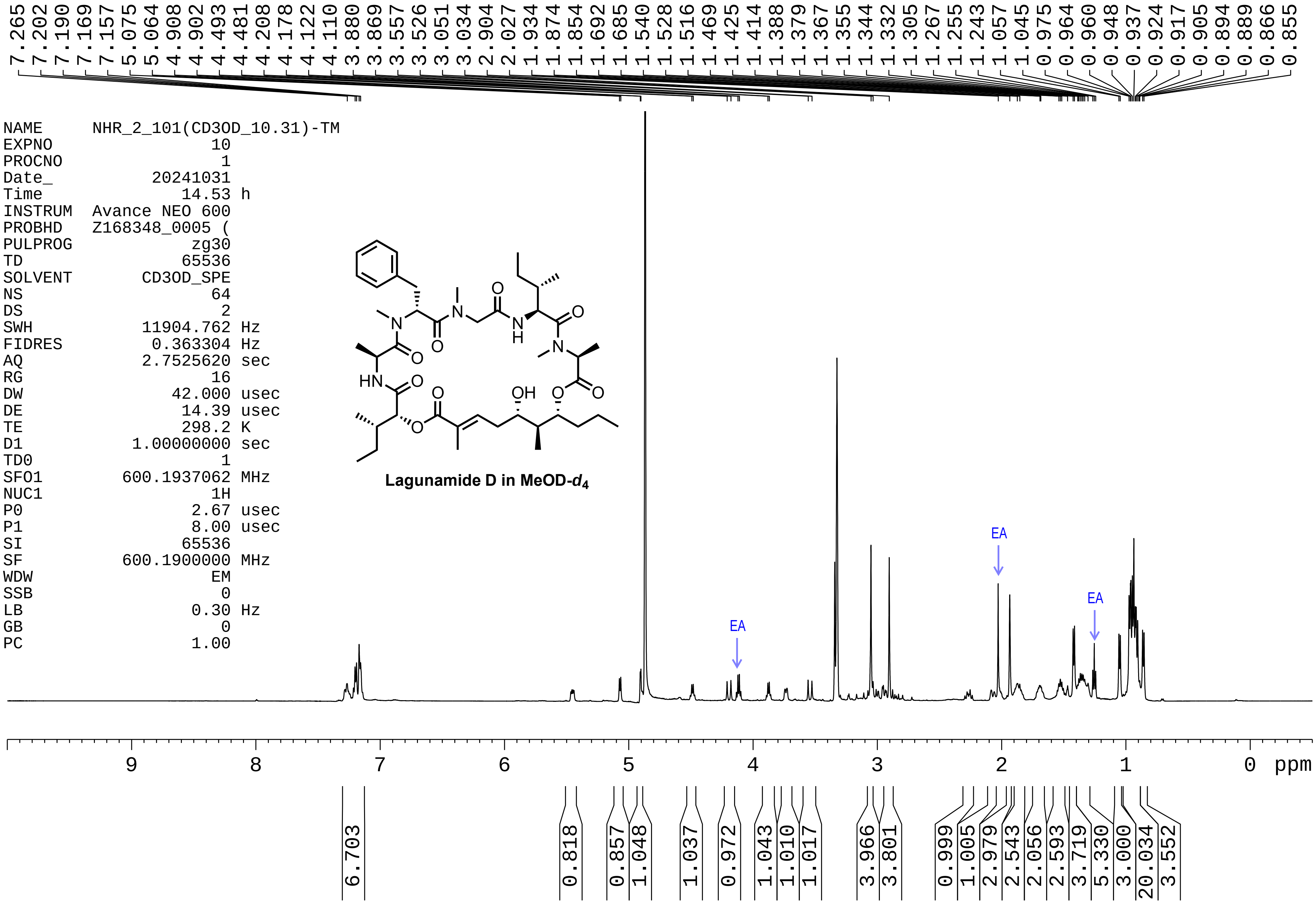

Lagunamide D in MeOD-d_4

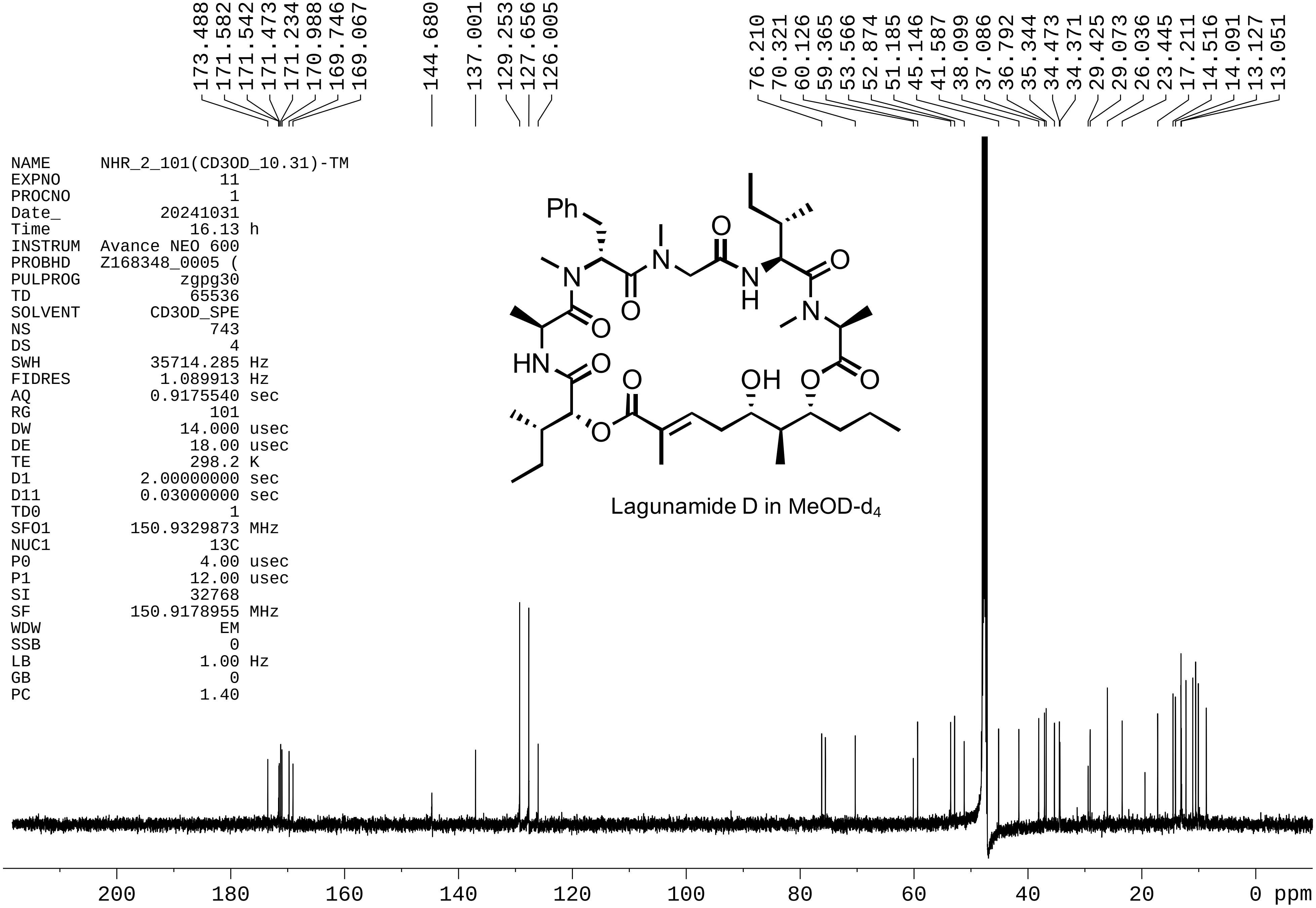
173.488
171.582
171.542
171.473
171.234
170.988
169.746
169.067
144.680
137.001
129.253
127.656
126.005
76.210
70.321
60.126
59.365
53.566
52.874
51.185
45.146
41.587
38.099
37.086
36.792
35.344
34.473
34.371
29.425
29.073
26.036
23.445
17.211
14.516
14.091
13.127
13.051
NAME NHR_2_101(CD3OD_10.31)-TM
EXPNO 11
PROCNO 1
Date_ 20241031
Time 16.13 h
INSTRUM Avance NEO 600
PROBHD Z168348_0005 (
PULPROG zgpg30
TD 65536
SOLVENT CD3OD_SPE
NS 743
DS 4
SWH 35714.285 Hz
FIDRES 1.089913 Hz
AQ 0.9175540 sec
RG 101
DW 14.000 usec
DE 18.00 usec
TE 298.2 K
D1 2.00000000 sec
D11 0.03000000 sec
TD0 1
SFO1 150.9329873 MHz
NUC1 13C
P0 4.00 usec
P1 12.00 usec
SI 32768
SF 150.9178955 MHz
WDW EM
SSB 0
LB 1.00 Hz
GB 0
PC 1.40
Ph
O
N
H
HN
OH
Lagunamide D in MeOD-d4
200
180
160
140
120
100
80
60
40
20
0 ppm

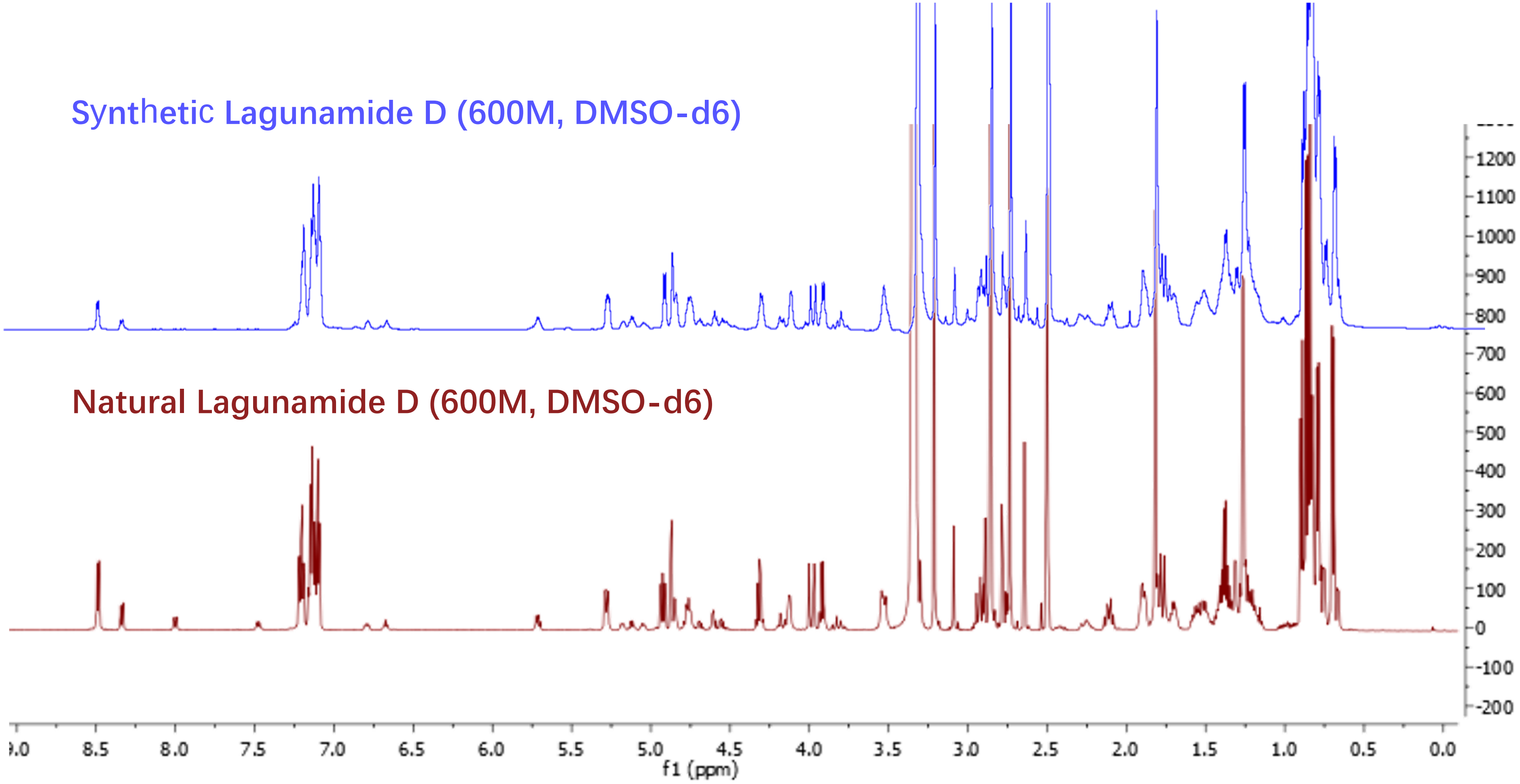

3. Figure S1. Comparison of ^{1}H NMR (600 MHz) for Natural [1] and Synthetic Lagunaminde D in DMSO-*d6*.

References

1. Luo, D.; Putra, M. Y.; Ye, T.; Paul, V. J.; Luesch, H., Isolation, Structure Elucidation and Biological Evaluation of Lagunamide D: A New Cytotoxic Macrocyclic Depsipeptide from Marine Cyanobacteria. *Mar. Drugs* **2019**, *17*, 83.
